# Bioorthogonal site-selective conjugation of fluorescent dyes to antibodies: method and potential applications†

**DOI:** 10.1039/d2ra05580e

**Published:** 2022-10-05

**Authors:** Philipp Grossenbacher, Maria C. Essers, Joël Moser, Simon A. Singer, Stephanie Häusler, Bruno Stieger, Jean-Sébastien Rougier, Martin Lochner

**Affiliations:** Institute of Biochemistry and Molecular Medicine, University of Bern Bühlstrasse 28 3012 Bern Switzerland martin.lochner@ibmm.unibe.ch; Department of Chemistry, Biochemistry and Pharmaceutical Sciences, University of Bern Freiestrasse 3 3012 Bern Switzerland; Department of Clinical Pharmacology and Toxicology, University Hospital Zürich, University of Zürich Rämistrasse 100 8091 Zürich Switzerland

## Abstract

Antibodies are immensely useful tools for biochemical research and have found application in numerous protein detection and purification methods. Moreover, monoclonal antibodies are increasingly utilised as therapeutics or, conjugated to active pharmaceutical ingredients, in targeted chemotherapy. Several reagents and protocols are reported to synthesise fluorescent antibodies for protein target detection and immunofluorescence applications. However, most of these protocols lead to non-selective conjugation, over-labelling or in the worst case antigen binding site modification. Here, we have used the antibody disulphide cleavage and re-bridging strategy to introduce bright fluorescent dyes without loss of the antibody function. The resulting fluorescent IgG1 type antibodies were shown to be effective imaging tools in western blot and direct immunofluorescence experiments.

## Abstract 2

Several synthetic fluorescent dibromopyridazinediones were conjugated site-selectively to IgG1 antibodies to generate imaging tools for western blot and immunofluorescence applications.

## Graphical abstract

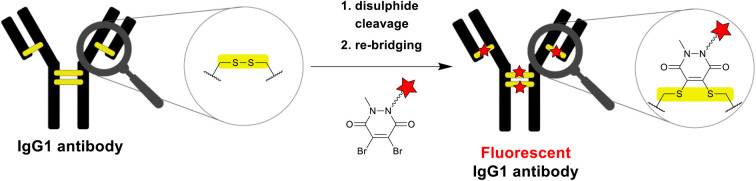

## Introduction

The use of antibody drug conjugates (ADCs) has seen a substantial rise in recent times. They offer precise treatment options with fewer side effects, as the payload is guided by the antibody to its specific target.^1^ In the wake of this development, various techniques have been established, moving from highly heterogenic mixtures to precise homogenous conjugate constructs using monoclonal antibodies with specified drug-to-antibody ratios (DARs) and controlled sites of attachment. For instance, unnatural amino acids can be used to engineer bioorthogonal functional groups into the monoclonal antibody, which subsequently enable site-selective and efficient coupling reactions with a variety of modified drugs.^2^

Traditionally, native antibody modifications have relied on the chemical reactivity of amino acid side chains, and numerous chemical reagents and methods have been developed to conjugate drug molecules to such reactive groups (*e.g.* the primary amine group of lysine residues).^3^ Such chemical reagent-based methods often produce heterogeneous mixtures of modified antibodies and involve the risk of over-labelling and altering the antigen binding sites, which results in inactive antibody conjugates. Alternatively, more recent strategies entail the targeting of glycans in native antibodies to guide modifications away from the antigen binding sites.^4^ Thiols are useful functional groups for protein bioconjugation as they react efficiently with widely used and available maleimides. Thiol-containing antibodies can be obtained through cysteine-engineering^5^ or generated by full or partial reduction of disulphide bonds, and reacted site-specifically with maleimide reagents.^2^ A potential drawback of maleimide conjugates is their propensity to undergo retro-Michael addition reactions and transfer their payload onto plasma thiols.^6^ Another viable approach is to specifically target the interchain disulphide bonds, in particular in IgG1 antibodies. In this subtype, four solvent-accessible disulphide bonds can be cleaved by mild, biocompatible reducing agents (*e.g.* TCEP or DTT) and then re-bridged using various bis-reactive cysteine reagents that carry the payload or functional groups for subsequent conjugation reactions.^2,4^ Several re-bridging strategies and agents have been developed, including bissulfones,^7,8^ divinylsulfonamides,^9^ arylene dipropiolonitriles (ADPN),^10^ dibromomethyl heterocycles (C-Lock™),^11^ dichloroacetone,^12^ next-generation maleimides,^13–15^ pyridazinediones^16–22^ and divinylpyrimidines.^23–25^

Immunofluorescence applications in general require fluorescently labelled antibodies. Detection of the cellular target can either be achieved directly, using a primary labelled antibody, or indirectly, by using a secondary labelled antibody that recognises the primary (unlabelled) one. Numerous commercial suppliers offer primary or secondary fluorescently labelled antibodies that were raised in different species. The selection of the primary antibody is one of the most important steps to achieve successful experimental outcome in western blot and immunofluorescence applications. Despite the wide variety of commercial antibodies, in many cases antibodies against a specific protein of interest are not available and first have to be generated, validated, and depending on the intended application, fluorescently labelled.

The common workflow in immunofluorescence includes the use of a primary antibody that binds specifically to the cellular target, followed by detection of the primary antibody with a fluorescently labelled secondary antibody. While this method works for most applications, there are limitations that negatively affect experimental outcome (Table 1). Direct immunofluorescence can provide an alternative in such situations; however, it does not come without technical issues either (Table 1).

**Table 1:** Advantages and disadvantages of direct and indirect immunofluorescence

Method	Advantages	Disadvantages
Direct immunofluorescence	• Simplified workflow and shorter sample staining time	• Lower sensitivity for low-abundance targets with limited number of fluorescent dyes that can be attached to primary antibody
	• No cross-reactivity between secondary antibodies	• Limited number of commercially available fluorescently labelled primary antibodies
	• Best solution for specific staining of multiple targets	
Indirect immunofluorescence	• Greater sensitivity for low-abundance targets due to signal amplification by secondary antibodies	• Cross-reactivity between secondary antibodies, in particular when performing multilabel experiments
	• Numerous inexpensive secondary antibodies commercially available with various dyes and tags attached	• Reaction of secondary antibodies with endogenous immunoglobulins leading to high background fluorescence

The goal of our study was to provide a practical method to site-specifically label native antibodies with bright fluorescent dyes by targeting the solvent-accessible interchain disulphide bonds. In most examples such conjugations have been shown on monoclonal IgG1 therapeutic antibody trastuzumab (Herceptin®),^9,13–21,24^ and recently monoclonal IgG4 magacizumab.^22^ In this work, we explored if the modification approach is more widely applicable to other mono- and polyclonal IgG1 antibodies and validated the fluorescently labelled products in western blot and direct immunofluorescence applications.

## Results and discussion

### Chemistry

**Scheme 1:**
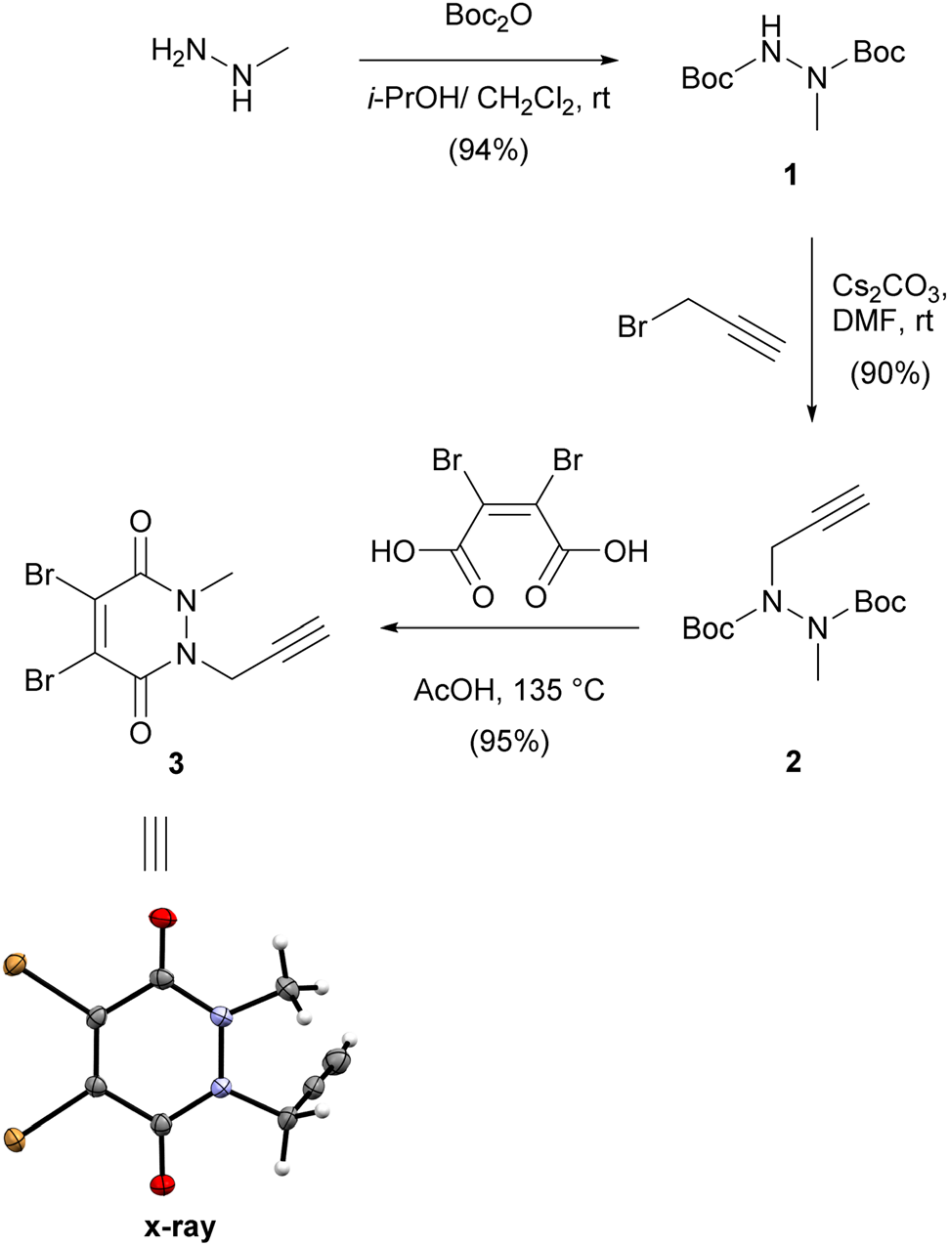
Synthesis of propargyl dibromopyridazinedione 3 and X-ray structure thereof.

We first synthesised small and versatile re-bridging agent 3, featuring an alkyne clickable handle for fluorescent dye conjugation, by adapting synthetic routes published for very similar dibromopyridazinediones.^18,20,26,27^ Methylhydrazine was first Boc-protected and then alkylated with propargyl bromide on the remaining free carbamate nitrogen (Scheme 1).

This was followed by a one-pot deprotection and cyclisation reaction by heating bis-carbamate 2 with dibromomaleic acid in acetic acid at refluxing temperature.^20^ Overall, this synthetic route delivered dibromopyridazinedione 3 in 80% yield over three steps as a crystalline solid. We were able to obtain a crystal structure of 3 to confirm the identity of the compound (Scheme 1).

**Scheme 2:**
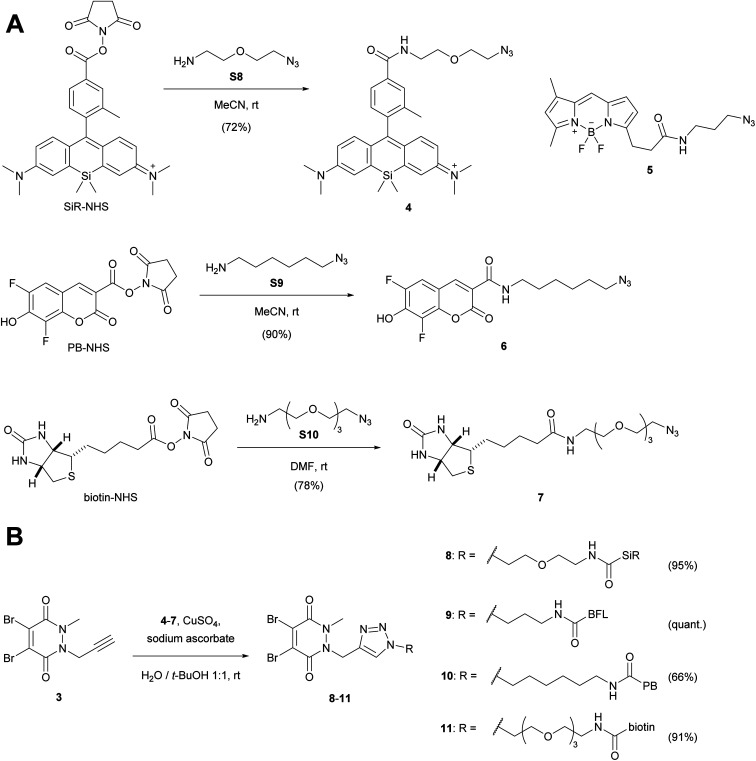
(A) Attachment of linkers to fluorescent dyes and biotin. (B) CuAAC-coupling with alkyne dibromopyridazinedione 3. For the synthesis of SiR-NHS (Scheme S1†), biotin-NHS and the azide linkers S8–S10 see ESI.† PB-NHS was commercially available. SiR, Si-rhodamine; PB, Pacific Blue; BFL, BODIPY FL.

We then attached various azido linkers *via* activated *N*-hydroxysuccinimid (NHS) esters to different fluorescent dyes that emit bright light in the red/far-red (Si-rhodamine, SiR)^28^ or blue region (Pacific Blue, PB), or to biotin (Scheme 2A). Azide 5 with a bright emission in the green region (BODIPY FL, BFL) was purchased from a commercial supplier.

The resulting constructs 4–7 were then coupled to pyridazinedione alkyne 3 using standard Cu-catalysed alkyne–azide cycloaddition (CuAAC) conditions, which gave the final re-bridging agents 8–11 in good to very good yields.

Originally, our synthetic plan was to couple the SiR dye with alternative dibromopyridazinediones *via* NHS-activated esters, rather than using CuAAC coupling chemistry (Scheme S2, ESI†). Even though the amide forming reactions were successful, one bromine atom was displaced by the NHS leaving group. We therefore speculate that this side reaction might be a potential generic risk when working with dibromopyridazinediones in the presence of NHS-activated esters, although these undesired products might still work as re-bridging agents.

### Anti GAPDH antibody modification

Glyceraldehyde 3-phosphate dehydrogenase (GAPDH) is a key enzyme in the glycolytic pathway. It catalyses the conversion of glyceraldehyde-3-phosphate to 1,3-biphopshoglycerate in the presence of NAD^+^ and inorganic phosphate. In biochemistry labs, GAPDH is commonly used as house-keeping gene in semi-quantitative or quantitative studies to confirm expression or suppression of proteins of interest in proteomic or genetic studies. Apart from its practical utility and important role in carbohydrate metabolism, several studies have shown that GAPDH also binds single-stranded DNA and in some cases acts as a transcription factor.^29^ Furthermore, GAPDH can initiate apoptosis but can also function as a mediator of cell survival. Other studies thus point to the fact that GAPDH could be an important marker in age-related neurodegenerative diseases, prostate cancer, and viral diseases.^30,31^

**Fig. 1:**
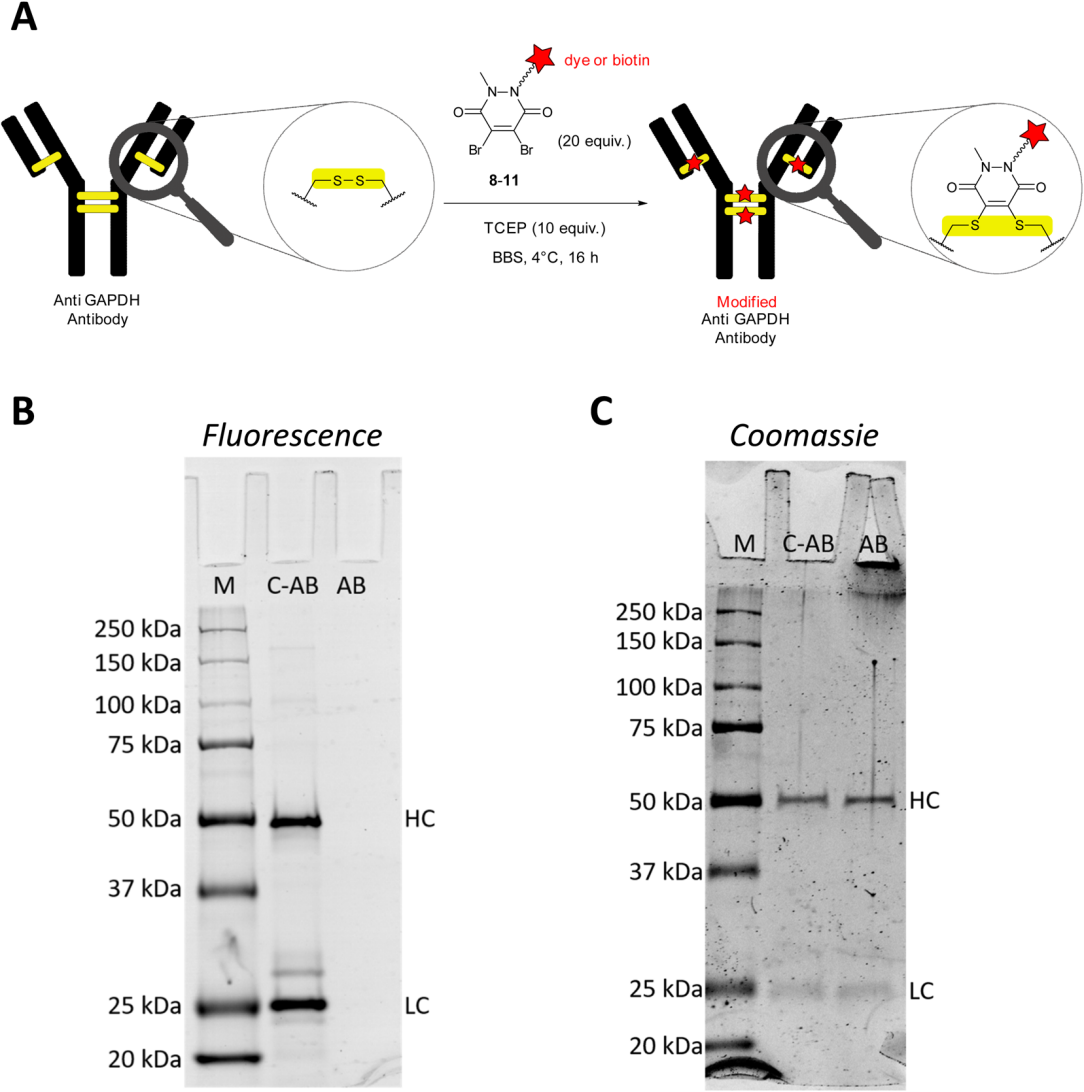
(A) Modification of anti GAPDH antibody by disulphide bond cleavage and re-bridging with dibromopyridazinediones 8–11. (B) Representative SDS-PAGE (reducing conditions) of conjugation reaction of anti GAPDH antibody with SiR-labelled 8. Detection of protein bands by fluorescence (excitation at 635 nm, Cy5 channel). Bands at 50 kDa and 25 kDa correspond to the heavy (HC) and light chains (LC), respectively. (C) SDS-PAGE as in (B), stained with Coomassie blue dye. Loading concentration of C-AB for in-gel fluorescence (B) was tenfold lower than for Coomassie gel (C) to avoid oversaturation of the fluorescence detector. Lanes: M, all Blue Protein Standard; C-AB, SiR-conjugated anti GAPDH antibody; AB, native anti GAPDH antibody.

For the initial antibody conjugation experiments, we chose a commercially available monoclonal anti GAPDH loading control antibody and followed the reported disulphide re-bridging protocol^20^ with some slight experimental modifications. The anti GAPDH antibody was incubated with TCEP (10 equiv.) and excess (20 equiv.) dibromopyridazinedione re-bridgers 8–11 in borate buffered saline (BBS) at 4 °C overnight (Fig. 1A). Isolation of the modified antibodies was conveniently achieved by using spin filter columns. Fig. 1B and C show a representative analysis of the SiR-modified anti GAPDH antibody by SDS-PAGE. Two strongly fluorescent bands at 25 kDa and 50 kDa were detected, which correspond to the fluorescently labelled light and heavy chains, respectively (Fig. 1B, lane C-AB).

During the reductive and denaturing SDS-PAGE sample preparation (using 10% mercaptoethanol in the loading buffer and heating), excess thiol presumably leads to disulphide cleavage and antibody fragmentation. The same two bands at 25 kDa and 50 kDa were also detected when a native anti GAPDH antibody sample was prepared analogously for SDS-PAGE and stained with Coomassie (Fig. 1C, lane AB). Omitting the mercaptoethanol in the SDS-PAGE loading buffer for the SiR-conjugated antibody sample resulted in several fluorescent bands at very high molecular weight at around 250 kDa (Fig. S1, ESI†), most probably resulting from incomplete linearisation and aggregation. We therefore deemed the non-reducing SDS-PAGE conditions as less suitable for conjugation reaction control. Due to the very bright fluorescence of the SiR dye, the conjugated antibody SDS-PAGE sample had to be diluted down tenfold to avoid oversaturation of the fluorescence detector (Fig. 1B). It is worth noting that at this low sample concentration Coomassie staining was almost unable to detect the same protein bands.

In addition to SDS-PAGE analysis, we have used size exclusion chromatography (SEC) to characterise the antibody conjugation products. The SEC traces of BFL-modified anti GAPDH antibody after re-bridging with dibromopyridazinedione 9 show the same peaks with very similar retention times as SEC traces of native anti GAPDH antibody (Fig. S2†). This leads us to conclude that the antibody remains intact and a fully re-bridged antibody is obtained. Consequently, it is more likely that the fragmentation of the modified antibodies occurs during the reductive SDS-PAGE sample preparation.

## Validation of modified GAPDH antibody and immunofluorescence

**Fig. 2:**
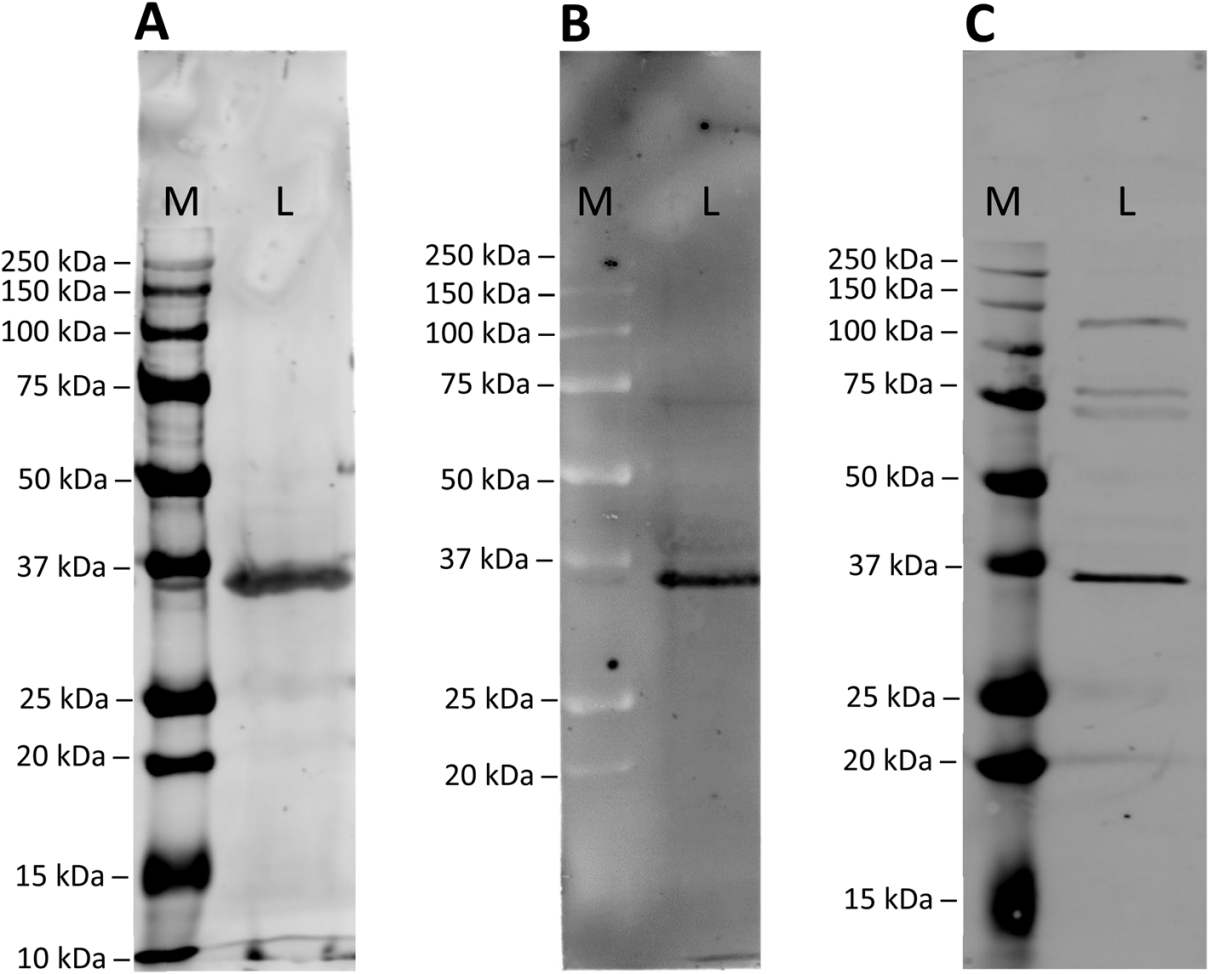
Western blot analysis of mouse cardiomyocyte whole cell lysate using (A) SiR-modified, (B) BFL-modified or (C) biotinylated anti GAPDH antibody. Antibodies were detected directly by fluorescence (A and B; Cy5 and FITC channel, respectively) or indirectly by complexing with streptaviding-Cy5 contstruct (C). The band at 36 kDa corresponds to the molecular weight of mouse GAPDH. Lanes: M, Marker; L, Lysate.

To assess the specificity of the modified anti GAPDH antibodies, they were tested in western blot applications, using mouse cardiomyocytes whole cell lysates (Fig. 2). After incubation and blotting, SiR- and BFL-modified antibodies were detected directly by fluorescence emission (Fig. 2A and B), whereas biotin-modified antibody was visualised by complexing with a streptavidin-Cy5 construct (Fig. 2C). In all cases a strong band at around 36 kDa was detected, which is in good agreement with the calculated molecular weight of mouse GAPDH (35.81 kDa).^32^

For comparison, we have employed a classical antibody modification method and stochastically modified anti GAPDH antibody with electrophilic, fluorescent reagent SiR-NHS. SDS-PAGE analysis of this directly labelled antibody product revealed the same fluorescent bands for the light and heavy chains (Fig. S3A, ESI†), as for SiR-modified anti GAPDH antibody obtained by re-bridging with dibromopyridazinedione 8 (Fig. 1B). It was noticeable, however, that the fluorescence intensity of the directly labelled antibody was markedly lower than of the re-bridged antibody. More importantly, the directly SiR-labelled antibody failed to detect its antigen GAPDH in whole cell lysate (Fig. S3B, ESI†).

**Fig. 3:**
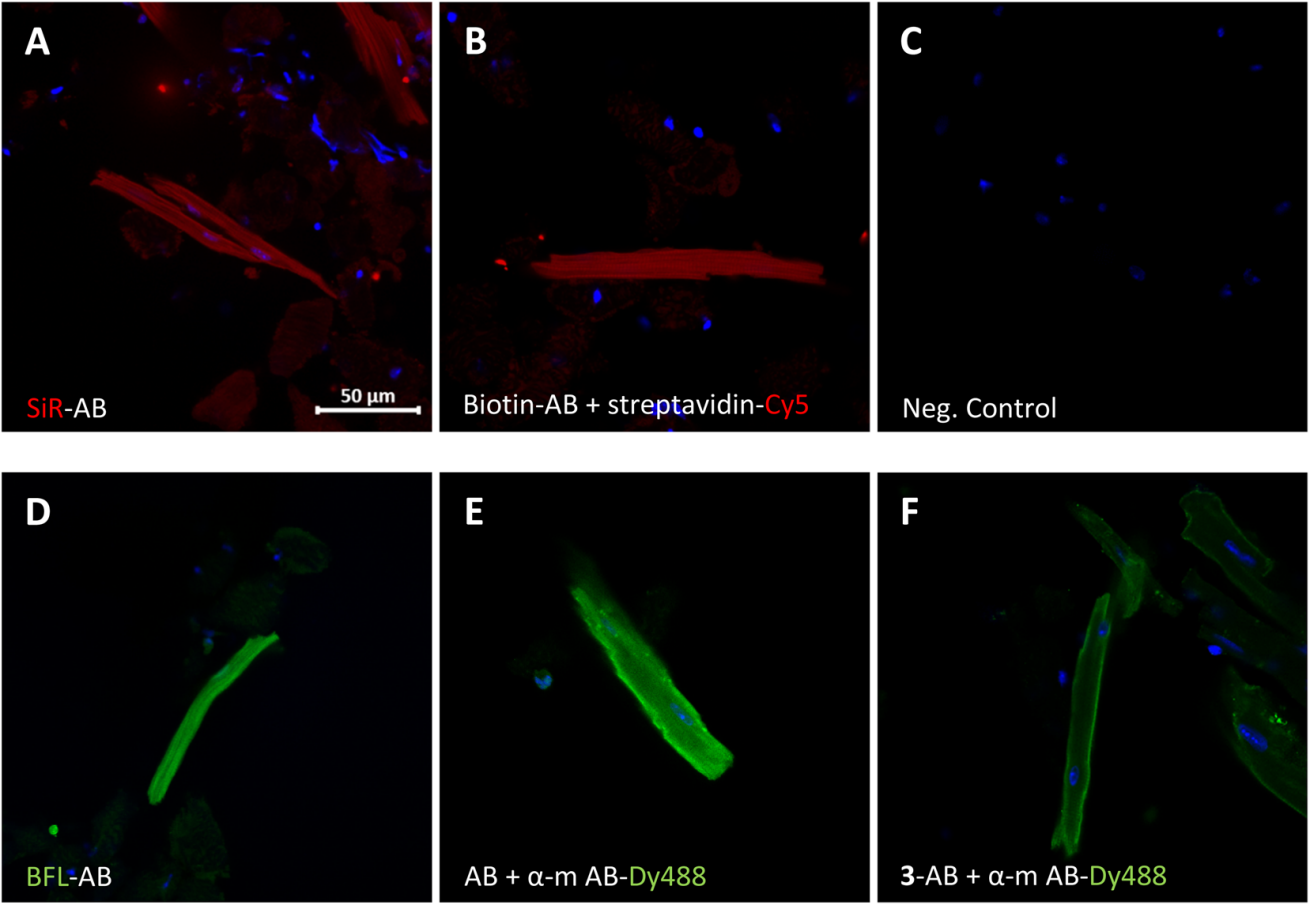
Direct (A and D) and indirect (B, E and F) immunofluorescence with isolated and fixed mouse cardiomyocytes using conjugated and native anti GAPDH antibodies. (A) SiR-modified and (B) biotinylated anti GAPDH antibody. (C) Negative control (DAPI and Cy5 Scan). (D) BFL-modified and (E) native anti GAPDH antibody. (F) Anti GAPDH antibody re-bridged with alkyne dibromopyridazinedione 3. Detection of antibodies by fluorescence (A and D; Cy5 and FITC channel, respectively), by complexing with streptaviding-Cy5 contstruct (B) or by secondary goat anti mouse Dy488-labelled antibodies (E and F). Cell nuclei were stained with DAPI. SiR, Si-rhodamine; BFL, BODIPY FL; Dy488, DyLight® 488; AB, anti GAPDH antibody; α-m AB, anti mouse antibody. Scale bar represents 50 μm.

The modified antibodies were then further assessed for their labelling utility in immunofluorescence experiments (Fig. 3). The images show isolated and fixed mouse cardiomyocytes incubated with modified and native anti GAPDH antibodies. Direct immunofluorescence with SiR- and BFL-conjugated antibodies clearly showed the cytosolic localisation of GAPDH in cardiomyocytes (Fig. 3A and D, respectively) compared to control (Fig. 3C). Alternatively, labelling with biotinylated antibody and subsequent incubation with streptavidin-Cy5 fusion construct gave the same staining pattern (Fig. 3B). Direct immunofluorescence staining was very similar to classical indirect immunofluorescence, using primary native anti GAPDH antibody and fluorescently labelled secondary anti mouse antibody (Fig. 3E). In another indirect immunofluorescence control experiment, we have used anti GAPDH antibody, which was re-bridged with alkyne dibromopyridazinedione 3, as primary antibody and a fluorescent secondary anti mouse antibody for staining (Fig. 3F). In this case the staining was again similar, albeit less strong, compared with direct (Fig. 3D) or indirect immunofluorescence (Fig. 3E).

### Modification of other IgG antibodies

We explored the general applicability of the fluorescence modification method with other commercially available or custom-generated IgG antibodies. The targeted antigens included a cytoskeletal protein (β-actin), cardiac ion channels (Na_v_1.5, Ca_v_1.2, Transient receptor potential cation channel subfamily M member 4 (TRPM4)) and a transmembrane ion pump (Na^+^/K^+^-ATPase). The respective IgG antibodies were conjugated with re-bridgers 8 or 9 according to the method shown in Fig. 1A and evaluated in western blot and direct immunofluorescence experiments (Table 2).

**Table 2:** Summary of produced IgG antibody conjugates and their evaluation in western blot and direct immunofluorescence experiments

Entry	Antigen	Antibody Type^a^	Tag^b^	Western Blot^c^	Direct Immunofluorescence*c*
1	GAPDH	IgG1 (m, mc)	SiR	Y	Y
2	GAPDH	IgG1 (m, mc)	BFL	Y	Y
3	GAPDH	IgG1 (m, mc)	Biotin	Y	Y
4	GAPDH	IgG1 (m, mc)	PB	—d	—d
5	β-Actin	IgG1 (m, mc)	SiR	Y	Y
6	Na_v_1.5	—e	BFL	N	Y
7	Ca_v_1.2	IgG^f^ (r, pc)	SiR	—g	Y
8	Na^+^/K^+^-ATPase	IgG1 (m, mc)	SiR	N	Y
9	TRPM4	IgG^f^ (r, pc)	SiR	Y	N^h^

a m, mouse; r, rabbit; mc, monoclonal; pc, polyclonal.

b SiR, Si-rhodamine; BFL, BODIPY FL, PB, Pacific Blue.

c Y, specific detection or staining of antigen; N, no detection or staining of antigen.

d Suitable detection system was not available.

e No information available from supplier.

f Subtype(s) not known.

g Western blot not attempted with SiR-modified antibody, as native antibody did not give signal.

h Native antibody did also not give specific staining in indirect immunofluorescence with secondary fluorescent antibody. Experiments in mouse heart sections showed similar inconclusive results for both SiR-modified and native anti TRPM4 antibodies.

In all examples, successful conjugation of fluorescent tags SiR or BFL was evident based on SDS-PAGE of the isolated antibody modification products (Fig. S4, ESI†). For instance, we obtained a SiR-modified anti β-actin antibody that was able to specifically detect its antigen in a cell lysate (entry 5) and gave very clear staining in direct immunofluorescence with mouse cardiomyocytes (Fig. S5 and S6, ESI†). Control indirect immunofluorescence using the native anti β-actin antibody and secondary Dy488-conjugated antibody produced very similar signal. For the studied ion channels and ion pump, the method failed to produce tools to detect Na_v_1.5, Ca_v_1.2 or Na^+^/K^+^-ATPase in cell lysate by western blot (entries 6–8). However, specific direct immunofluorescence signal was clearly obtained with BFL-modified anti Na_v_1.5, SiR-modified anti Ca_v_1.2 and anti Na^+^/K^+^-ATPase antibodies (Fig. S5 and S6, ESI†). It is noteworthy, that the anti Ca_v_1.2 staining tool was obtained from a custom-generated polyclonal antibody population. Conversely, modification of polyclonal anti TRPM4 antibody yielded a product that gave a strong and specific signal in western blot, but direct immunofluorescence was inconclusive (entry 9). In this particular case, TRPM4 detection by indirect immunofluorescence or fluorescence-assisted cell sorting (FACS), using native polyclonal anti TRPM4 antibody and secondary fluorescent antibody, was not conclusive either.

## Conclusions

The growing field of ADCs has produced several useful antibody conjugation protocols, some of which selectively target the interchain disulphide bridges. We have adapted the dithiol re-bridging dibromopyridazinedione reagents to introduce bright fluorescent dyes and biotin tags site-selectively into IgG1 type antibodies using a straightforward protocol. A further option, which we have not tested experimentally, would be to re-bridge with general reagent 3 and subsequently “click” the tag of choice to the alkyne-modified antibody.

The synthesised anti GAPDH antibody conjugates were shown to give strong and specific signals in western blot and direct immunofluorescence applications. Our produced modified anti GAPDH antibodies could be further evaluated as potential diagnostic tools in various disease models, where GAPDH was proposed as important marker for cell survival.^30,31^

It appears that the thiol re-bridging modification protocol is not generally transferable to other IgG1 antibodies and the source and quality of the native IgG1 antibody seems to play an important role. It is challenging to generate an imaging antibody tool that works equally well and gives specific labelling in both applications (western blot and direct immunofluorescence), and therefore, modified antibody products must be evaluated carefully for the applications intended. Nonetheless, we have shown that it is possible to start from commercially available monoclonal IgG1 and custom-produced polyclonal IgG antibodies, the latter of which consist of several subtypes having different number and locations of intra-chain disulphide bonds,^33,34^ and synthetically produce specific ion channel imaging tools for western blot (β-actin, TRPM4) and direct immunofluorescence (β-actin, Na_v_1.5, Ca_v_1.2, Na^+^/K^+^-ATPase) applications.

## Experimental section

### Chemistry

#### General remarks

All reactions requiring anhydrous conditions were performed in heat-gun, oven or flame dried glassware under inert atmosphere (N_2_, Ar). Silica gel 60 Å (40–63 mm) from Sigma-Aldrich was used for dry loads. Flash column chromatography was performed on a Teledyne Isco CombiFlash® Rf+ with the corresponding RediSep® prepacked silica cartouches unless otherwise stated. Thin layer chromatography (TLC) was performed on Machery & Nagel Alugram® xtra SIL G/UV 254 visualization under UV light (254 nm) and/or (366 nm) and/or by dipping in anisaldehyde stain and subsequent heating.

Commercial reagents and solvents (Acrôs Organics, Fluorochem, Grogg Chemie, Hänseler, Sigma-Aldrich) were used without further purification unless otherwise stated. Pacific Blue NHS ester and BODIPY FL azide 5 were obtained from Lumiprobe GmbH. Dry solvents for reactions were distilled and filtered over columns of dry neutral aluminium oxide under positive argon pressure. Solvents for extraction and flash chromatography were used without further purification.

^1^H and ^13^C NMR spectra were recorded on a Bruker AVANCE-300 or 400 spectrometers operating at 300 or 400 MHz for ^1^H and 75 or 101 MHz for ^13^C at room temperature unless otherwise stated. Chemical shifts (*δ*) are reported in parts per million (ppm) relative to tetramethylsilane (TMS) calibrated using residual signals of the solvent or TMS. Coupling constants (*J*) are reported in Hz. HRMS analyses and accurate mass determinations were performed on a Thermo Scientific LTQ Orbitrap XL mass spectrometer using ESI ionisation and positive or negative mode by the analytical services (mass spectrometry lab of Prof. Dr Stefan Schürch) from the Department of Chemistry, Biochemistry and Pharmaceutical Sciences (DCBP) of the University of Bern, Switzerland. HPLCs were measured on a Thermo-Scientific UltiMate 3000 HPLC with H_2_O + 0.1% TFA and MeCN + 0.1% TFA as eluents on an Acclaim™ 120 C 18 5 μm 120 Å (4.6 × 150 mm) column.

#### Di-*tert*-butyl 1-methylhydrazine-1,2-dicarboxylate (1)

To a stirred solution of methylhydrazine (5.7 mL, 5.00 g, 108.52 mmol) in 2-propanol (75 mL) at 27 °C Boc-anhydride (60.12 g, 275.48 mmol) was added dropwise over the course of 50 minutes. The mixture was stirred at 27 °C for 5 h and then concentrated under reduced pressure. The residue was taken up in EtOAc. The organic layer was washed with water, brine, dried over MgSO_4_, filtered and concentrated under reduced pressure. The crude was purified by flash column chromatography (cHex/EtOAc gradient from 1 : 0 to 0 : 1) to give the desired compound in 94% yield (25.02 g, 101.58 mmol). Physical and spectral data was in accordance with literature.^20^ Colourless solid: Major rotamer, ^1^H NMR (300 MHz, CDCl_3_) *δ* 6.47 (s, 1H), 3.08 (s, 3H), 1.45 (s, 18H). Minor rotamer, ^1^H NMR (300 MHz, CDCl_3_) *δ* 6.24 (s, 1H), 3.08 (s, 3H), 1.44 (s, 18H). Both rotamers: ^13^C NMR (75 MHz, CDCl_3_) *δ* 155.88, 81.27, 28.33. HRMS (ESI) calculated for [M + H]^+^ C_11_H_23_N_2_O_4_^+^ 247.1652, found 247.1658. Calculated for [M + Na]^+^ C_11_H_22_N_2_NaO_4_^+^ 269.1472, found 269.1477.

#### Di-*tert*-butyl 1-methyl-2-(prop-2-yn-1-yl)hydrazine-1,2-dicarboxylate (2)

To a stirred solution of di-*tert*-butyl 1-methylhydrazine-1,2-dicarboxylate (1) (0.51 g, 2.06 mmol) in DMF (20 mL) propargyl bromide (80% in toluene, 0.3 mL, 0.32 g, 2.69 mmol) and Cs_2_CO_3_ (1.04 g, 3.19 mmol) was added. The mixture was stirred at 23 °C under an argon atmosphere for 16 h. Then more propargyl bromide (80% in toluene, 0.3 mL, 0.32 g, 2.69 mmol) was added and the mixture was stirred for another 8 h. Subsequently, it was diluted with EtOAc and water. The aqueous phase was extracted with EtOAc. The combined organic layers were washed with brine (3*x*), dried over MgSO_4_, filtered and concentrated under reduced pressure. The residue was taken up in CH_2_Cl_2_ and was concentrated onto silica gel. The crude was purified by flash column chromatography (cylcohexane/EtOAc gradient from 1 : 0 to 9 : 1) to give the desired compound in 90% yield (0.53 g, 1.85 mmol). Colourless solid: Mix of rotamers, ^1^H NMR (300 MHz, DMSO-*d*_6_) *δ* 4.53–3.74 (m, 2H), 3.30–3.18 (m, 1H), 3.11–2.90 (m, 3H), 1.50–1.29 (m, 18H). ^13^C NMR (75 MHz, DMSO-*d*_6_) *δ* 154.85, 154.16, 153.11, 81.34, 80.72, 80.18, 78.80, 78.65, 74.99, 74.93, 39.39, 38.21, 37.62, 37.46, 36.15, 36.06, 27.81, 27.76, 27.73, 27.69. ^1^H NMR (400 MHz, at *T* = 90 °C DMSO-*d*_6_) *δ* 4.36 (d, *J* = 17.5 Hz, 1H), 3.99 (d, *J* = 17.9 Hz, 1H), 3.08–2.97 (m, 4H), 1.53–1.32 (m, 18H). HRMS (ESI) calculated for [M + Na]^+^ C_14_H_24_N_2_NaO_4_^+^ 307.1628, found 307.1633.

#### 4,5-Dibromo-1-methyl-2-(prop-2-yn-1-yl)-1,2-dihydropyridazine-3,6-dione (3)

To a stirred solution of di-*tert*-butyl 1-methyl-2-(prop-2-yn-1-yl)hydrazine-1,2-dicarboxylate (2) (0.90 g, 3.15 mmol) in acetic acid (30 mL) 2,3-dibromomaleic acid (0.98 g, 3.59 mmol) was added. The mixture was heated to 135 °C and stirred for 4 h. The mixture was concentrated under reduced pressure and the residue was taken up in acetone and concentrated onto silica gel. The crude was purified by flash column chromatography (cHex/EtOAc gradient from 1 : 0 to 1 : 1) to give the desired compound in 92% yield (0.93 g, 2.91 mmol). A small sample was recrystallised from a mixture of acetone and *n*-hexane and yielded single crystals of suitable quality for X-ray diffraction analysis (CCDC deposition number: 2171667). Colourless crystals: ^1^H NMR (300 MHz, CDCl_3_) *δ* 4.94 (d, *J* = 2.5 Hz, 2H), 3.78 (s, 3H), 2.43 (t, *J* = 2.5 Hz, 1H). 13C NMR (75 MHz, CDCl_3_) *δ* 152.87, 152.85, 136.93, 135.16, 75.68, 75.01, 37.17, 34.79. HRMS (ESI) calculated for [M + H]^+^ C_8_H_7_Br_2_N_2_O_2_^+^ 320.8869, found 320.8872.

#### *N*-(10-(4-((2-(2-azidoethoxy)ethyl)carbamoyl)-2-methylphenyl)-7-(dimethylamino)-5,5-dimethyldibenzo[*b*,*e*]silin-3(5*H*)-ylidene)-*N*-methylmethanaminium chloride (4)

To a stirred solution of *N*-(7-(dimethylamino)-10-(4-(((2,5-dioxopyrrolidin-1-yl)oxy)carbonyl)-2-methylphenyl)-5,5-dimethyldibenzo[*b*,*e*]silin-3(5*H*)-ylidene)-*N*-methylmethanaminium chloride (SiR-NHS) (0.029 g, 0.051 mmol) in MeCN (3 mL) was added 2-(2-azidoethoxy)ethan-1-amine (S8) (0.059 g, 0.457 mmol). The mixture was stirred at 21 °C for 2 h and protected from light. The mixture was concentrated under reduced pressure. The residue was taken up in CH_2_Cl_2_, the organic phase was washed with HCl (1 M), brine, dried over MgSO_4_, filtered and concentrated under reduced pressure onto silica gel. The crude was purified by flash column chromatography (CH_2_Cl_2_/MeOH gradient from 1 : 0 to 9 : 1) to give the desired product in quantitative yield (0.030 g, 0.0506 mmol). Purple solid: ^1^H NMR (300 MHz, DMSO-*d*_6_) *δ* 8.69 (t, *J* = 5.5 Hz, 1H), 7.93 (s, 1H), 7.87 (dd, *J* = 7.9, 1.7 Hz, 1H), 7.45 (d, *J* = 2.4 Hz, 2H), 7.27 (d, *J* = 7.9 Hz, 1H), 6.88 (d, *J* = 9.6 Hz, 2H), 6.82 (dd, *J* = 9.7, 2.5 Hz, 2H), 3.68–3.60 (m, 4H), 3.50 (t, *J* = 5.7 Hz, 2H), 3.46–3.40 (m, 2H), 3.31 (s, 12H), 2.02 (s, 3H), 0.61 (s, 3H), 0.59 (s, 3H). ^13^C NMR (101 MHz, DMSO-*d*_6_) *δ* 166.22, 165.77, 153.73, 147.16, 141.30, 139.78, 135.30, 134.60, 128.95, 128.90, 126.04, 124.61, 121.56, 114.57, 68.98, 68.68, 49.99, 40.50, 18.85, −1.04, −1.37. HRMS (ESI) calculated for [M-Cl]^+^ C_31_H_39_N_6_O_2_Si^+^ 555.2898, found 555.2878.

#### *N*-(6-azidohexyl)-5,7-difluoro-6-hydroxy-2-oxo-2*H*-chromene-3-carboxamide (6)

To a stirred, slightly turbid solution of pacific blue NHS ester (PB–NHS) (0.05 g, 0.15 mmol) in MeCN (10 mL) was added a solution of 6-azidohexan-1-amine (S9) (0.5 M in MeCN, 300 μL, 0.15 mmol, 0.21 g). The reaction mixture was left stirring at 21 °C for 24 h, after which it was concentrated onto silica gel. The crude was purified by flash column chromatography (EtOAc/(EtOAc/AcOH 99 : 1) gradient from 1 : 0 to 0 : 1) to give the desired compound in 90% yield (0.049 g, 0.135 mmol). Yellow powder: ^1^H NMR (300 MHz, CDCl_3_) *δ* 9.38 (br. s, 1H), 8.93 (t, *J* = 5.8 Hz, 1H), 8.80 (d, *J* = 1.4 Hz, 1H), 7.29–7.20 (m, 1H), 3.48 (q, *J* = 6.7 Hz, 2H), 3.26 (t, *J* = 6.8 Hz, 2H), 1.73–1.50 (m, 4H), 1.49–1.32 (m, 4H). 13C NMR (75 MHz, CDCl_3_) *δ* 162.16, 160.50, 151.07, 151.06, 148.19, 148.14, 148.10, 147.83, 147.78, 141.03, 141.00, 140.98, 140.95, 140.88, 140.85, 140.61, 140.45, 140.38, 140.21, 137.64, 116.09, 110.30, 110.26, 110.21, 109.99, 110.18, 109.99, 109.94, 51.44, 40.19, 29.20, 28.81, 26.60, 26.45. HRMS (ESI) calculated for [M + H]^+^ C_16_H_17_O_4_N_4_F_2_ 367.1212, found 367.1212.

#### *N*-(2-(2-(2-(2-azidoethoxy)ethoxy)ethoxy)ethyl)-5-((3a*S*,4*S*,6a*R*)-2-oxohexahydro-1H-thieno[3,4-*d*]imidazole-4-yl)pentanamide (7)

To a stirred solution of biotin-NHS ester (biotin-NHS) (0.1 g, 0.29 mmol) in DMF (10 mL) was added 2-(2-(2-(2-azidoethoxy)ethoxy)ethoxy)ethan-1-amine (S10) (0.146 g, 0.669 mmol) the mixture was stirred under an argon atmosphere for 16 h. The volatiles were removed under reduced pressure. The colourless residue was taken up in CH_2_Cl_2_ and was concentrated onto silica gel. The crude was purified by flash column chromatography (CH_2_Cl_2_/MeOH 9 : 1 gradient from 1 : 0 to 9 : 1) to give the desired compound in quantitative yield (0.139 g, 0.313 mmol). Colourless solid: ^1^H NMR (300 MHz, DMSO-*d*_6_) *δ* 7.82 (t, *J* = 5.6 Hz, 1H), 6.39 (d, *J* = 20.1 Hz, 2H), 4.38–4.23 (m, 1H), 4.19–4.05 (m, 1H), 3.68–3.45 (m, 10H), 3.45–3.29 (m, 5H), 3.18 (q, *J* = 5.9 Hz, 2H), 3.13–3.01 (m, 1H), 2.82 (dd, *J* = 12.4, 5.0 Hz, 1H), 2.57 (d, *J* = 12.3 Hz, 1H), 2.06 (t, *J* = 7.3 Hz, 2H), 1.69–1.38 (m, 4H), 1.36–1.20 (m, 2H). ^13^C NMR (75 MHz, DMSO-*d*_6_) *δ* 172.12, 162.73, 69.81, 69.78, 69.71, 69.57, 69.27, 69.18, 61.05, 59.21, 55.43, 50.00, 39.86, 38.45, 35.10, 28.20, 28.04, 25.26. HRMS (ESI) calculated for [M + H]^+^ C_18_H_33_O_5_N_6_S 445.2228, found 445.2221.

#### *N*-(10-(4-((2-(2-(4-((4,5-dibromo-2-methyl-3,6-dioxo-3,6-dihydropyridazin-1(2*H*)-yl)methyl)-1*H*-1,2,3-triazol-1-yl)ethoxy)ethyl)carbamoyl)-2-methylphenyl)-7-(dimethylamino)-5,5-dimethyldibenzo[*b*,*e*]silin-3(5*H*)-ylidene)-*N*-methylmethanaminium chloride (8)

To a stirred solution of *N*-(10-(4-((2-(2-azidoethoxy)ethyl)carbamoyl)-2-methylphenyl)-7-(dimethylamino)-5,5-dimethyldibenzo[*b*,*e*]silin-3(5*H*)-ylidene)-*N*-methylmethanaminium chloride (4) (0.034 g, 0.058 mmol) and 4,5-dibromo-1-methyl-2-(prop-2-yn-1-yl)-1,2-dihydropyridazine-3,6-dione (3) (0.021 g, 0.066 mmol) in *t*-BuOH (3 mL) and water (3 mL) was added sodium ascorbate (0.005 g, 0.023 mmol) and CuSO_4_·5H_2_O (0.001 g, 0.005 mmol). The mixture was stirred at 21 °C for 3 h. The mixture was concentrated under reduced pressure onto silica gel. The crude was purified by flash column chromatography (CH_2_Cl_2_/MeOH gradient from 1 : 0 to 9 : 1) to give the desired compound in 95% yield (0.05 g, 0.05 mmol). Blue solid: ^1^H NMR (300 MHz, DMSO-*d*_6_) *δ* 8.61 (t, *J* = 5.4 Hz, 1H), 8.17 (s, 1H), 7.92 (s, 1H), 7.85 (d, *J* = 7.9 Hz, 1H), 7.45 (d, *J* = 2.6 Hz, 2H), 7.27 (d, *J* = 7.9 Hz, 1H), 6.88 (d, *J* = 9.7 Hz, 2H), 6.81 (dd, *J* = 9.7, 2.6 Hz, 2H), 5.33 (s, 2H), 4.55 (t, *J* = 5.1 Hz, 2H), 3.85 (t, *J* = 5.1 Hz, 2H), 3.63 (s, 3H), 3.56 (t, *J* = 5.7 Hz, 2H), 3.42 (q, *J* = 5.5 Hz, 2H), 3.31 (s, 12H), 2.02 (s, 3H), 0.61 (s, 3H), 0.59 (s, 3H). 13C NMR (101 MHz, DMSO-*d*_6_) *δ* 166.24, 165.70, 153.72, 152.42, 152.24, 147.16, 141.33, 140.81, 139.80, 135.68, 135.37, 134.70, 134.52, 128.97, 128.92, 126.04, 124.56, 124.47, 121.55, 114.55, 68.66, 68.29, 54.90, 49.51, 42.45, 40.50, 34.85, 18.87, −1.04, −1.37. HRMS (ESI) calculated for [M–Cl]^+^ C_39_H_45_Br_2_N_8_O_4_Si^+^ 875.1694, found 875.1685.

#### *N*-(3-(4-((4,5-dibromo-2-methyl-3,6-dioxo-3,6-dihydropyridazin-1(2*H*)-yl)methyl)-1*H*-1,2,3-triazol-1-yl)propyl)-3-(5,5-difluoro-7,9-dimethyl-5*H*-5λ,^4^ 6λ ref. 4-dipyrrolo[1,2-*c*:2′,1′-*f*][1,3,2]diazaborinin-3-yl)propenamide (9)

4,5-dibromo-1-methyl-2-(prop-2-yn-1-yl)-1,2-dihydropyridazine-3,6-dione (3) (0.016 g, 0.05 mmol) and *N*-(3-azidopropyl)-3-(5,5-difluoro-7,9-dimethyl-5*H*-5_λ_^4^, 6_λ_^4^-dipyrrolo[1,2-c:2′,1′-f][1,3,2]diazaborinin-3-yl)propenamide (5) (0.018 g, 0.047 mmol) was dissolved in *t*-BuOH (2 mL) and H_2_O (1 mL). CuSO_4_·5H_2_O (1 M in H_2_0, 2 μL, 0.002 mmol) and sodium ascorbate (0.003 g, 0.013 mmol) was added to this solution. The mixture was stirred at 23 °C for 16 h, under an argon atmosphere and protected from light. The reaction mixture was diluted with MeCN and concentrated onto silica gel. The crude was purified by flash column chromatography (cHex/(EtOAc/MeOH/AcOH 85 : 10 : 5) gradient from 1 : 0 to 0 : 1) to give the desired compound in quantitative yield (0.034 g, 0.049 mmol). Dark orange powder: ^1^H NMR (300 MHz, DMSO-*d*_6_) *δ* 8.16 (s, 1H), 8.02 (t, *J* = 5.6 Hz, 1H), 7.69 (s, 1H), 7.08 (d, *J* = 4.0 Hz, 1H), 6.35 (d, *J* = 4.0 Hz, 1H), 6.30 (s, 1H), 5.37 (s, 2H), 4.34 (t, *J* = 7.0 Hz, 2H), 3.64 (s, 3H), 3.07 (dd, *J* = 9.6, 6.7 Hz, 4H), 2.47 (s, 9H), 2.26 (s, 3H), 1.94 (p, *J* = 7.0 Hz, 3H). ^13^C NMR (101 MHz, DMSO-*d*_6_) *δ* 170.92, 159.16, 152.38, 152.25, 140.89, 135.70, 134.72, 132.95, 128.89, 128.19, 125.35, 125.30, 124.14, 120.26, 116.56, 47.35, 42.46, 35.74, 34.81, 33.76, 29.81, 23.95, 14.50, 10.98, 1.14. ^19^F NMR (376 MHz, DMSO-*d*_6_) *δ*-143.18 (dd, *J* = 66.5, 33.0 Hz). ^11^B NMR (96 MHz, DMSO-*d*_6_) *δ* 0.74 (t, *J* = 32.8 Hz). HRMS (ESI) calculated for [M + Na]^+^ C_25_H_27_O_3_N_8_ BBr_2_F_2_Na 717.0526, found 717.0523.

#### *N*-(6-(4-((4,5-dibromo-2-methyl-3,6-dioxo-3,6-dihydropyridazin-1(2*H*)-yl)methyl)-1*H*-1,2,3-triazol-1-yl)hexyl)-5,7-difluoro-6-hydroxy-2-oxo-2*H*-chromene-3-carboxamide (10)

To a stirred suspension of 4,5-dibromo-1-methyl-2-(prop-2-yn-1-yl)-1,2-dihydropyridazine-3,6-dione (3) (0.051 g, 0.157 mmol) and *N*-(6-azidohexyl)-5,7-difluoro-6-hydroxy-2-oxo-2*H*-chromene-3-carboxamide (6) (0.056 g, 0.154 mmol) and sodium ascorbate (0.004 g, 0.02 mmol) in *tert*-butanol (2 mL) and water (2 mL) was added a solution of CuSO_4_·5H_2_O (1 M, 10 μL, 0.003 g, 10 μmol). The mixture was placed under an argon atmosphere and was stirred for 48 h. The suspension was filtered through a pad of Celite and the filter cake was washed with water. The filtrate was discarded. The filter cake was washed with MeCN. The resulting filtrate was then concentrated onto silica gel. The crude was purified by flash column chromatography (EtOAc/(EtOAc/acetic acid 99 : 1) gradient from 1 : 0 to 0 : 1) to give the desired compound in 67% yield (0.071 g, 0.105 mmol). Yellow powder: ^1^H NMR (300 MHz, DMSO-*d*_6_) *δ* 12.02 (s, 1H), 8.76 (s, 1H), 8.56 (t, *J* = 5.8 Hz, 1H), 8.16 (s, 1H), 7.74 (d, *J* = 9.6 Hz, 1H), 5.36 (s, 2H), 4.33 (t, *J* = 7.1 Hz, 2H), 3.63 (s, 3H), 3.31–3.24 (m, 2H), 1.80 (p, *J* = 7.1 Hz, 2H), 1.49 (p, *J* = 6.6 Hz, 2H), 1.32–1.19 (m, 4H). ^13^C NMR (101 MHz, DMSO-*d*_6_) *δ* 160.97, 159.66, 152.38, 152.24, 147.00, 140.92, 140.62, 140.59, 140.54, 135.68, 134.73, 123.91, 115.99, 110.53, 110.32, 109.26, 109.18, 49.38, 42.51, 34.80, 29.47, 28.74, 25.68, 25.44. ^19^F NMR (282 MHz, DMSO-*d*_6_) *δ*-135.17, −154.28. HRMS (ESI) calculated for [M + Na]^+^ C_24_H_22_O_6_N_6_Br_2_F_2_Na 708.9828, found 708.9854.

#### *N*-(2-(2-(2-(2-(4-((4,5-dibromo-2-methyl-3,6-dioxo-3,6-dihydropyridazin-1(2*H*)-yl)methyl)-1*H*-1,2,3-triazol-1-yl)ethoxy)ethoxy)ethoxy)ethyl)-5-((3a*S*,4*S*,6a*R*)-2-oxohexahydro-1*H*-thieno[3,4-*d*]imidazole-4-yl)pentanamide (11)

*N*-(2-(2-(2-(2-azidoethoxy)ethoxy)ethoxy)ethyl)-5-((3a*S*,4*S*,6a*R*)-2-oxohexahydro-1*H*-thieno[3,4-*d*]imidazole-4-yl)pentanamide (7) (0.112 g, 0.252 mmol) was dissolved in a mixture of *t*-BuOH (3 mL) and water (3 mL), using some gentle heating to obtain a clear solution. To this was added 4,5-dibromo-1-methyl-2-(prop-2-yn-1-yl)-1,2-dihydropyridazine-3,6-dione (3) (0.089 g, 0.278 mmol), sodium ascorbate (0.005 g, 0.027 mmol) and CuSO_4_·5H_2_O (0.002 g, 0.007 mmol). The mixture was stirred at 21 °C for 6 h. The mixture was concentrated under reduced pressure. The slight greenish residue was taken up in a mixture of CH_2_Cl_2_ and MeOH. This mixture was then concentrated onto silica gel. The crude was purified by flash column chromatography (CH_2_Cl_2_/MeOH gradient from 1 : 0 to 9 : 1) to give the desired compound in 91% yield (0.175 g, 0.228 mmol). Pale yellow powder: ^1^H NMR (400 MHz, DMSO-*d*_6_) *δ* 8.12 (s, 1H), 7.80 (t, *J* = 5.6 Hz, 1H), 6.38 (d, *J* = 23.7 Hz, 2H), 5.37 (s, 2H), 4.51 (t, *J* = 5.2 Hz, 2H), 4.30 (dd, *J* = 7.7, 5.0 Hz, 1H), 4.16–4.08 (m, 1H), 3.80 (t, *J* = 5.3 Hz, 2H), 3.65 (s, 3H), 3.52–3.43 (m, 8H), 3.38 (t, *J* = 6.0 Hz, 2H), 3.17 (q, *J* = 5.9 Hz, 2H), 3.14–3.04 (m, 1H), 2.81 (dd, *J* = 12.4, 5.1 Hz, 1H), 2.57 (d, *J* = 12.5 Hz, 1H), 2.06 (t, *J* = 7.4 Hz, 2H), 1.66–1.54 (m, 1H), 1.56–1.38 (m, 3H), 1.35–1.22 (m, 2H). ^13^C NMR (101 MHz, DMSO-*d*_6_) *δ* 172.09, 162.67, 152.39, 152.24, 140.80, 135.69, 134.73, 124.46, 69.67, 69.61, 69.52, 69.14, 68.56, 61.01, 59.17, 55.39, 54.89, 49.50, 42.41, 39.83, 38.41, 35.08, 34.81, 28.17, 28.02, 25.23. HRMS (ESI) calculated for [M + H]^+^ C_26_H_39_O_7_N_8_Br_2_S 765.1024, found 765.1024.

### Biology

#### General remarks

Antibodies used were purchased from Invitrogen (anti GAPDH, MA5-15738), Abcam (anti β-actin, ab6276; anti Na^+^/K^+^-ATPase, ab7671), Alomone Labs (anti Ca_v_1.2, ACC-003) or custom-generated by Pineda antibody services (anti Na_v_1.5 and anti TRPM4). All experiments involving animals (mouse hearts) were performed according to the Swiss Federal Animal Protection Law and were approved by the Cantonal Veterinary Administration, Bern, Switzerland. This investigation conforms to the Guide for the Care and Use of Laboratory Animals, published by the US National Institutes of Health (NIH publication no. 85-23, revised 1996).

Buffers used: PBS (Sigma-Aldrich (P5368) dissolved in 1 L Milli-Q water), BBS (Na_2_B_4_O_7_·10H_2_O 25 mM, NaCl 25 mM, EDTA 0.5 mM, DMSO 2% in Milli-Q water, pH adjusted with aq. HCl to 8.01), SDS-Running Buffer (25 mM Tris, 190 mM glycine, 0.1% SDS), Transfer Buffer (25 mM Tris, 190 mM glycine, 20% MeOH, 0.1% SDS), TBST (20 mM Tris, pH 7.5, 150 mM NaCl, 0.1 Tween 20), 4*x* loading buffer (0.2 M Tris pH 6.8, 40% glycerol, 20% 2-mercaptoethanol, 8% SDS, 0.002% bromophenol blue in deionised water).

### Antibody conjugation through re-bridging

The buffer, in which the antibody was obtained, was exchanged to Borate Buffered Saline (BBS, pH = 8.0) using spin filter columns (Amicon® Ultra 2 mL, 30′000 Da molecular weight cut-off, 3 × 15 minutes @ 4000 g). The residue was transferred into a PCR reaction tube. The re-bridging compound 8–11 (20 mM in DMSO) was added in 20-fold excess with respect to the antibody. This mixture was incubated for 1 h at 4 °C. Then TCEP (10 mM in water) was added in 10-fold excess with respect to the antibody and the reaction was kept at 4 °C overnight. The reaction residue was transferred into a spin filter column (Amicon® Ultra 2 mL, 30′000 Da molecular weight cut-off) and washed with phosphate buffered saline (PBS, pH = 7.4) (6 × 15 minutes @ 4000 g). After washing, the modified antibody was diluted to the original concentration with PBS, aliquoted and stored at −20 °C.

### Stochastic antibody fluorescent labelling

The buffer, in which the antibody was obtained, was exchanged to Borate Buffered Saline (BBS, pH = 8.0) using spin filter columns (Amicon® Ultra 2 mL, 30′000 Da molecular weight cut-off, 3 × 15 minutes @ 4000 g). The residue was transferred into a PCR reaction tube. To this a solution of SiR-NHS (10 mM in DMSO) was added in 20-fold excess with respect to the antibody. This mixture was incubated at 4 °C overnight. The reaction residue was transferred into a spin filter column (Amicon® Ultra 2 mL, 30′000 Da molecular weight cut-off) and washed with phosphate buffered saline (PBS, pH = 7.4, 5 × 15 minutes @ 4000 g and 1 × 15 minutes @ 4000 g). After washing, the modified antibody was diluted to the original concentration with PBS, aliquoted and stored at −20 °C.

### SDS-PAGE

Polyacrylamide gels were cast from 30% acrylamide solution (Carl Roth, A124.1). For a typical SDS-PAGE experiment the modified antibody in PBS was diluted with 4*x* loading buffer to obtain a loading of 200 ng per lane. In this dilution the mixture was incubated at 92 °C for 30 minutes. A 10% 1 mm polyacrylamide gel was run at 90 V for 15 minutes followed by 120 V up to a total run time of 2.5 h in SDS running buffer. Visualisation was achieved with a Typhoon FLA 9500 fluorescence imager. After the fluorescence analysis, the gel was stained with Coomassie blue staining for 1 h followed by washing with water overnight.

### Size exclusion chromatography (SEC)

SEC was conducted on an Agilent 1260 infinity II HPLC with 150 mM phosphate buffer + 0.01% NaN_3_, pH = 7 as eluent, using an Agilent AdvanceBio SEC 2.7 μm 300 Å fully porous column (7.8 × 300 mm) and an Agilent AdvanceBio SEC 2.7 μm 300 Å fully porous guard column (7.8 × 50 mm). Proteins were detected by fluorescence and absorption at 215 nm and 280 nm (Fig. S2, ESI†).

### Western blotting

In a typical western blot experiment, cardiomyocytes were isolated from mouse heart according to Ozhathil *et al.*^35^ and lysed. The lysate was diluted with 4*x* loading buffer to obtain a loading of 100 μg per lane. In this dilution the mixture was incubated at 37 °C for 30 minutes. A 10% 1 mm polyacrylamide gel was run at 90 V for 15 minutes followed by 120 V up to a total run time of 2.5 h in SDS running buffer. The gel was removed and incubated in transfer buffer for 15 minutes. The gel was sandwiched together with a 0.4 μM nitrocellulose membrane and was wet-blotted at 90 V for 90 minutes in 0 °C transfer buffer. The membrane was then blocked with 3% BSA in TBST for 1 h after which it was rinsed 5*x* with TBST. Staining of the targets was achieved by incubating the membrane in a solution of the corresponding antibody diluted in TBST + 3% BSA, using a concentration for western blot application recommended by the manufacturer. Visualisation was achieved with a fluorescence imager (GE Healthcare, Typhoon FLA 9500). The biotin-modified antibody was visualised by co-incubating the membrane with streptavidin-Cy5 (Invitrogen™, SA1011) solution.

### Immunofluorescence

Adult mouse heart cardiomyocytes were isolated as described previously^35^ and plated onto uncoated PCA microscopy chamber slides (Sartstedt, 94.6140.802). The cells were washed twice with PBS 1*x* and then fixed with 10% PFA for 15 minutes. The cells were washed twice with PBS 1*x*, blocked with blocking buffer (PBS 1*x* + 1% BSA + 0.5% TritonX-100 + 10% Goat Serum) for 30 minutes and subsequently incubated overnight at 4 °C in incubation buffer (PBS 1*x* + 1% BSA + 0.5% Triton X-100 + 3% Goat Serum). The incubation buffer contained the corresponding antibody in a concentration for immunofluorescence applications, according to the manufacturer's recommendation. The slides were washed 3*x* with PBS 1*x*. For indirect immunofluorescence or biotin detection, secondary antibodies (Abcam, ab96879 (goat anti mouse), ab98462 (goat anti rabbit)) or streptavidin-Cy5 was applied, respectively, in the recommended concentration in incubation buffer for 1 h at 22 °C. For direct immunofluorescence using a fluorescent primary antibody, the cells were kept in PBS 1*x* for 1 h at 22 °C. Subsequently, the slides were washed 3*x* with PBS 1*x*, applying DAPI (Sigma-Aldrich, D9542) stain in the second wash. The cover slip was mounted with Fluor Save (Calbiochem, 345789). The microscope used was a Zeiss LSM710 with EC Plan-NeoFluor 40x objective.

## Author contributions

P. Grossenbacher, B. Stieger and M. Lochner conceived the project. P. Grossenbacher, M. C. Essers, S. A. Singer, B. Stieger, J.-S. Rougier and M. Lochner designed the experiments. P. Grossenbacher, M. C. Essers, J. Moser, S. A. Singer and S. Häusler performed the experiments and data analysis. P. Grossenbacher and M. Lochner wrote the manuscript. All authors edited and approved the manuscript.

## Conflicts of interest

The authors declare no conflict of interest.

## Supplementary Material

RA-012-D2RA05580E-s001

RA-012-D2RA05580E-s002

RA-012-D2RA05580E-s003

## References

[cit1] Khongorzul P., Ling C. J., Khan F. U., Ihsan A. U., Zhang J. (2020). Mol. Cancer Res..

[cit2] Walsh S. J., Bargh J. D., Dannheim F. M., Hanby A. R., Seki H., Counsell A. J., Ou X., Fowler E., Ashman N., Takada Y., Isidro-Llobet A., Parker J. S., Carroll J. S., Spring D. R. (2021). Chem. Soc. Rev..

[cit3] Hermanson G. T. , Bioconjugate Techniques, Academic Press, 3rd edn, 2013

[cit4] Chudasama V., Maruani A., Caddick S. (2016). Nat. Chem..

[cit5] Junutula J. R., Raab H., Clark S., Bhakta S., Leipold D. D., Weir S., Chen Y., Simpson M., Tsai S. P., Dennis M. S., Lu Y., Meng Y. G., Ng C., Yang J., Lee C. C., Duenas E., Gorrell J., Katta V., Kim A., McDorman K., Flagella K., Venook R., Ross S., Spencer S. D., Lee Wong W., Lowman H. B., Vandlen R., Sliwkowski M. X., Scheller R. H., Polakis P., Mallet W. (2008). Nat. Biotechnol..

[cit6] Shen B.-Q., Xu K., Liu L., Raab H., Bhakta S., Kenrick M., Parsons-Reponte K. L., Tien J., Yu S.-F., Mai E., Li D., Tibbitts J., Baudys J., Saad O. M., Scales S. J., McDonald P. J., Hass P. E., Eigenbrot C., Nguyen T., Solis W. A., Fuji R. N., Flagella K. M., Patel D., Spencer S. D., Khawli L. A., Ebens A., Wong W. L., Vandlen R., Kaur S., Sliwkowski M. X., Scheller R. H., Polakis P., Junutula J. R. (2012). Nat. Biotechnol..

[cit7] Liberatore F. A., Comeau R. D., McKearin J. M., Pearson D. A., Belonga B. Q., Brocchini S. J., Kath J., Phillips T., Oswell K., Lawton R. G. (1990). Bioconjugate Chem..

[cit8] Del Rosario R. B., Wahl R. L., Brocchini S. J., Lawton R. G., Smith R. H. (1990). Bioconjugate Chem..

[cit9] Wei D., Mao Y., Xu Z., Chen J., Li J., Jiang B., Chen H. (2021). Bioorg. Med. Chem..

[cit10] Koniev O., Dovgan I., Renoux B., Ehkirch A., Eberova J., Cianférani S., Kolodych S., Papot S., Wagner A. (2018). Med. Chem. Commun..

[cit11] Shi B., Wu M., Li Z., Xie Z., Wei X., Fan J., Xu Y., Ding D., Akash S. H., Chen S., Cao S. (2019). Cancer Med..

[cit12] Hu Q.-Y. and Imase H., Methods for making conjugates from disulfide-containing proteins, 2014, p. WO2014083505

[cit13] Schumacher F. F., Nunes J. P. M., Maruani A., Chudasama V., Smith M. E. B., Chester K. A., Baker J. R., Caddick S. (2014). Org. Biomol. Chem..

[cit14] Nunes J. P. M., Morais M., Vassileva V., Robinson E., Rajkumar V. S., Smith M. E. B., Pedley R. B., Caddick S., Baker J. R., Chudasama V. (2015). Chem. Commun..

[cit15] Morais M., Nunes J. P. M., Karu K., Forte N., Benni I., Smith M. E. B., Caddick S., Chudasama V., Baker J. R. (2017). Org. Biomol. Chem..

[cit16] Maruani A., Smith M. E. B., Miranda E., Chester K. A., Chudasama V., Caddick S. (2015). Nat. Commun..

[cit17] Maruani A., Savoie H., Bryden F., Caddick S., Boyle R., Chudasama V. (2015). Chem. Commun..

[cit18] Lee M. T. W., Maruani A., Richards D. A., Baker J. R., Caddick S., Chudasama V. (2017). Chem. Sci..

[cit19] Robinson E., Nunes J. P. M., Vassileva V., Maruani A., Nogueira J. C. F., Smith M. E. B., Pedley R. B., Caddick S., Baker J. R., Chudasama V. (2017). RSC Adv..

[cit20] Bahou C., Richards D. A., Maruani A., Love E. A., Javaid F., Caddick S., Baker J. R., Chudasama V. (2018). Org. Biomol. Chem..

[cit21] Shao S., Tsai M.-H., Lu J., Yu T., Jin J., Xiao D., Jiang H., Han M., Wang M., Wang J. (2018). Bioorg. Med. Chem. Lett..

[cit22] Javaid F., Pilotti C., Camilli C., Kallenberg D., Bahou C., Blackburn J., Baker J. R., Greenwood J., Moss S. E., Chudasama V. (2021). RSC Chem. Biol..

[cit23] Walsh S. J., Omarjee S., Galloway W. R. J. D., Kwan T. T. L., Sore H. F., Parker J. S., Hyvönen M., Carroll J. S., Spring D. R. (2019). Chem. Sci..

[cit24] Walsh S. J., Omarjee S., Dannheim F. M., Couturier D.-L., Bexheti D., Mendil L., Cronshaw G., Fewster T., Gregg C., Brodie C., Miller J. L., Houghton R., Carroll J. S., Spring D. R. (2022). Chem. Commun..

[cit25] Hanby A. R., Walsh S. J., Counsell A. J., Ashman N., Mortensen K. T., Carroll J. S., Spring D. R. (2022). Chem. Commun..

[cit26] Bahou C., Love E. A., Leonard S., Spears R. J., Maruani A., Armour K., Baker J. R., Chudasama V. (2019). Bioconjugate Chem..

[cit27] Lee M. T. W., Maruani A., Baker J. R., Caddick S., Chudasama V. (2016). Chem. Sci..

[cit28] Koide Y., Urano Y., Hanaoka K., Terai T., Nagano T. (2011). ACS Chem. Biol..

[cit29] Nicholls C., Li H., Liu J.-P. (2012). Clin. Exp. Pharmacol. Physiol..

[cit30] Sirover M. A. (1999). Biochim. Biophys. Acta, Protein Struct. Mol. Enzymol..

[cit31] Sirover M. A. (2011). Biochim. Biophys. Acta, Gen. Subj..

[cit32] The Universal Protein Resource (UniProt), https://www.uniprot.org/uniprot/P16858, accessed April 2022

[cit33] Liu H., May K. (2012). mAbs.

[cit34] Vidarsson G., Dekkers G., Rispens T. (2014). Front. Immunol..

[cit35] Ozhathil L. C., Rougier J.-S., Arullampalam P., Essers M. C., Ross-Kaschitza D., Abriel H. (2021). Int. J. Mol. Sci..

