# Supplementary material for: Bioorthogonal site-selective conjugation of fluorescent dyes to antibodies: method and potential applications
Source: RSC Adv. 2022 Oct 5;12(44):28306–17. doi: 10.1039/d2ra05580e (PMC9533196; doi:10.1039/d2ra05580e)

Electronic Supplementary Material (ESI) for RSC Advances.
This journal is © The Royal Society of Chemistry 2022

Figure 1B in main Text

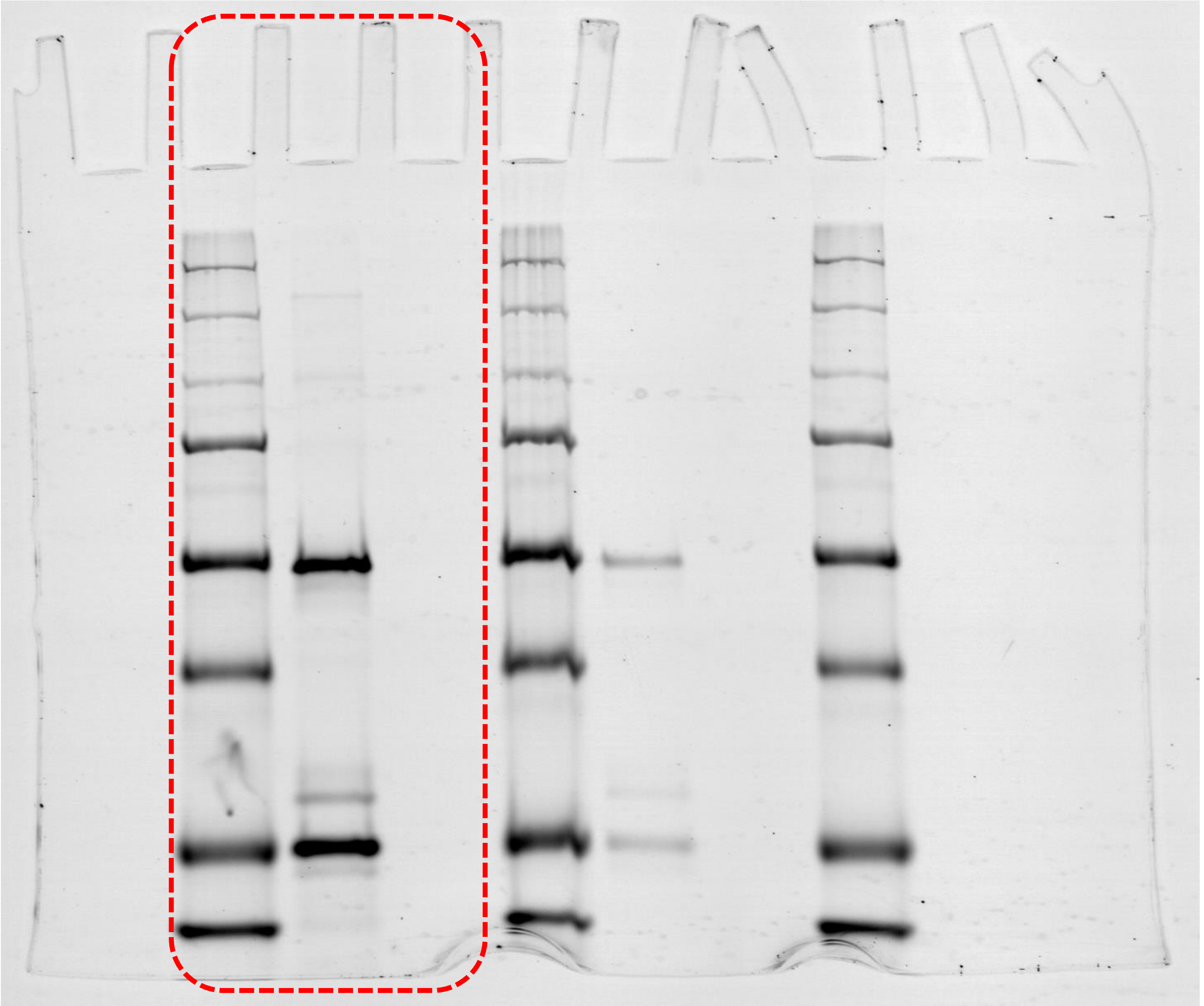

Figure 1C in main Text

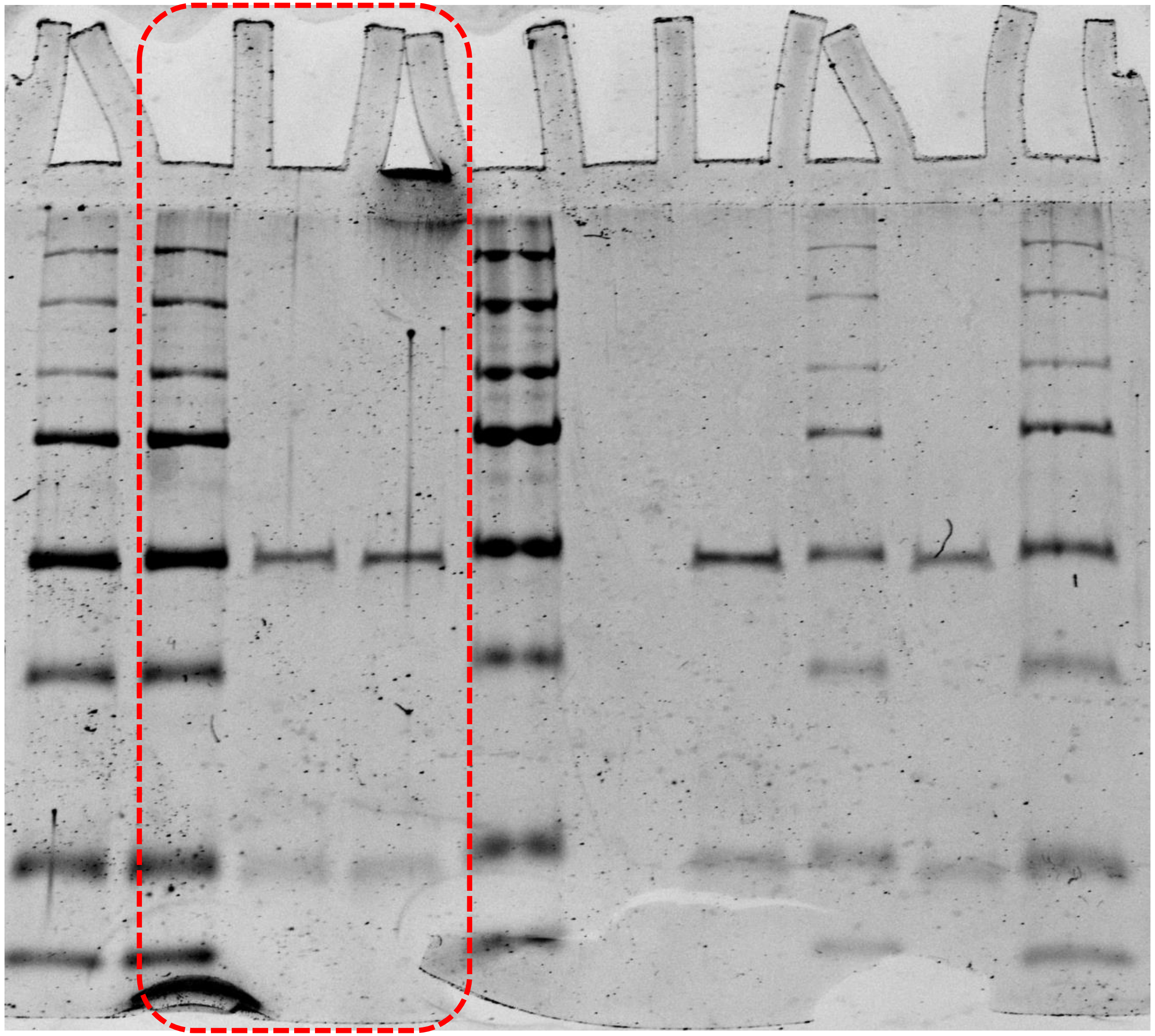

Figure 2A in main Text

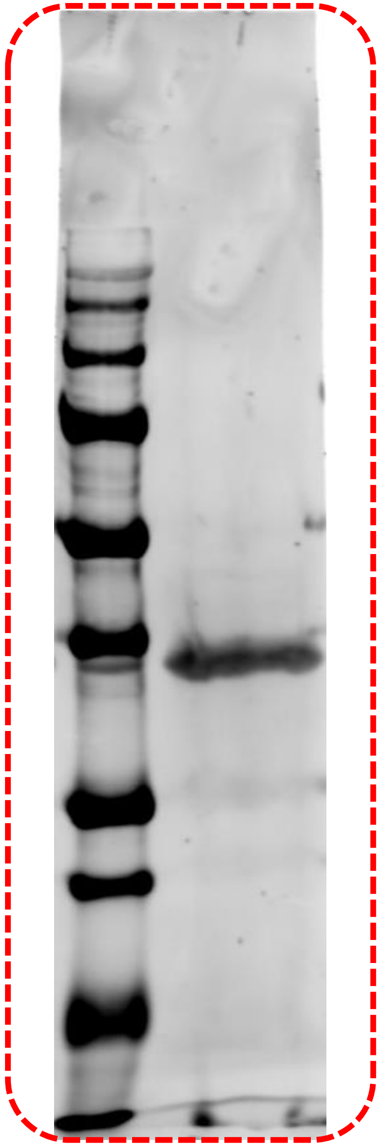

Figure 2B in main Text

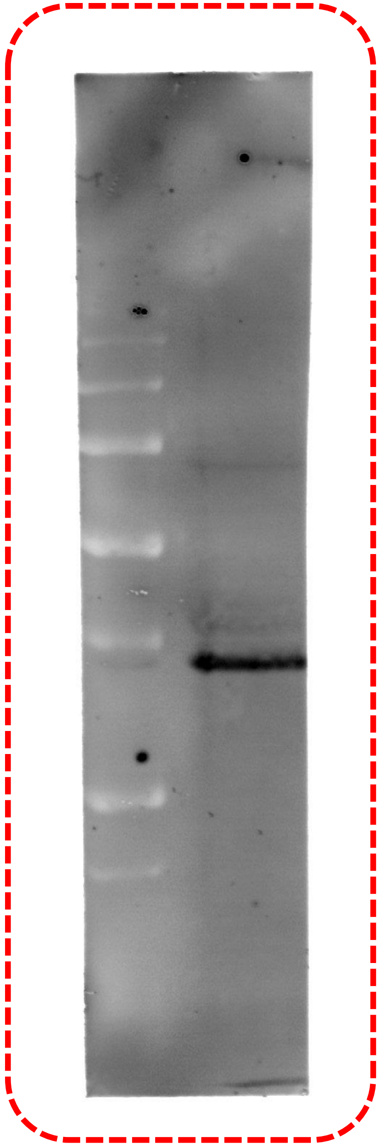

Figure 2C in main Text

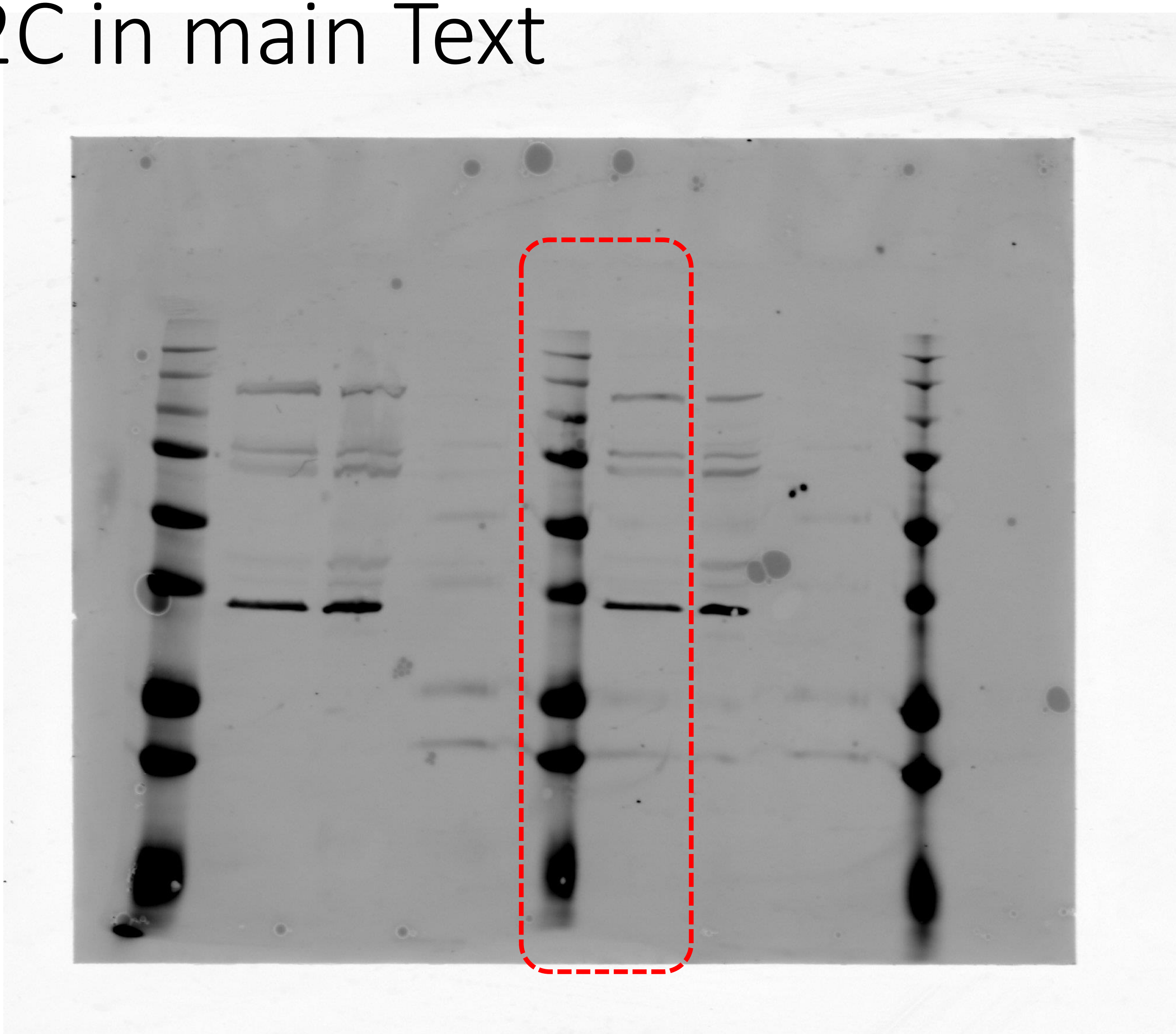

Figure S1 in ESI

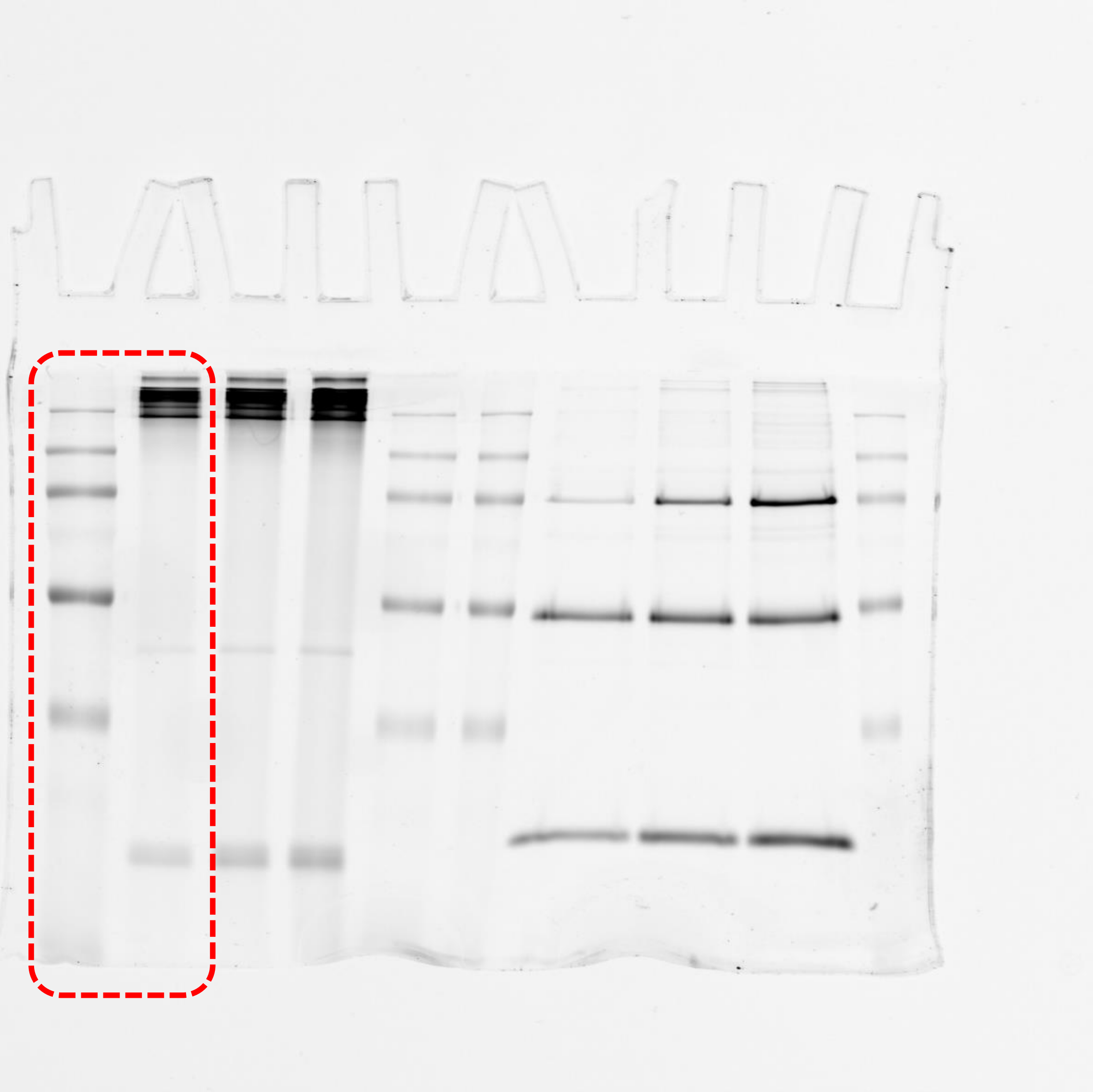

Figure S3A in ESI

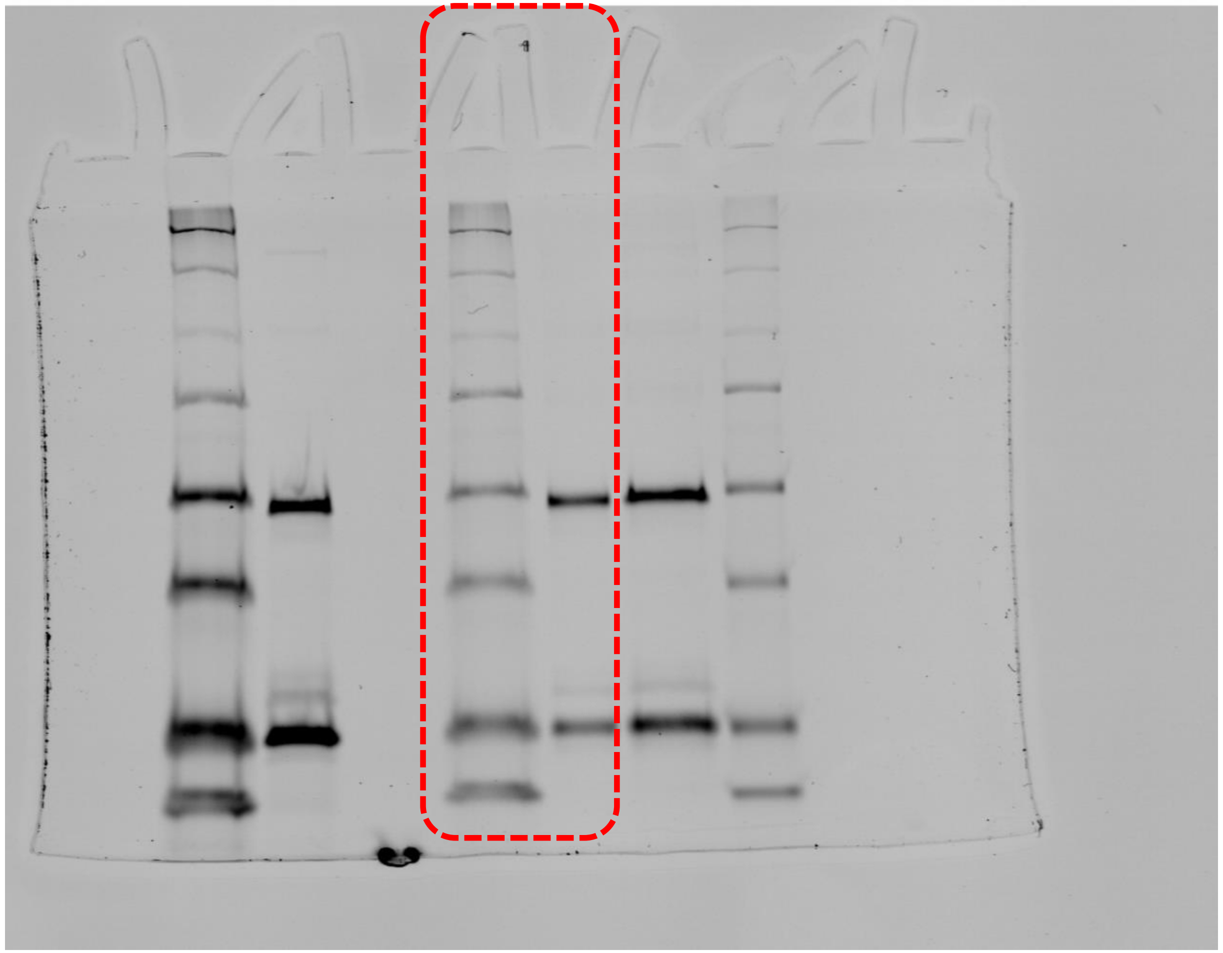

Figure S3B in ESI

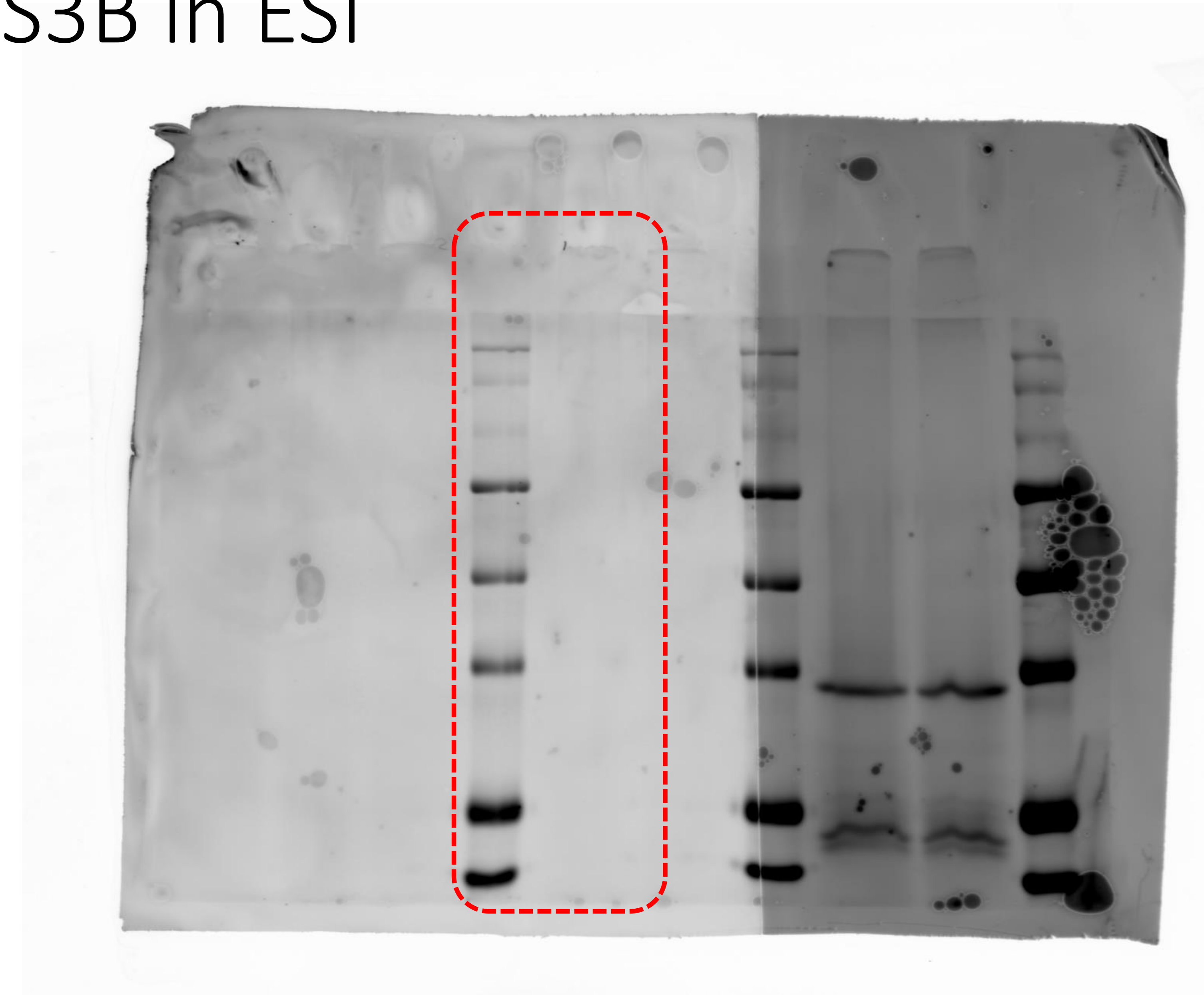

Figure S4A in ESI

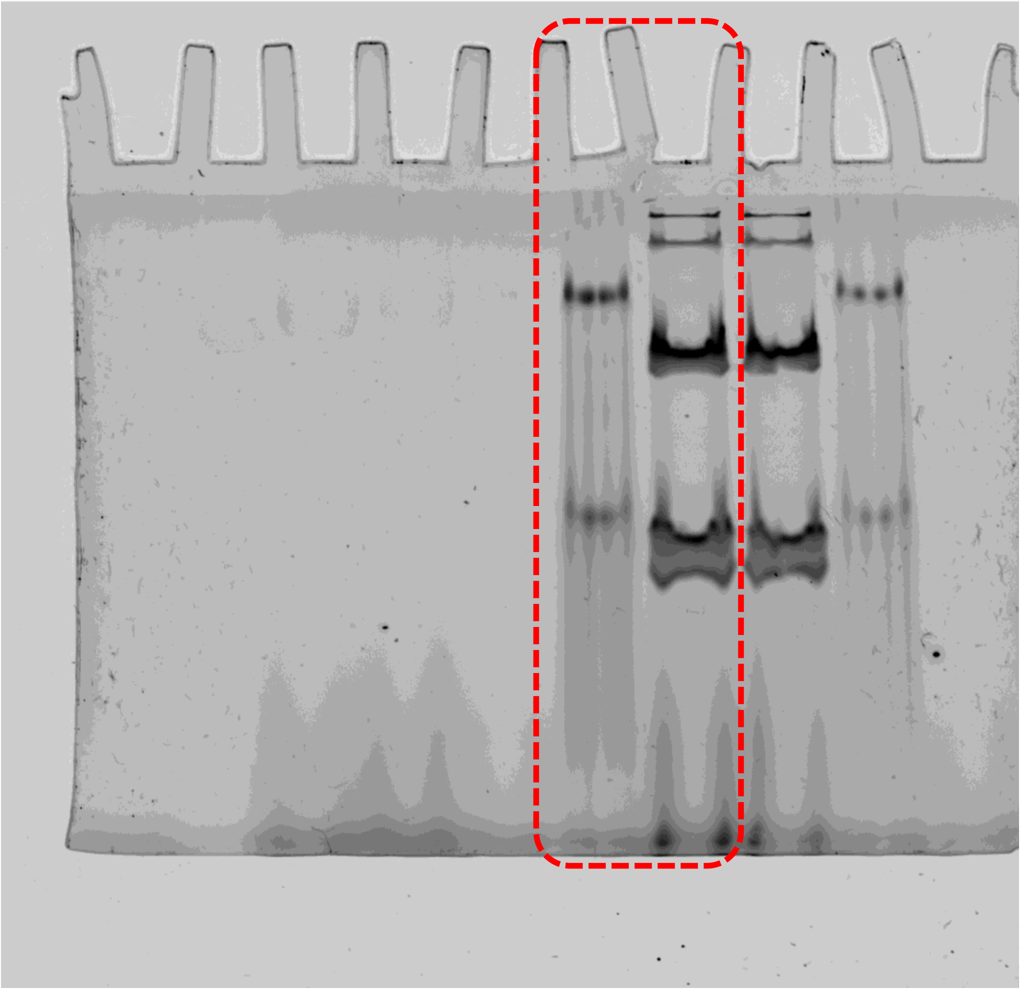

Figure S4B in ESI

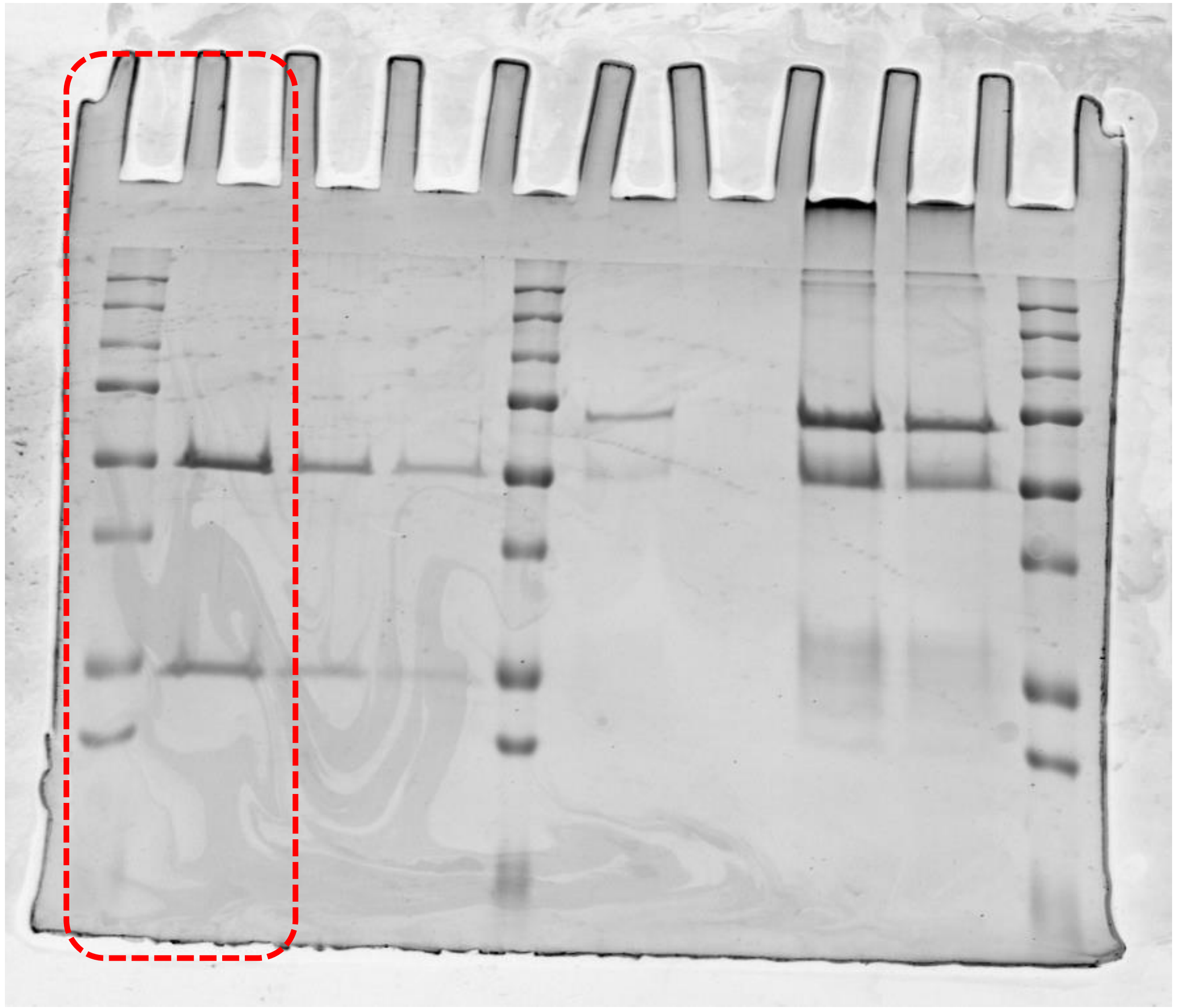

Figure S4C in ESI

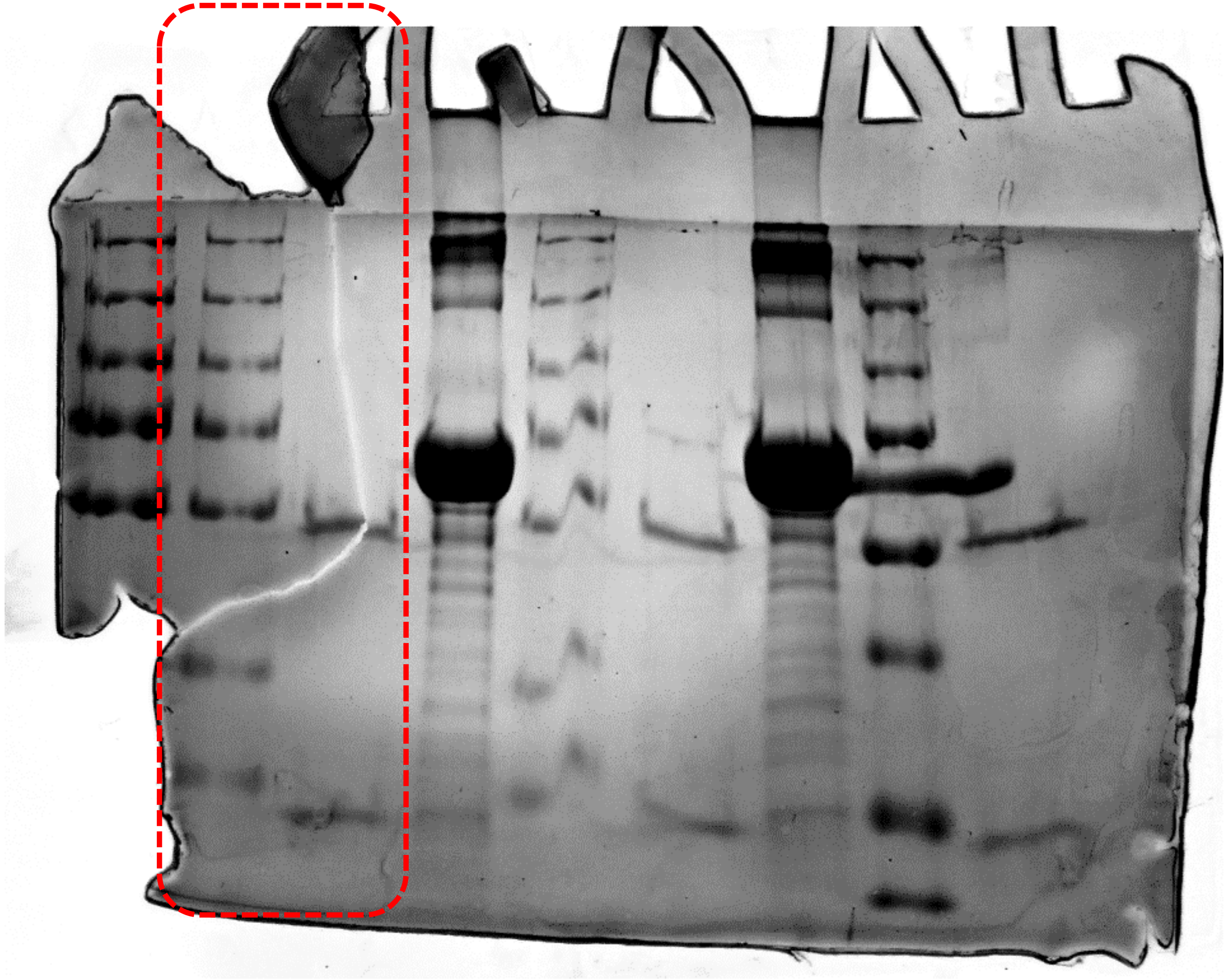

Figure S4D Cy5 channel in ESI

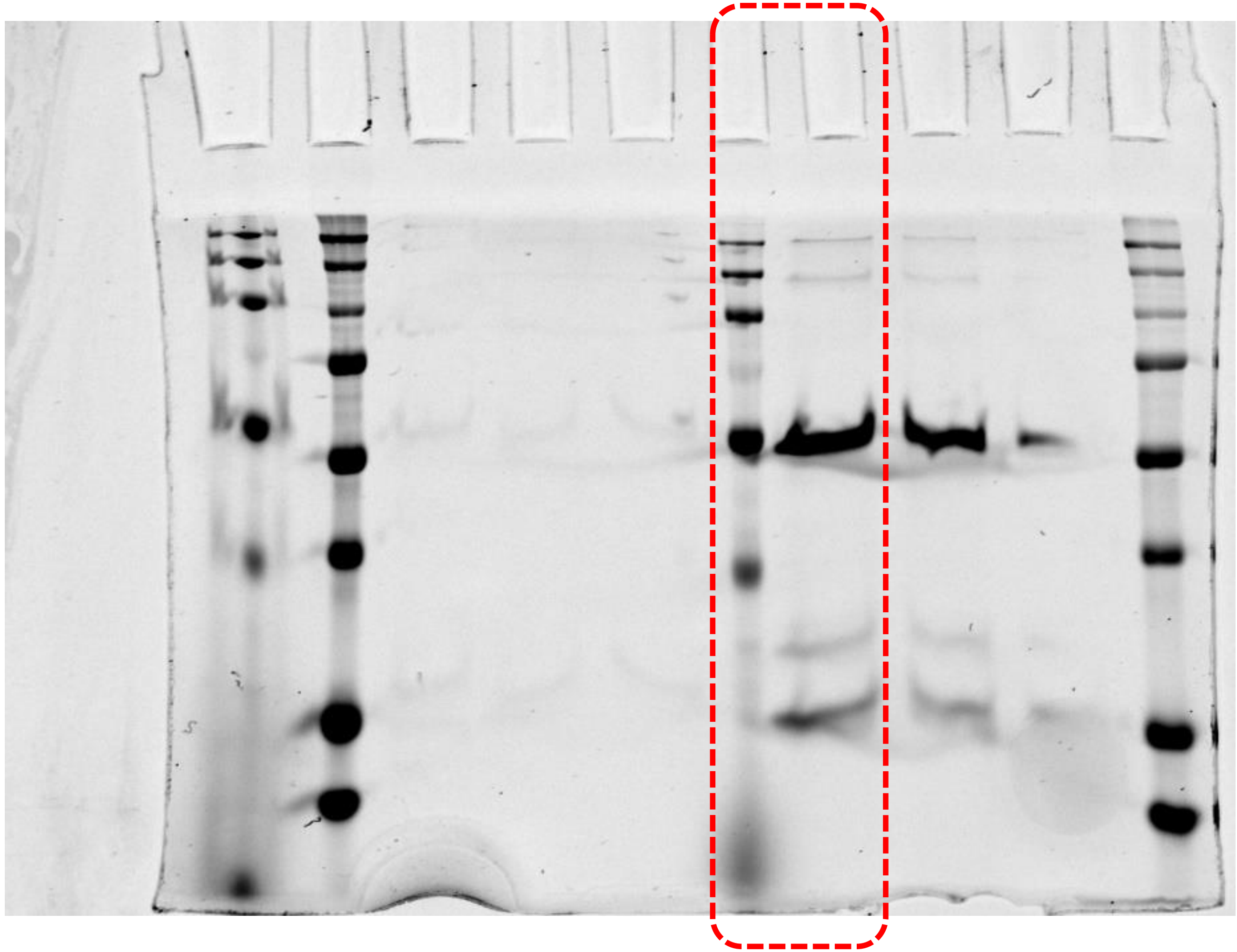

Figure S4D Coomassie in ESI

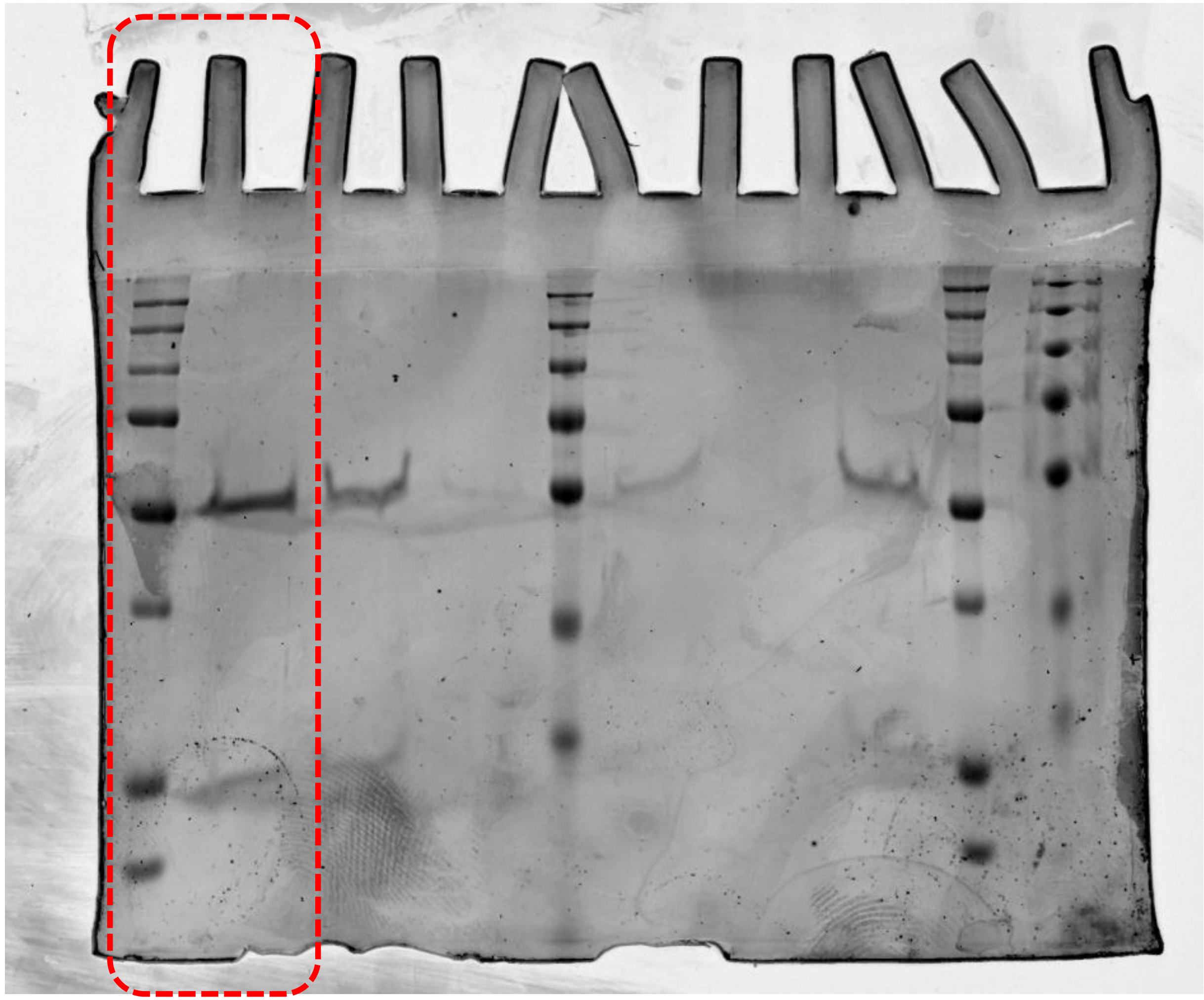

Figure S4E Cy5 in ESI

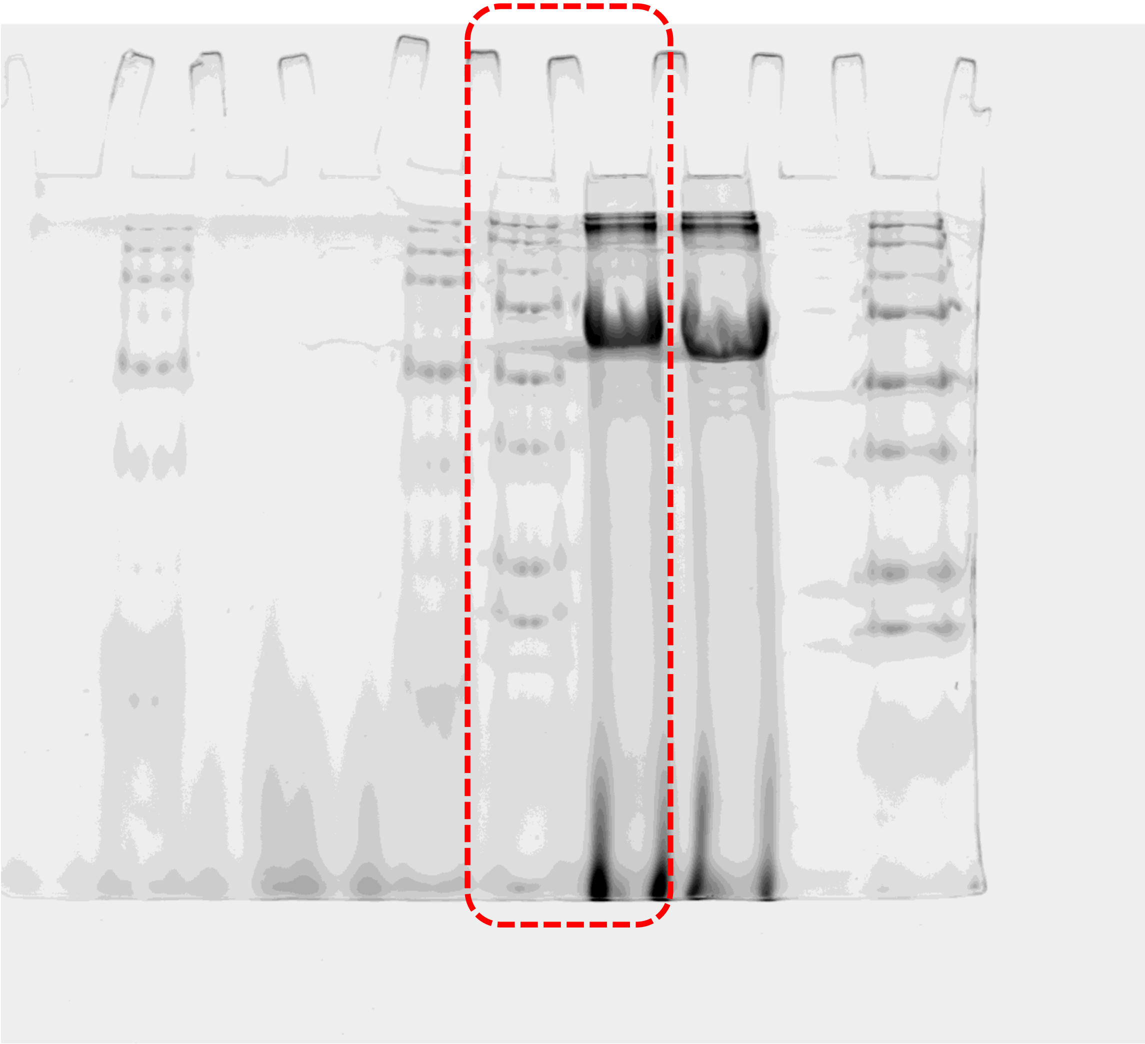

Figure S4E Coomassie in ESI

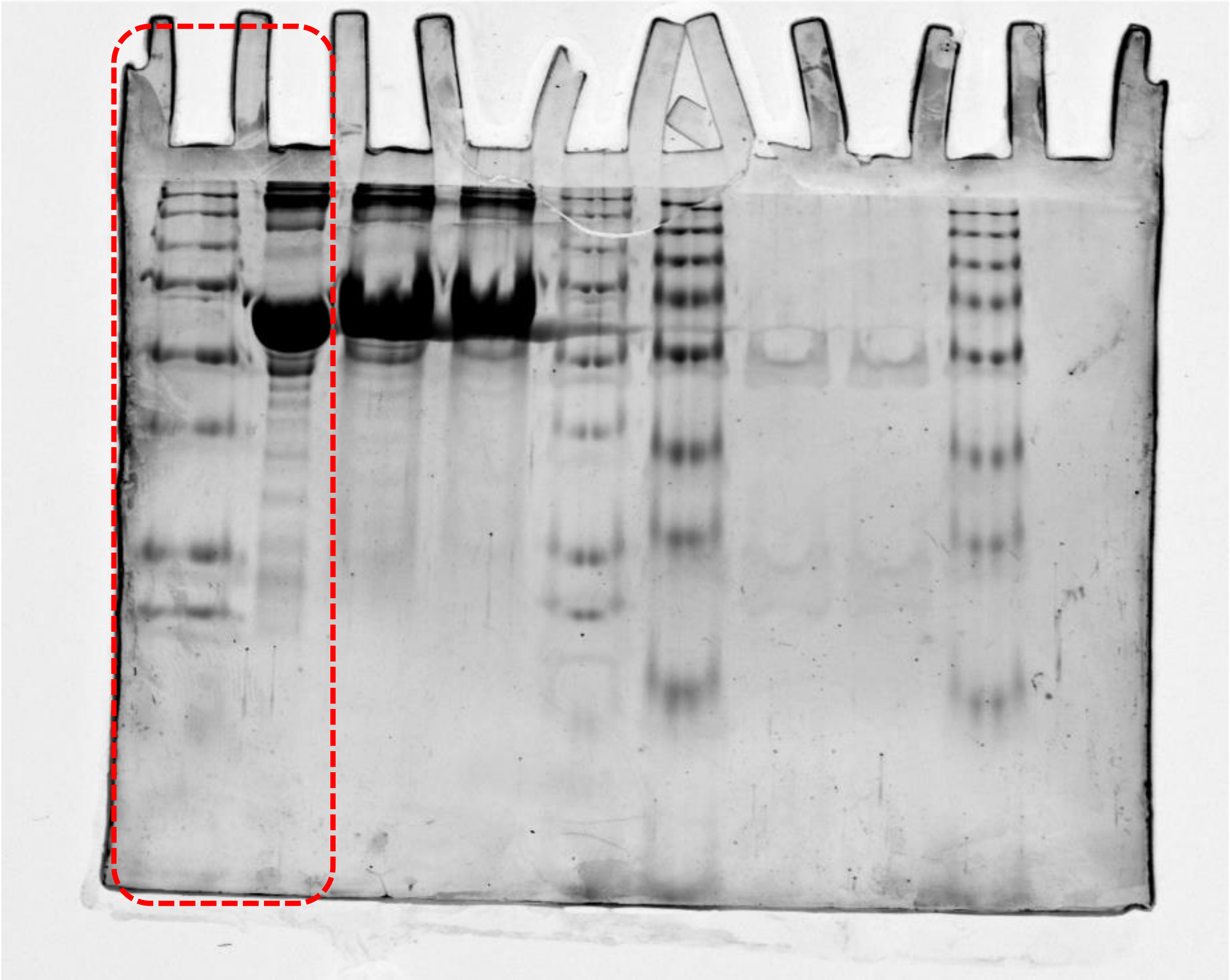

Figure S4F Cy5 channel in ESI

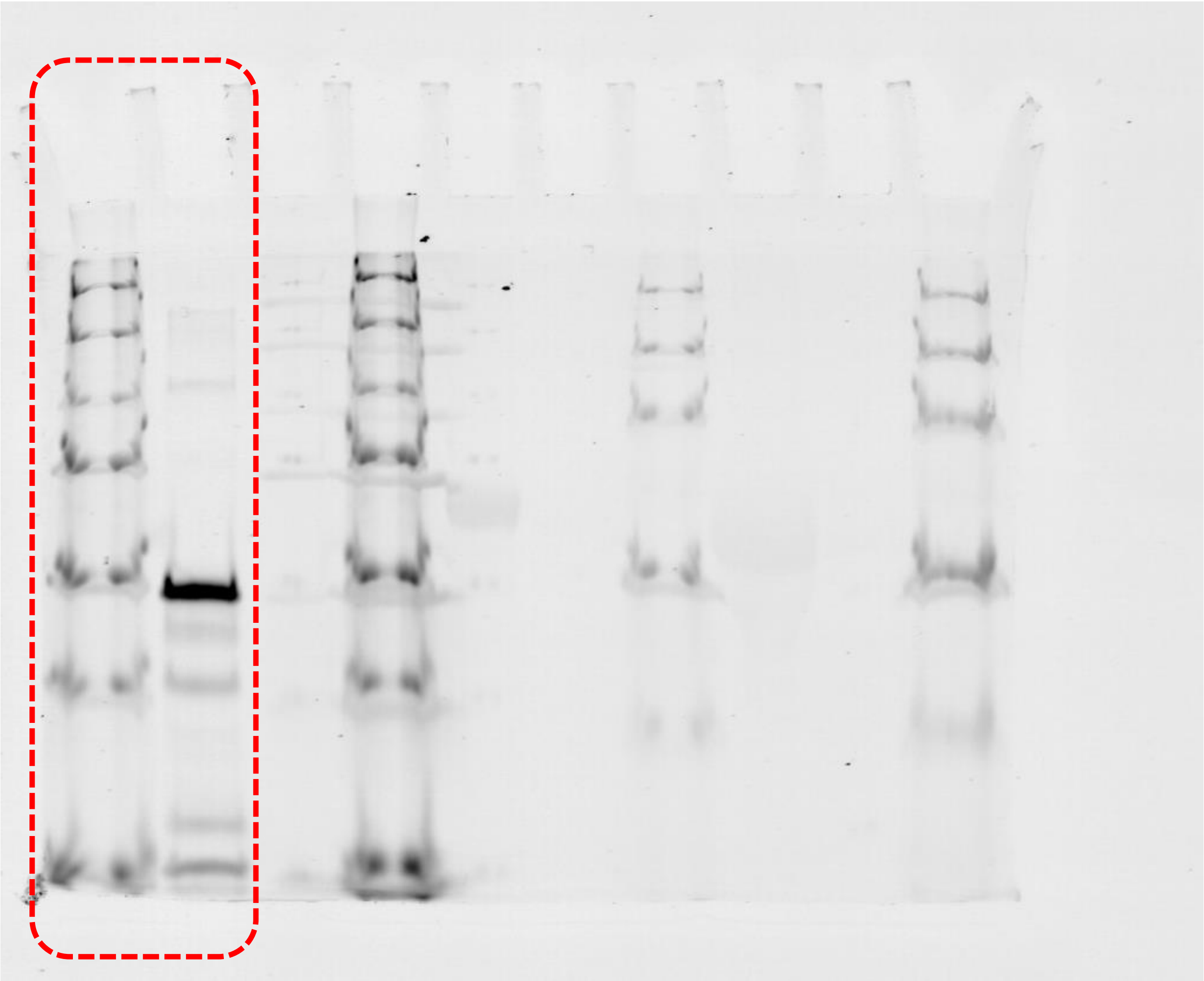

Figure S4E Coomassie in ESI

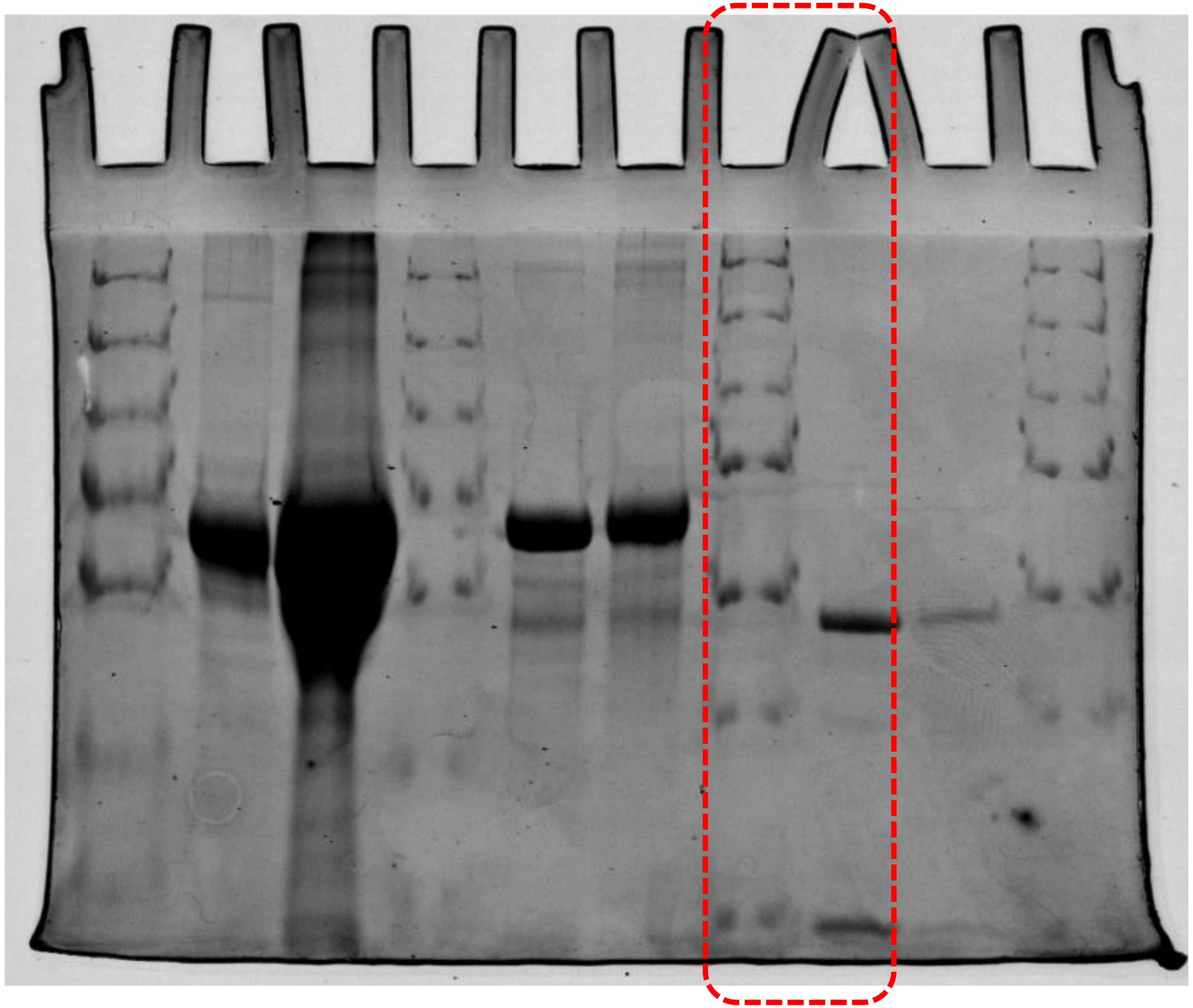

Figure S4G Cy5 channel in ESI

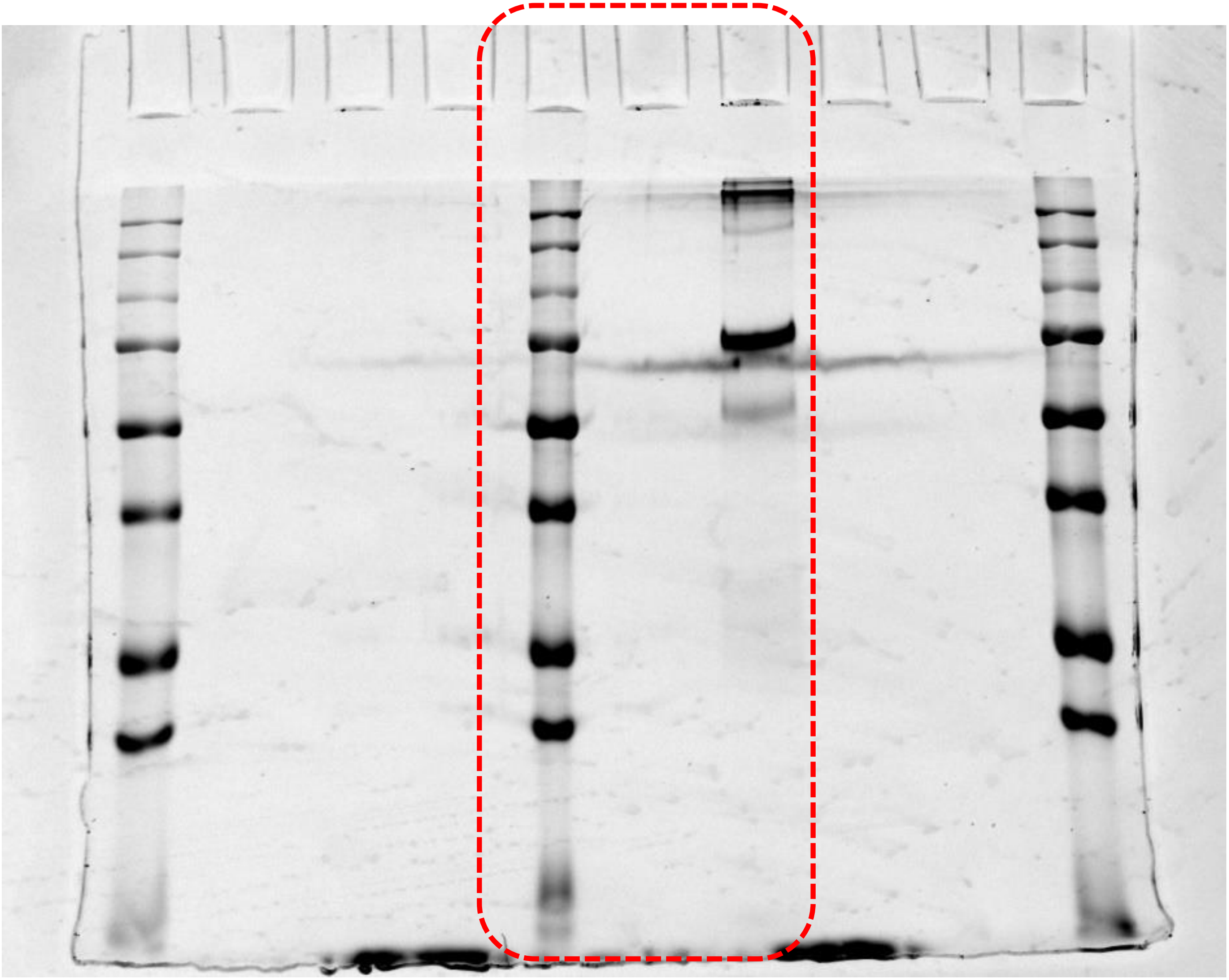

Figure S4G Coomassie in ESI

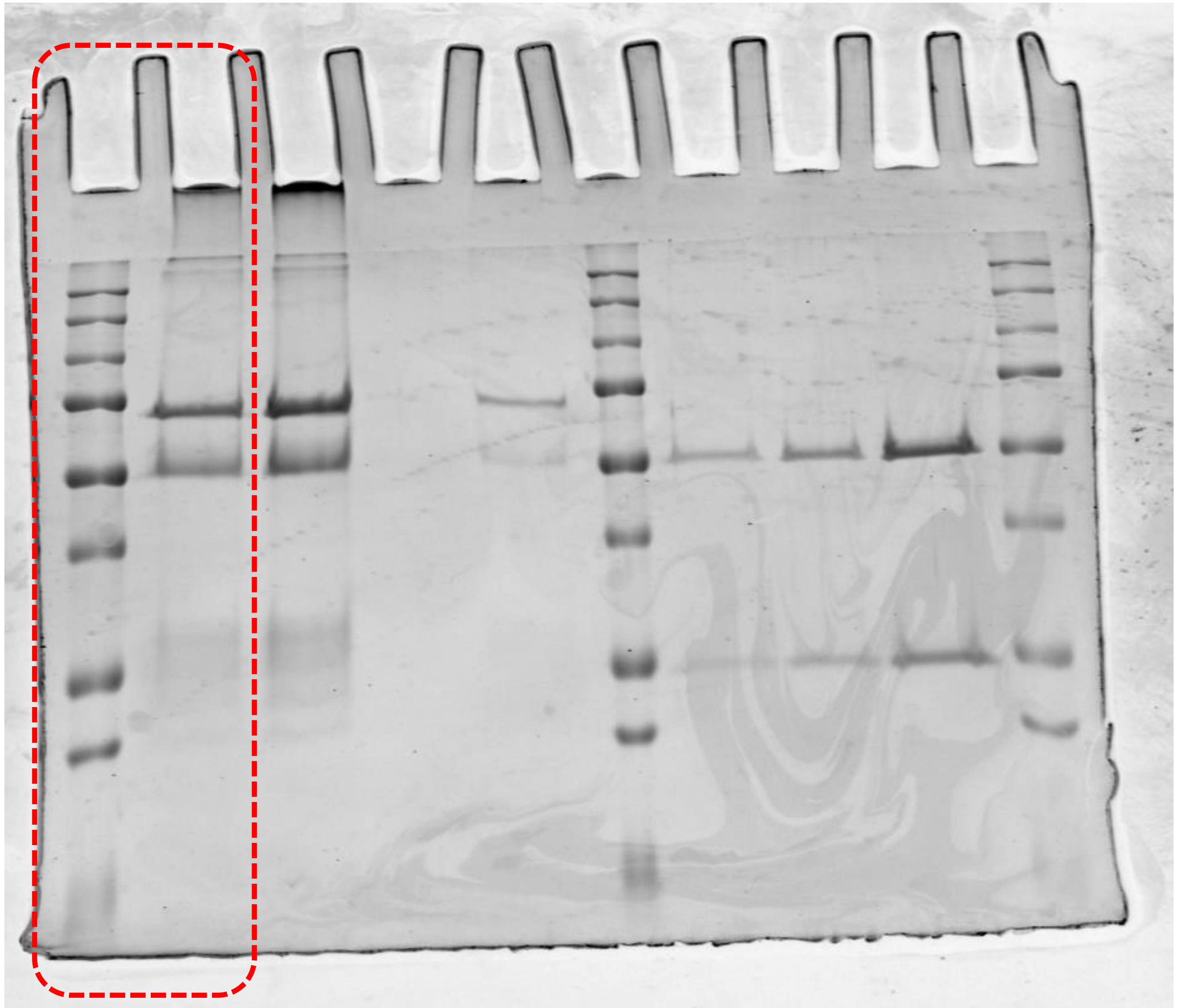

Figure S4H FITC channel in ESI

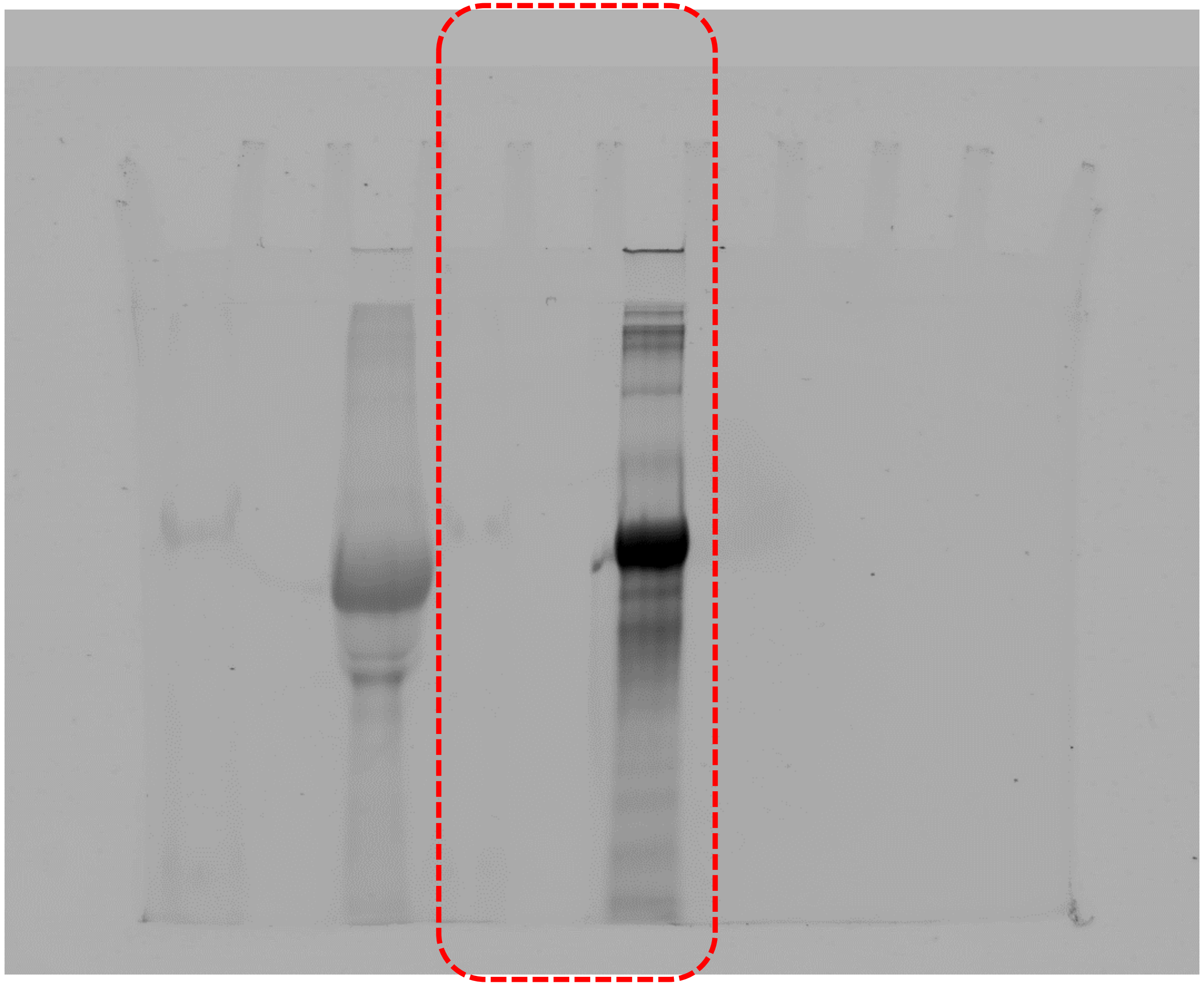

Figure S4H Coomassie in ESI

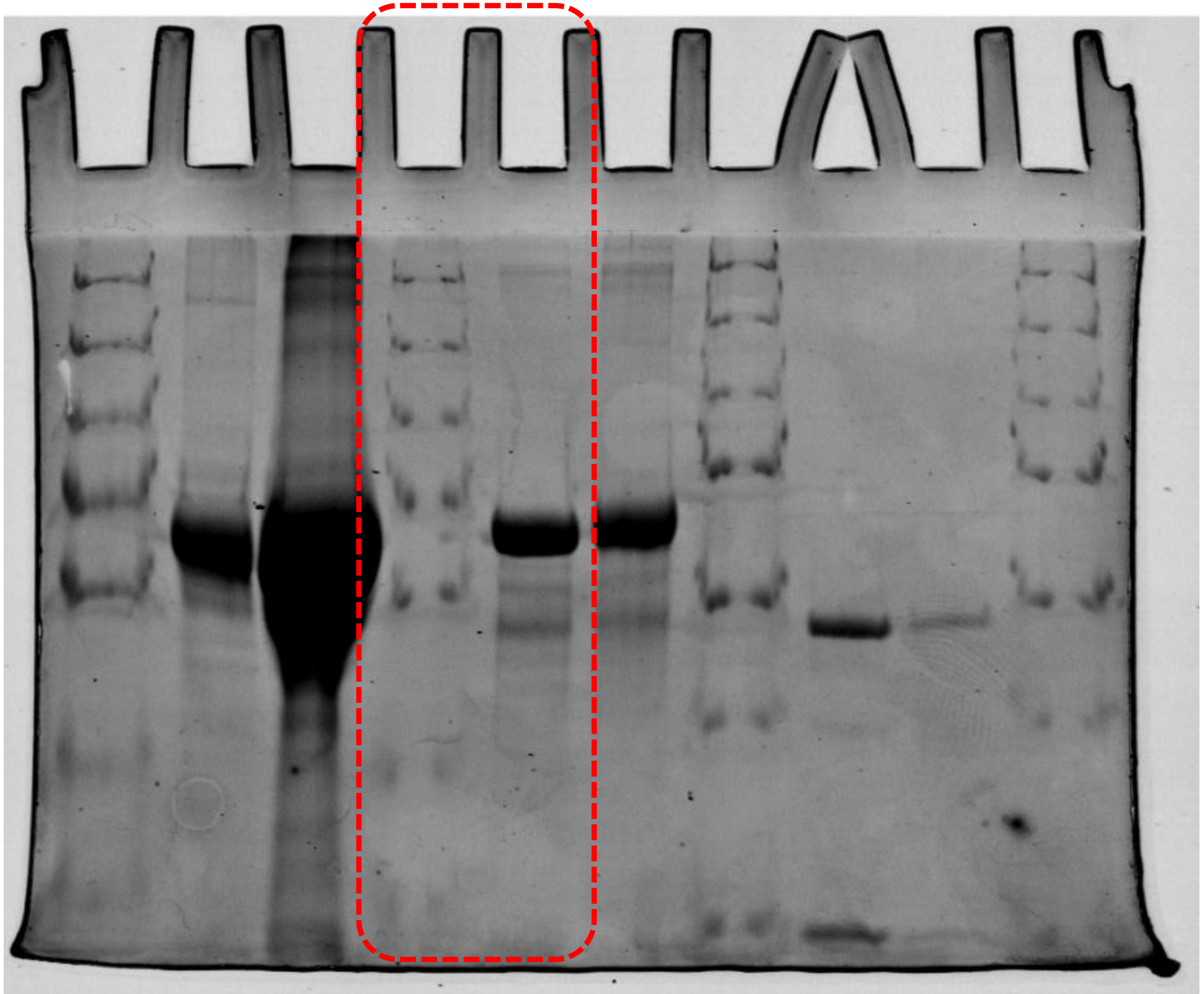

Figure S5A in ESI

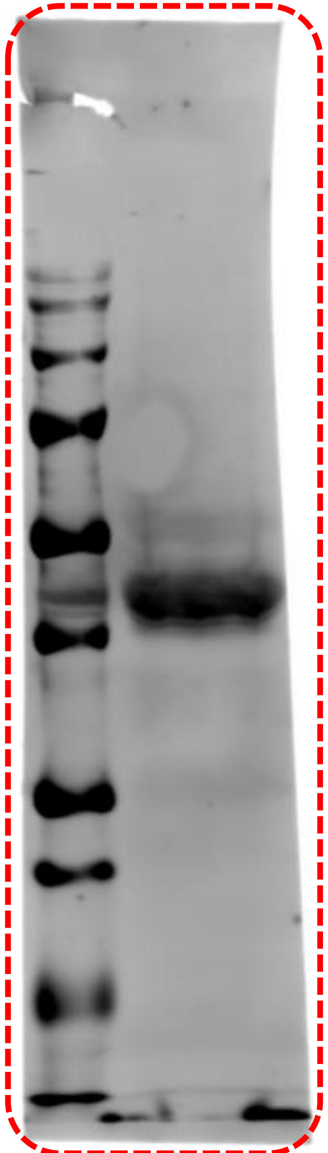

Figure S5B in ESI

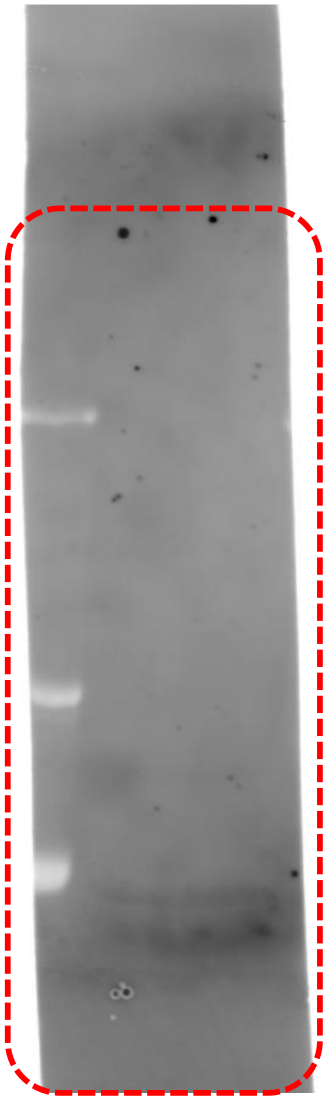

Figure S5C in ESI

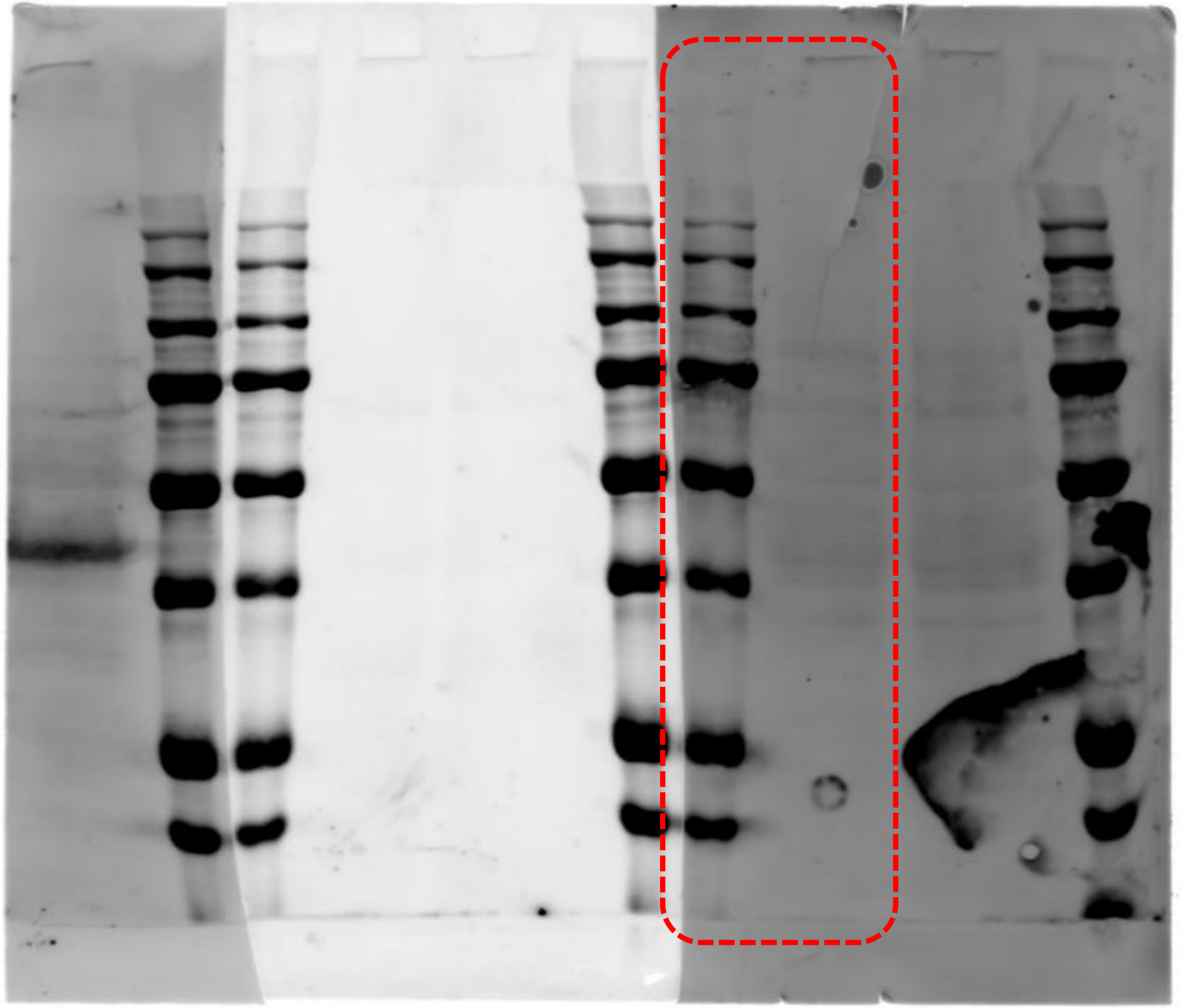

Figure S5D in ESI

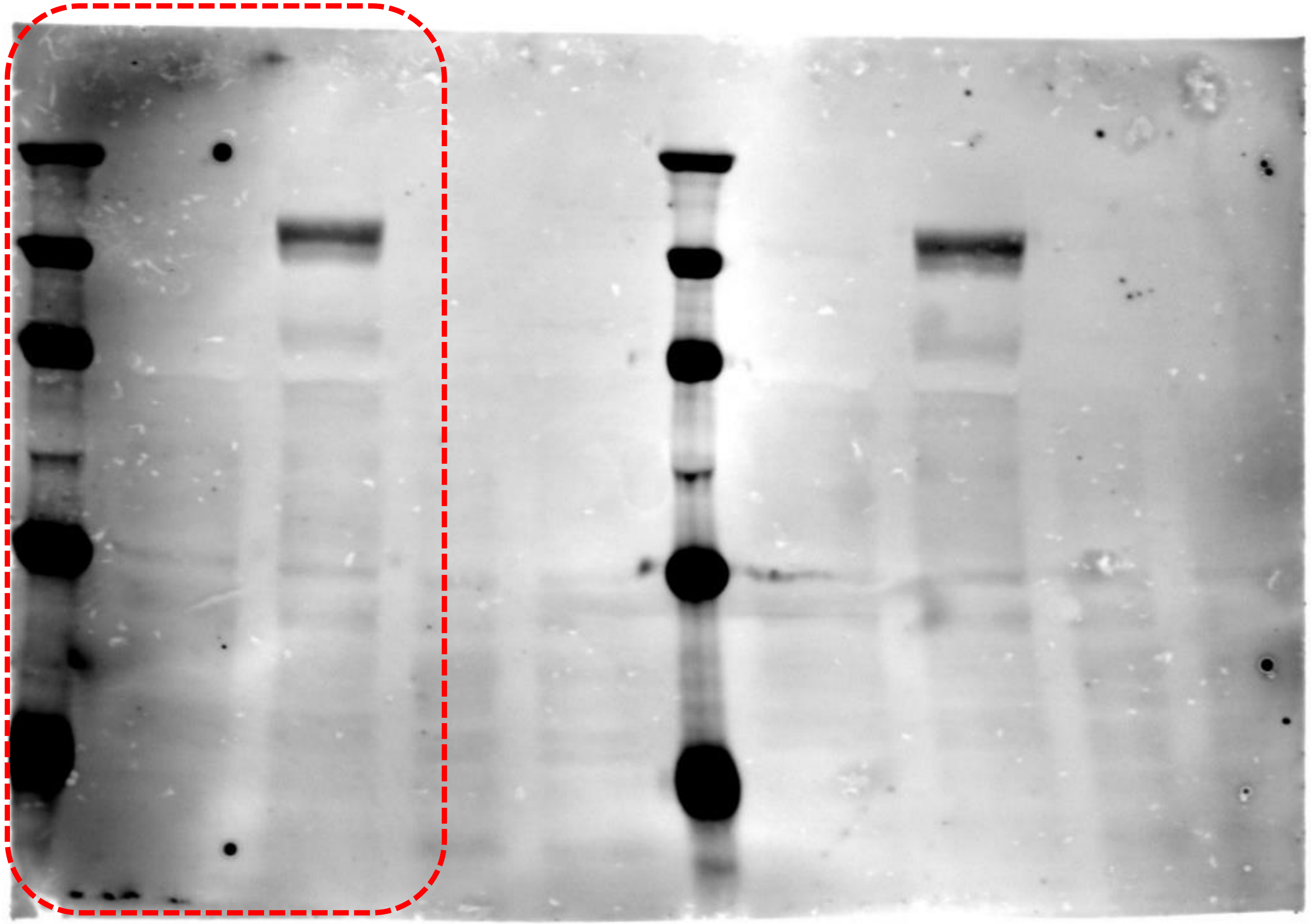

Supplement: RA-012-D2RA05580E-s001 [file RA-012-D2RA05580E-s001.pdf]
