# Supplementary material for: Bioorthogonal site-selective conjugation of fluorescent dyes to antibodies: method and potential applications
Source: RSC Adv. 2022 Oct 5;12(44):28306–17. doi: 10.1039/d2ra05580e (PMC9533196; doi:10.1039/d2ra05580e)
Supplement: RA-012-D2RA05580E-s002 [file RA-012-D2RA05580E-s002.pdf]

Electronic Supplementary Material (ESI) for RSC Advances.
This journal is © The Royal Society of Chemistry 2022

Electronic Supplementary Information

for

Bioorthogonal site-selective conjugation of fluorescent dyes to antibodies: method and potential applications

Philipp Grossenbacher,[a] Maria C. Essers,[a] Joël Moser,[b] Simon A. Singer,[a] Stephanie Häusler,[c] Bruno Stieger,[c] Jean-Sébastien Rougier[a] and Martin Lochner*[a]

[a]*Institute of Biochemistry and Molecular Medicine, University of Bern, Bühlstrasse 28, 3012 Bern, Switzerland*

[b]*Department of Chemistry, Biochemistry and Pharmaceutical Sciences, University of Bern, Freiestrasse 3, 3012 Bern, Switzerland*

[c]*Department of Clinical Pharmacology and Toxicology, University Hospital Zürich, Rämistrasse 100, 8091 Zürich, Switzerland*

Table of Contents

1. Synthesis of SiR-NHS ester (Scheme S1) S2
2. Synthesis of biotin-NHS ester S15
3. Synthesis of azide linkers S17
4. Observed side reactions with NHS-esters (Scheme S2) S22
5. Non-reducing SDS-PAGE of modified anti GAPDH antibody (Figure S1) S23
6. Size exclusion chromatography (SEC) (Figure S2) S24
7. Direct conjugation of anti GAPDH antibody (Figure S3) S25
8. SDS-PAGE of conjugated IgG antibodies (Figure S4) S26
9. Western blot (Figure S5) and direct immunofluorescence (Figure S6) with conjugated IgG antibodies S28
10. Absorption and emission spectra for fluorescent dibromopyridazinediones **8**-**10** S31
11. NMR spectra for final compounds S33
12. Supplementary references S45

---

1. Synthesis of SiR-NHS ester

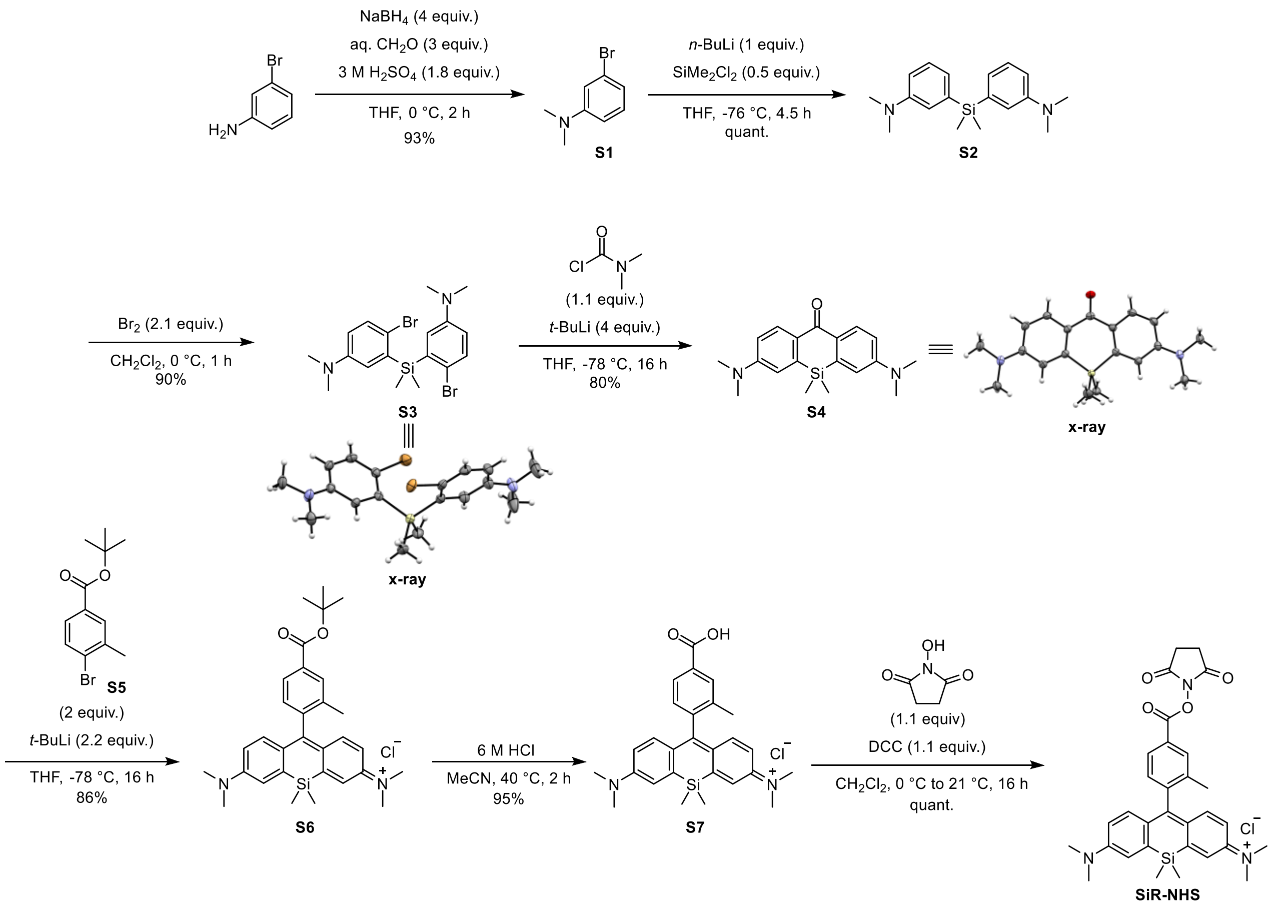

Scheme S1 Synthetic route to SiR-NHS ester.

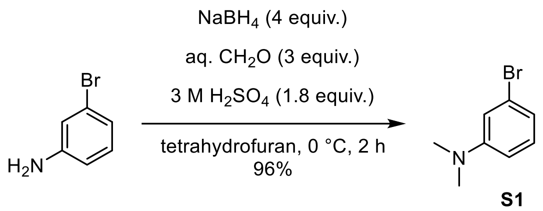

3-bromo-*N*,*N*-dimethylaniline (S1). To a stirred solution of formaldehyde (37% in water, stabilised with MeOH, 10.4 mL, 4.19 g, 139.67 mmol) in THF (125 mL) was added H_2SO_4 (3 M, 28 mL, 84 mmol). The mixture was cooled to -20°C. 3-Bromoaniline (5 mL, 7.9 g, 45.9 mmol) was added dropwise over the course of 10 min. The resulting flaky suspension was stirred until a clear solution formed. $NaBH_4$ was added (7.05 g, 186.36 mmol) portion wise over the course of 30 min ensuring that the temperature remained below 0°C during the addition. After the addition, the reaction mixture was slowly warmed to 23°C and stirred for 1 h. The reaction was quenched by adding sat. $NaHCO_3$ (175 mL) and the mixture was extracted with CH_2Cl_2 (3 x 100 mL). The combined organic phases were washed with brine, dried over $MgSO_4$, filtered and concentrated under reduced pressure. The crude was purified by vacuum Kugelrohrdistillation (1 mbar, 120-145°C). The desired compound was obtained in 96% yield (8.88 g, 44.41 mmol). Physical and spectral data in accordance with literature.[S1]

Yellowish Oil: ^{1}**H NMR** (300 MHz, $CDCl_3$) δ 7.08 (t, J = 8.1 Hz, 1H), 6.87 – 6.79 (m, 2H), 6.55 (dd, J = 8.9, 2.1 Hz, 1H), 2.95 (s, 6H). ^{13}**C NMR** (75 MHz, $CDCl_3$) δ 151.67, 130.37, 123.50, 119.36, 115.31, 111.13, 40.56. **HRMS** (ESI) calculated for $[M+H]^+$ $C_8H_{11}BrN^+$ 200.0069, found 200.0073.

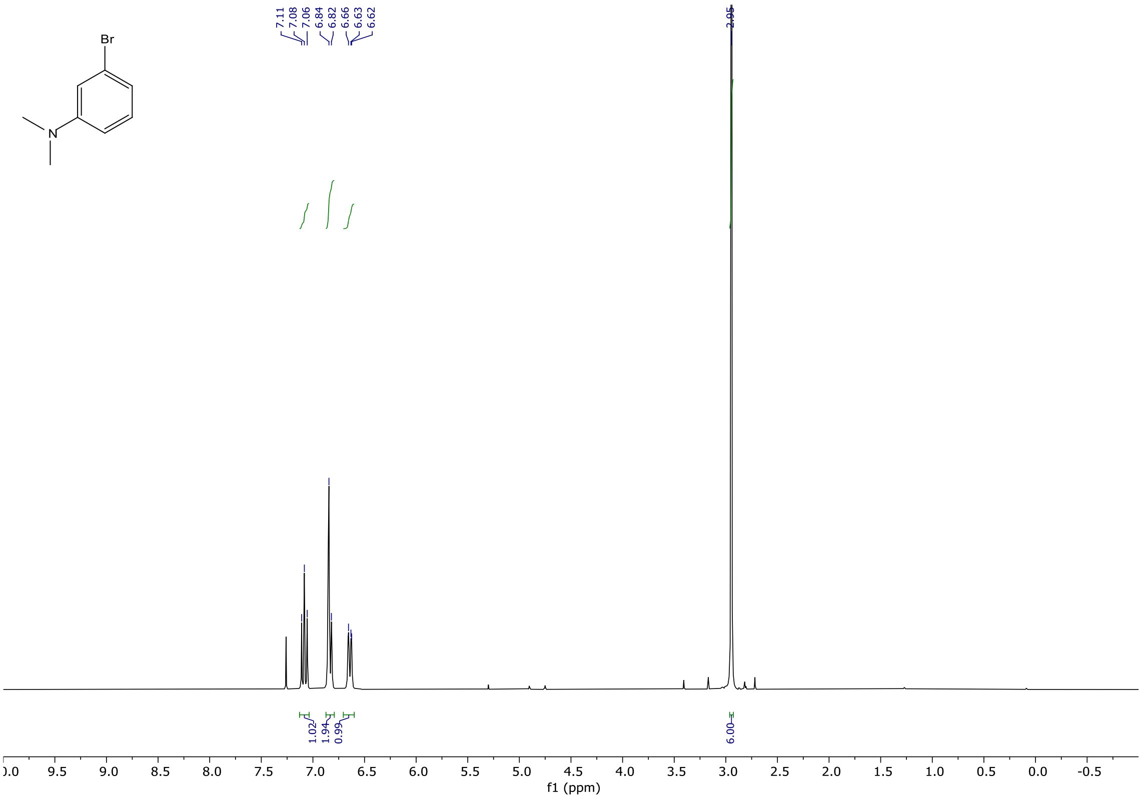

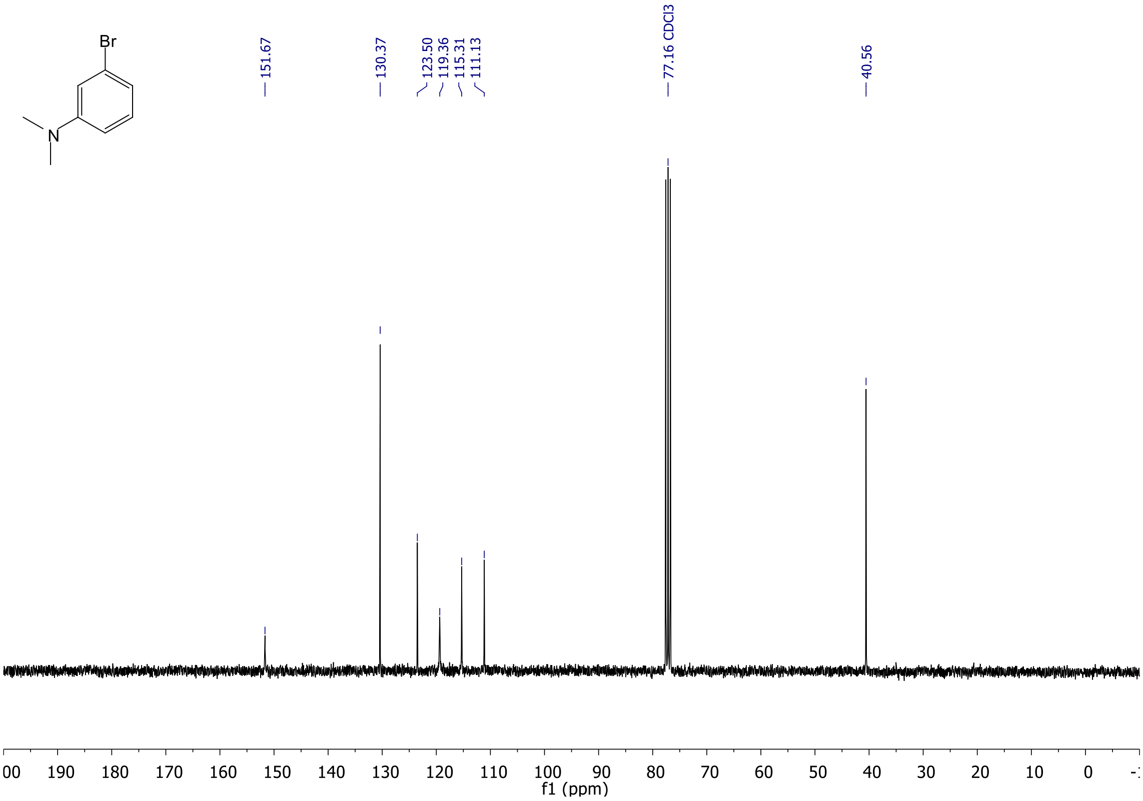

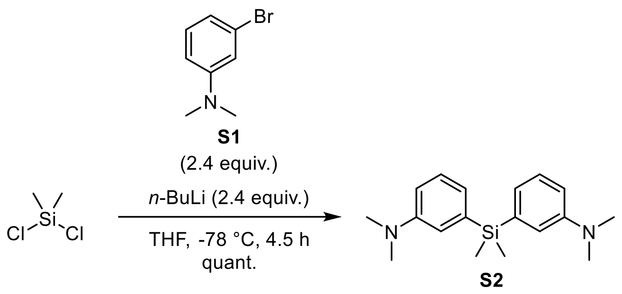

3,3'-(dimethylsilanediyl)bis(*N*,*N*-dimethylaniline) (S2). To a stirred solution of 3-bromo-*N*,*N*-dimethylaniline (**S1**) (4.009 g, 20.036 mmol) in dry THF (50 mL) under an argon atmosphere at -78°C *n*-BuLi (2.5 M in hexanes, 8 mL, 1.281 g, 20.000 mmol) was added dropwise. After the addition, the mixture was stirred for 30 min at -78°C. Then a solution of dichlorodimethylsilane (1.02 mL, 1.07 g, 8.46 mmol) in dry THF (12 mL), pre-cooled to -78°C, was added dropwise. The mixture was slowly warmed to 22°C and stirred for 4 h. To the reaction mixture was added sat. NH_4Cl (5 mL) and water and it was extracted with EtOAc. The combined organic phases were washed with brine, dried over $MgSO_4$, filtered and concentrated under reduced pressure onto silica gel. The crude was purified by flash column chromatography (CombiFlash, cHex/EtOAc gradient from 1:0 to 9:1). The desired compound was obtained in quantitative yield (2.51 g, 8.41 mmol). Physical and spectral data in accordance with literature.[S2] Yellowish Oil**:** **^{1}H NMR** (300 MHz, $CDCl_3$) δ 7.25 (dd, J = 8.0, 7.3 Hz, 2H), 6.94 (d, J = 2.7 Hz, 2H), 6.92 (d, J = 7.1 Hz, 2H), 6.77 (ddd, J = 8.3, 2.7, 0.8 Hz, 2H), 2.93 (s, 12H), 0.54 (s, 6H). **^{13}C NMR** (75 MHz, $CDCl_3$) δ 150.08, 139.10, 128.61, 122.88, 118.48, 113.70, 40.82, -2.01. **HRMS** (ESI) calculated for $[M+H]^+$ $C_{18}H_{27}N_2Si$ 299.1938, found 299.1929.

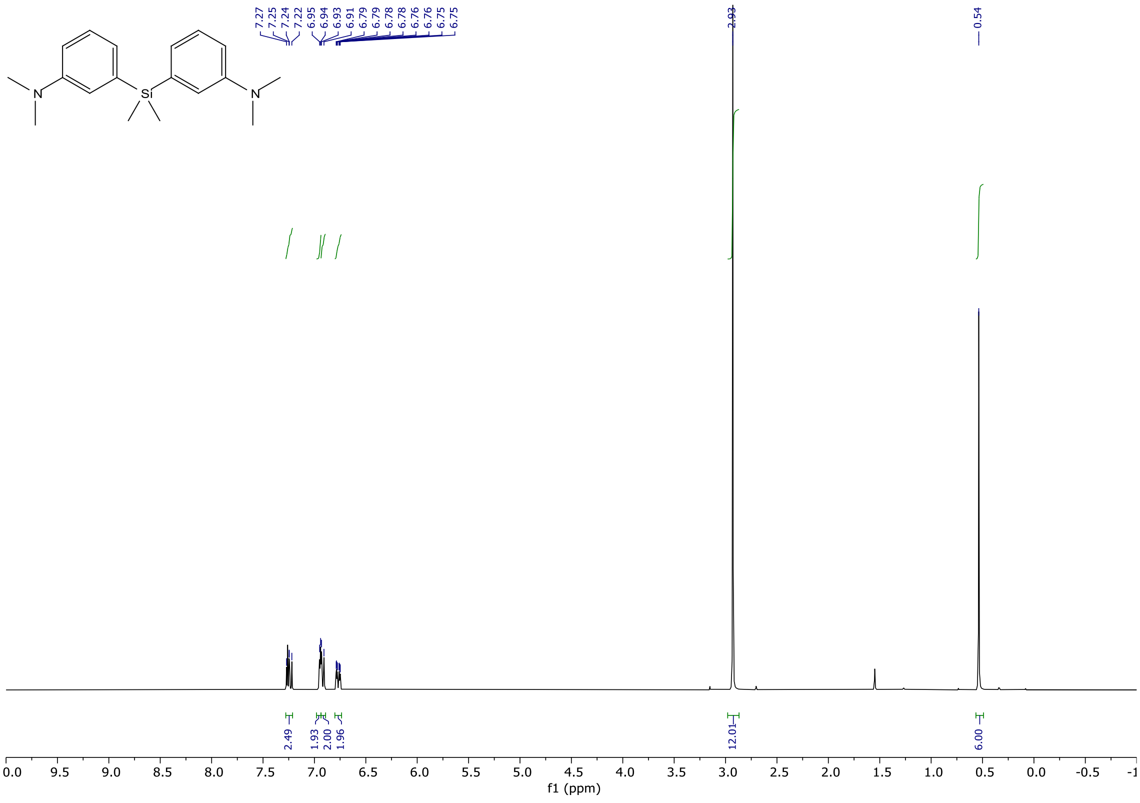

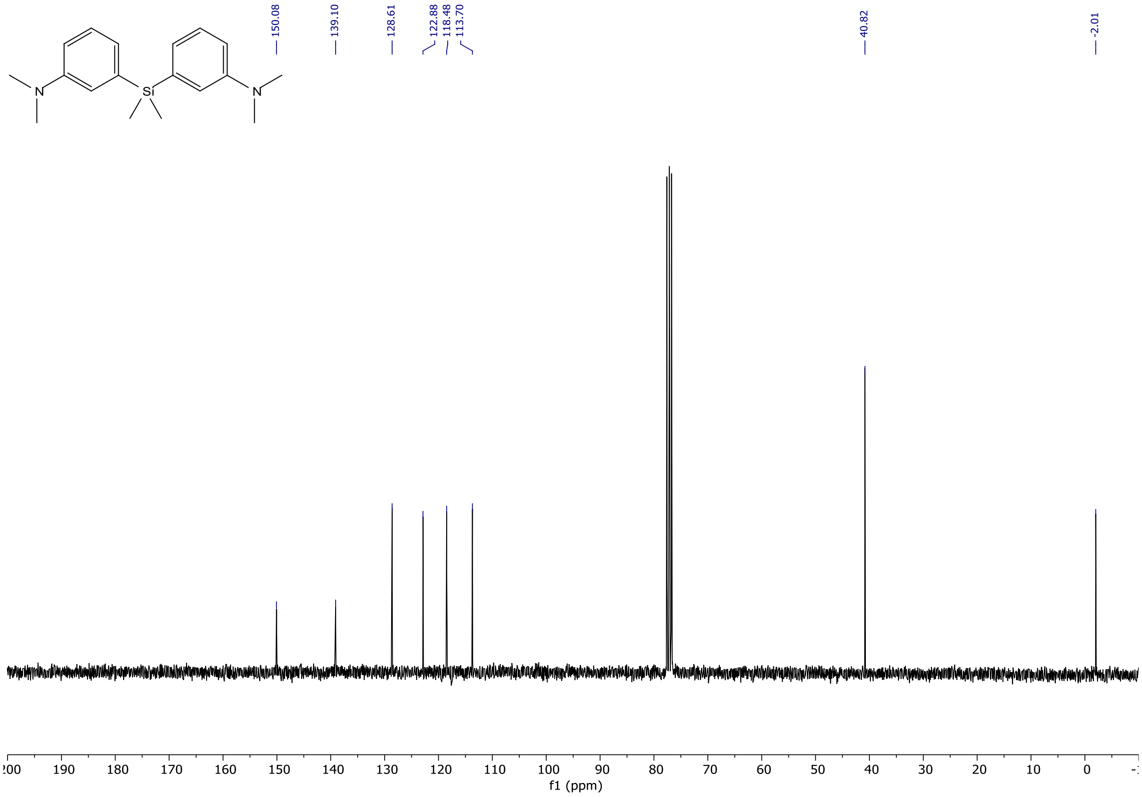

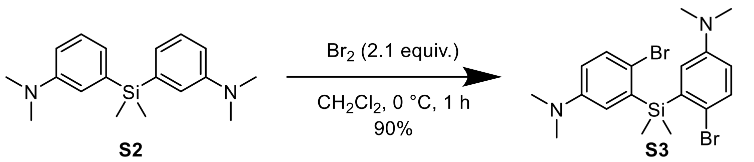

3,3'-(dimethylsilanediyl)bis(4-bromo-*N*,*N*-dimethylaniline) (S3). To a stirred solution of 3,3'-(dimethylsilanediyl)bis(*N*,*N*-dimethylaniline) (**S2**) (3.648 g, 12.221 mmol) in CH_2Cl_2 (40 mL) at 0°C was added bromine (1.3 mL, 4.055 g, 25.372 mmol) over the course of 10 min. The resulting solution was stirred for 1 h whilst slowly warming to room temperature. To the mixture was added aq. $Na_2S_2O_3$. Then the mixture was basified with 2 M aq. NaOH until a clear greenish biphasic mixture was formed. The aqueous phase was extracted with CH_2Cl_2. The combined organic layers were washed with brine, dried over $MgSO_4$, filtered and concentrated under reduced pressure onto silica gel. The crude was purified by flash column chromatography (pentane/Et_2O gradient 1:0 to 9:1) to give the desired compound in 90% yield (5.003 g, 10.964 mmol). A small sample was recrystallised from pentane and Et_2O to obtain single crystals of suitable quality for x-ray diffraction measurements (CCDC deposition number: 2171687). Physical and spectral data in accordance with literature.[S3] Colourless solid**:** **^{1}H NMR** (300 MHz, $CDCl_3$) δ 7.35 (d, J = 8.7 Hz, 2H), 6.85 (d, J = 3.2 Hz, 2H), 6.61 (dd, J = 8.8, 3.2 Hz, 2H), 2.88 (s, 12H), 0.76 (s, 6H). **^{13}C NMR** (75 MHz, $CDCl_3$) δ 148.99, 138.89, 133.12, 121.97, 117.00, 115.45, 40.74, -0.79. **HRMS** (ESI) calculated for $[M+H]^+$ $C_{18}H_{25}Br_2N_2Si^+$ 455.0148, found 455.0146.

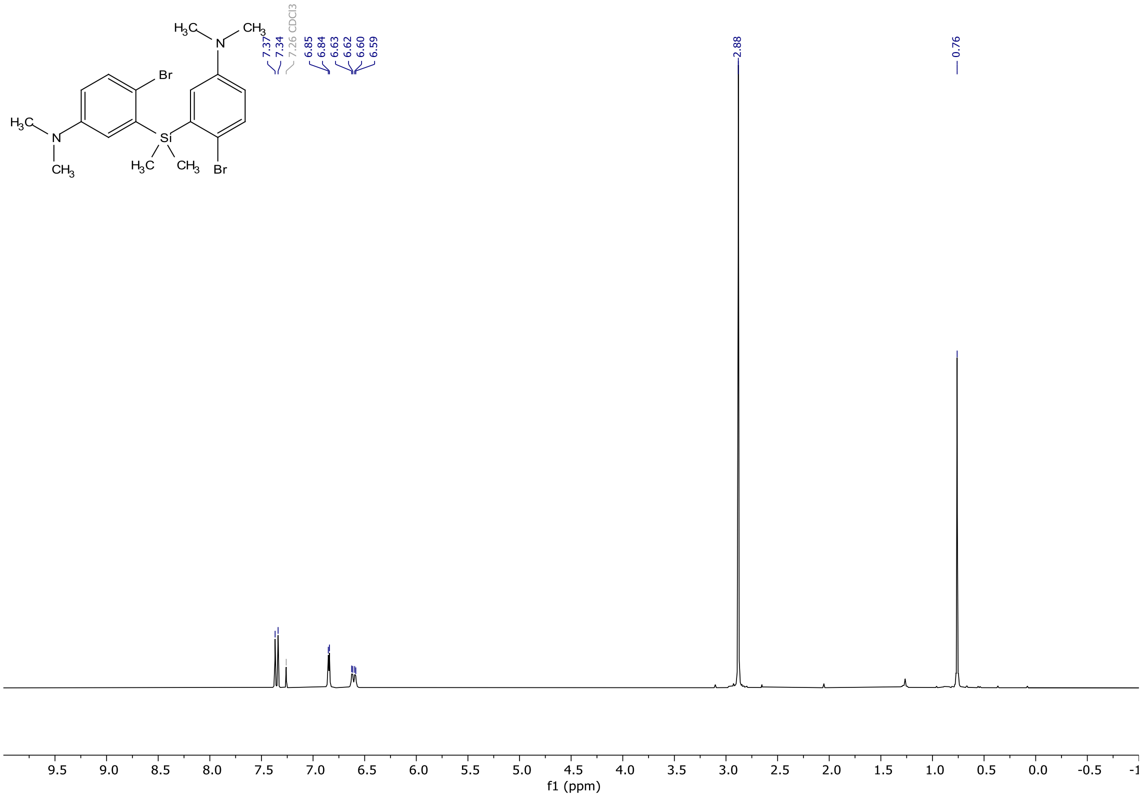
7.37
7.34
7.26 CDCl3
6.85
6.84
6.63
6.62
6.60
6.59
2.88
0.76
f1 (ppm)

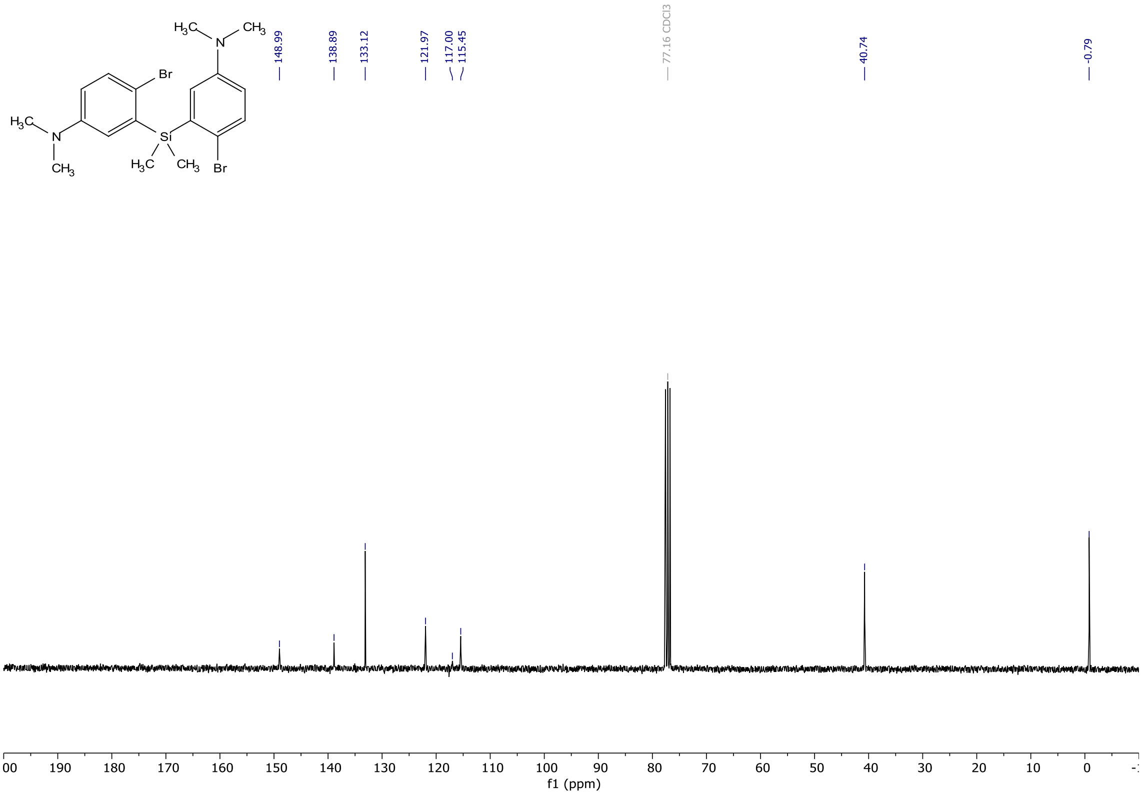
148.99
138.89
133.12
121.97
117.00
115.45
77.16 CDCl3
40.74
-0.79
f1 (ppm)

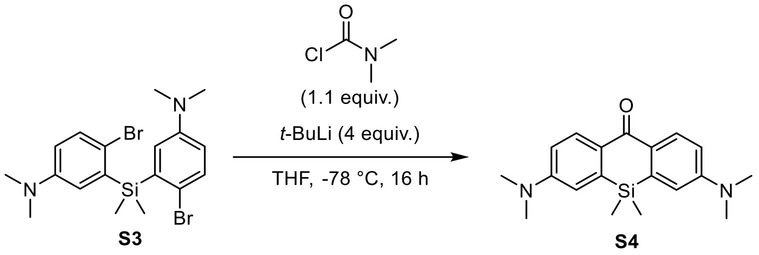

3,7-bis(dimethylamino)-5,5-dimethyldibenzo[*b*,*e*]silin-10(5*H*)-one (S4). To a stirred solution of 3,3'-(dimethylsilanediyl)bis(4-bromo-*N*,*N*-dimethylaniline) (**S3**) (0.67 g, 1.47 mmol) in dry THF (30 mL) at -78°C under an argon atmosphere was added *t*-BuLi (1.7 M in pentane, 3.5 mL, 5.95 mmol) over the course of 4 min. The mixture was stirred at -78°C for 1.5 h. Then was added dropwise dimethylcarbamoyl chloride (0.15 mL, 0.175 g, 1.629 mmol). The mixture was stirred at -78°C for 16 h while slowly warming to room temperature. To the mixture was added aq. sat. NH_4Cl (6 mL) and it was extracted with CH_2Cl_2. The combined organic layers were washed with brine, dried over $MgSO_4$, filtered, and concentrated under reduced pressure onto silica gel. The crude was purified by flash column chromatography (cHex/EtOAc gradient from 1:0 to 0:1) to give the desired compound in 80% yield (0.382 g, 1.178 mmol). A small sample was recrystallised from cHex and EtOAc to obtain single crystals of suitable quality for x-ray diffraction measurements (CCDC deposition number: 2171686). Physical and spectral data in accordance with literature.[S4] Yellow crystals**:** **^{1}H NMR** (300 MHz, $CDCl_3$) δ 8.40 (d, *J* = 8.9 Hz, 2H), 6.95 – 6.73 (m, 4H), 3.09 (s, 12H), 0.47 (s, 6H). **^{13}C NMR** (75 MHz, $CDCl_3$) δ 185.37, 151.49, 140.62, 131.73, 129.90, 114.48, 113.35, 40.21, -0.88. **HRMS** (ESI) calculated for $[M+H]^+$ $C_{19}H_{25}N_2OSi^+$ 325.1731, found 325.1730.

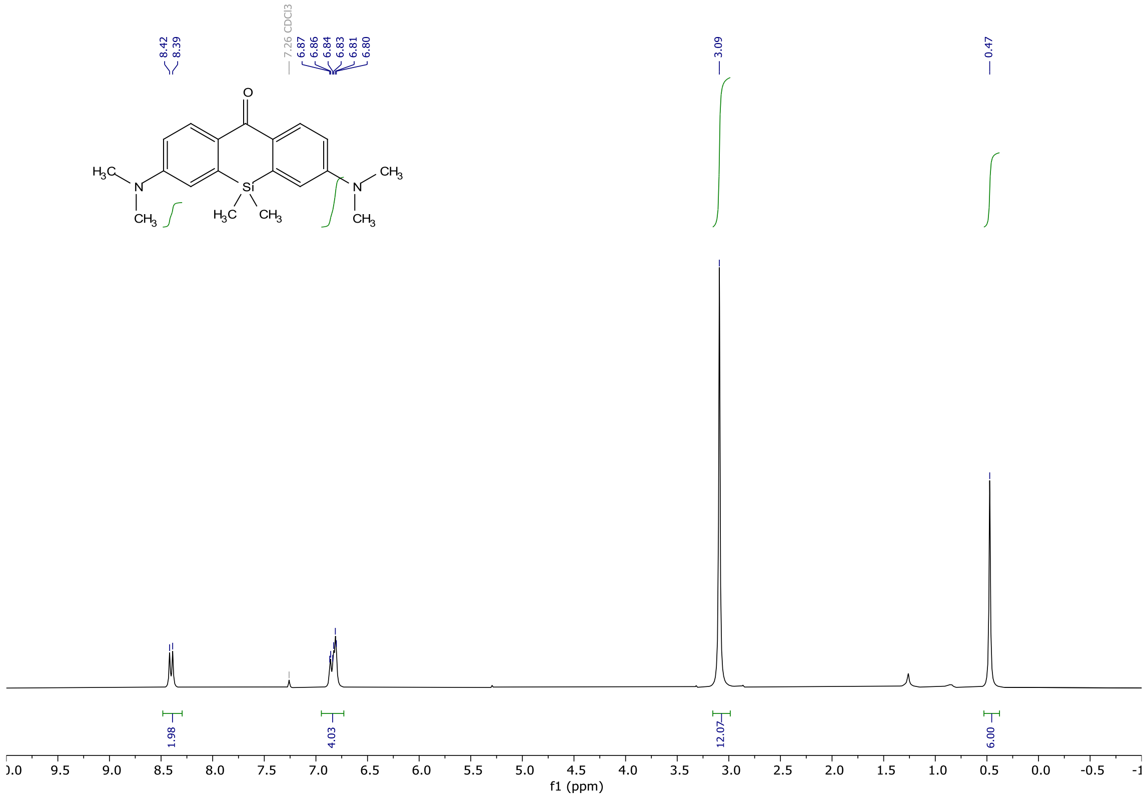

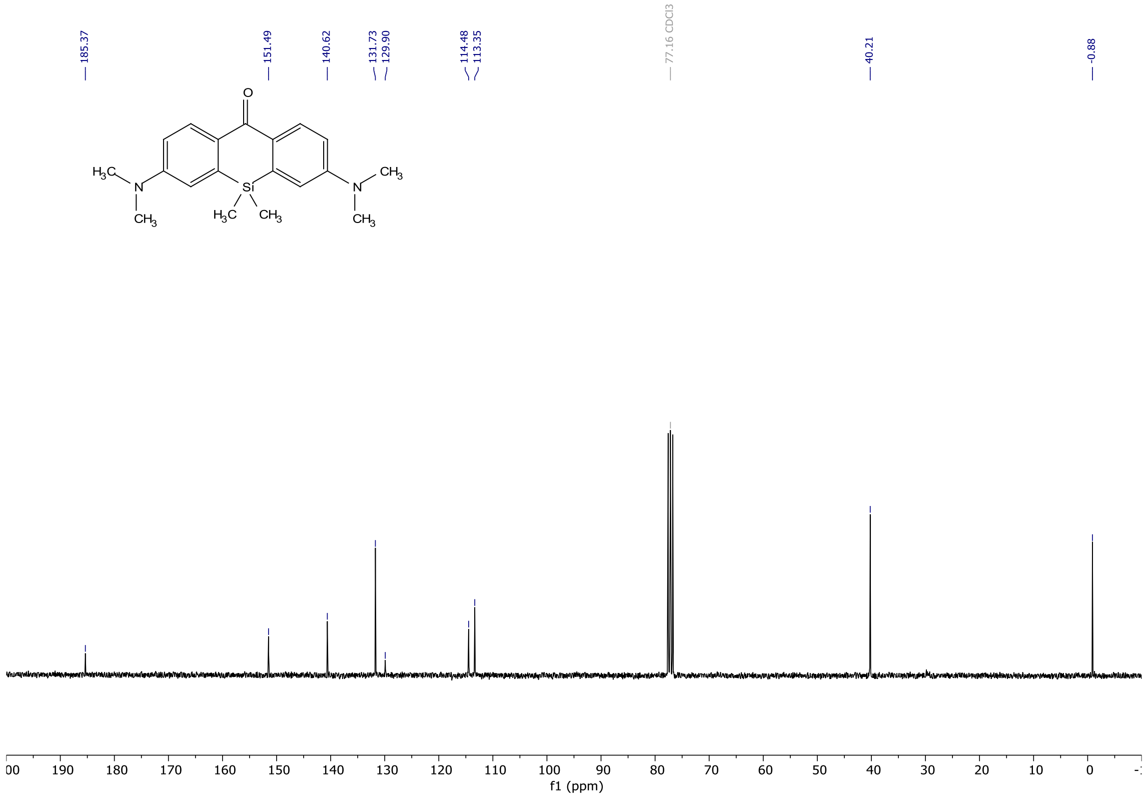

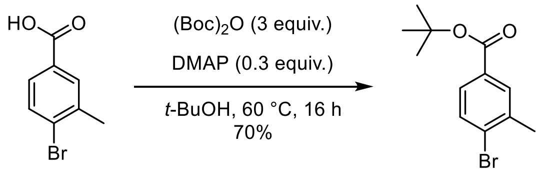

S5

***tert*-butyl 4-bromo-3-methylbenzoate (S5).** To a stirred solution of 4-bromo-3-methylbenzoic acid (3.98 g, 18.52 mmol) in *tert*-Butanol (100 mL) at 60°C was added DMAP (0.627 g, 5.128 mmol). The mixture was stirred for 10 min, then Boc anhydride (12.323 g, 5.646 mmol) in *tert*-Butanol was added dropwise over 30 min. First, the mixture became cloudy, but after the addition of the anhydride the mixture was clear again. It was stirred for 30 min at 60°C. The volatiles were removed under reduced pressure. The colourless solid residue was dissolved in a mixture of aq. $NaHCO_3$ and CH_2Cl_2. The aqueous phase was extracted with CH_2Cl_2, the combined organic layers were washed with brine, dried over $MgSO_4$, filtered and concentrated under reduced pressure onto silica gel. The crude was purified by flash column chromatography (cHex/EtOAc gradient from 1:0 to 0:1) to give the desired compound in 70% yield (3.526 g, 13.002 mmol). Colourless Oil: **^{1}H NMR** (300 MHz, DMSO-d_6) δ 7.83 (d, J = 1.7 Hz, 1H), 7.69 (d, J = 8.3 Hz, 1H), 7.60 (dd, J = 8.3, 1.8 Hz, 1H), 2.39 (s, 3H), 1.53 (s, 9H). **^{13}C NMR** (75 MHz, DMSO-d_6) δ 164.33, 137.81, 132.43, 131.24, 130.69, 129.28, 128.17, 81.05, 39.52, 27.71, 22.32. **HRMS** (ESI) calculated for $[M+Na]^+$ $C_{12}H_{15}O_2BrNa^+$ 293.0148, found 293.0152.

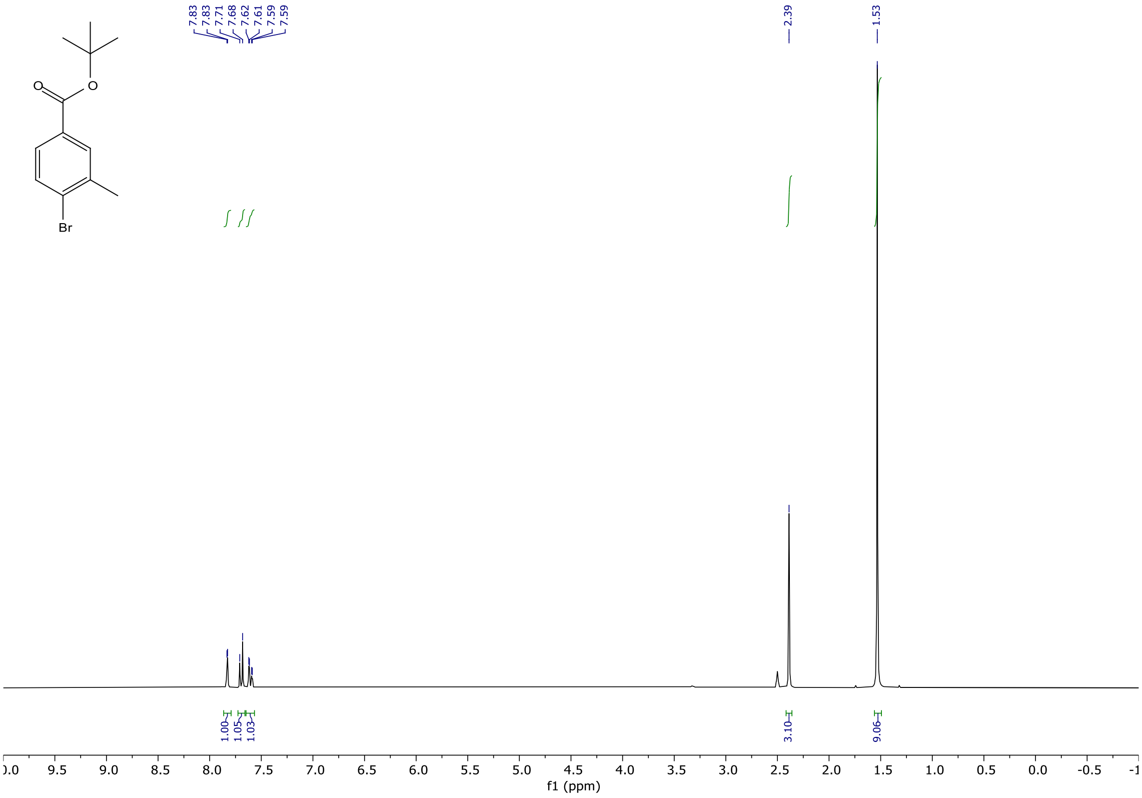

7.83
7.83
7.71
7.68
7.62
7.61
7.59
7.59
2.39
1.53
1.00
1.05
1.03
3.10
9.06
f1 (ppm)
Br
O

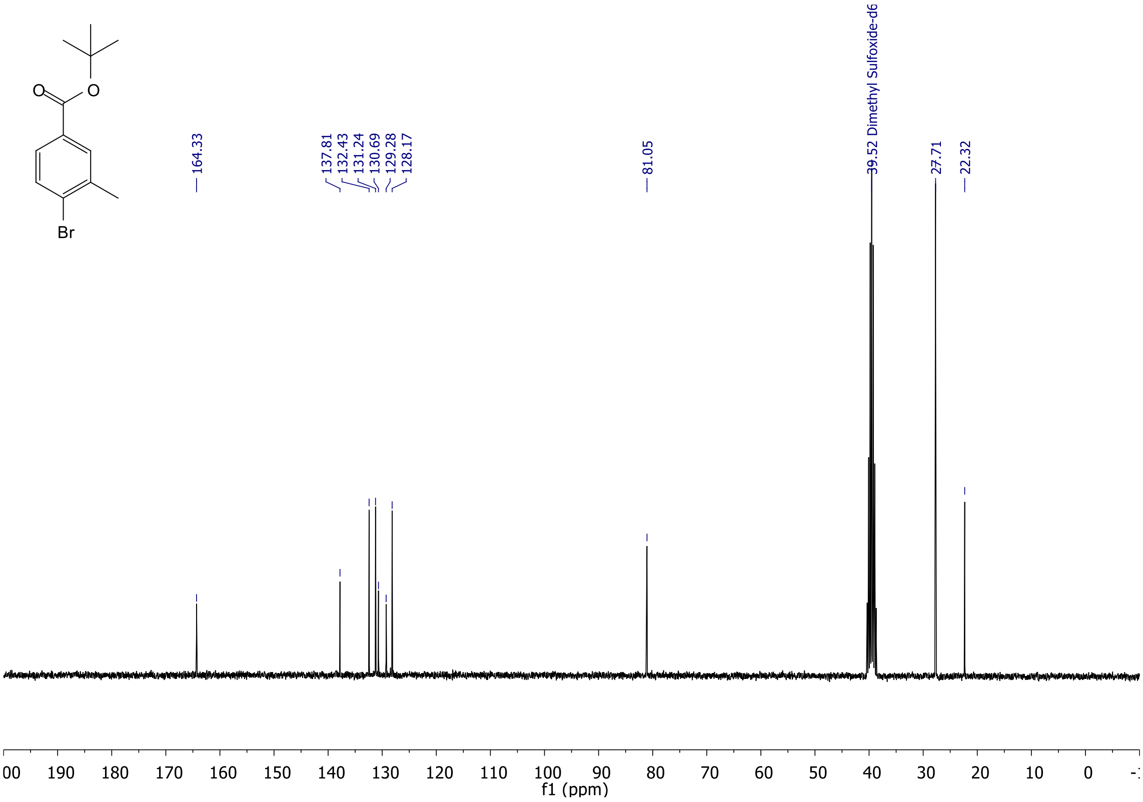

164.33
137.81
132.43
131.24
130.69
129.28
128.17
81.05
39.52 Dimethyl Sulfoxide-d6
27.71
22.32
f1 (ppm)
Br
O

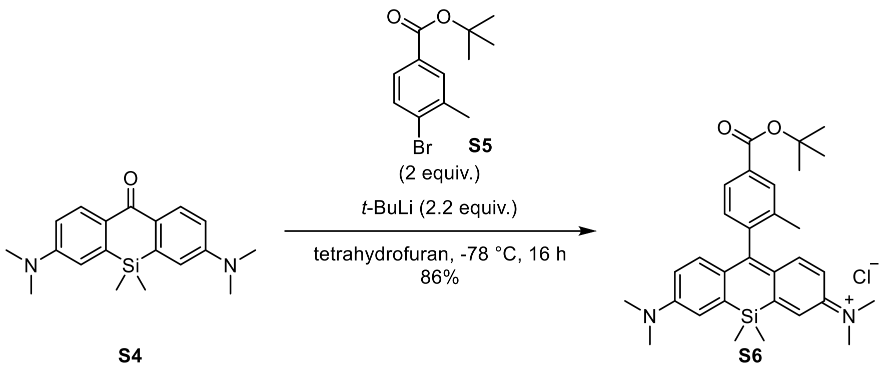

***N*-(10-(4-(tert-butoxycarbonyl)-2-methylphenyl)-7-(dimethylamino)-5,5-dimethyldibenzo[*b*,*e*]silin-3(5*H*)-ylidene)-*N*-methylmethanaminium chloride (S6).** To a stirred solution of (*tert*-butyl 4-bromo-3-methylbenzoate) (**S5**) (0.90 g, 3.32 mmol) in dry THF (10 mL) at -100°C was added dropwise *t*-BuLi (1.7 M in pentane, 2.2 mL, 3.74 mmol). The mixture was stirred at -100°C for 30 min. Then 3,7-bis(dimethylamino)-5,5-dimethyldibenzo[*b*,*e*]silin-10(5*H*)-one (**S4**) (0.546 g, 1.681 mmol) dissolved in dry THF (25 mL) was added dropwise. The mixture was stirred at -100°C for 1 h, after which it was slowly warmed to 23°C and it was stirred for another 24 h. The reaction was quenched by adding aq. HCl (1 M, 75 mL) and the resulting blue mixture was left stirring for 30 min. It was neutralised with Na_2CO_3 until the gas evolution ceased. The mixture was extracted with CH_2Cl_2, the combined organic layers were dried over $MgSO_4$, filtered and concentrated under reduced pressure onto silica gel. The crude was purified by flash column chromatography (CH_2Cl_2/MeOH gradient from 1:0 to 9:1) to give the desired compound in 86% yield (0.772 g, 1.443 mmol). Purple solid: **^{1}H NMR** (400 MHz, CD_3OD) δ 7.99 (s, 1H), 7.96 (d, *J* = 7.9 Hz, 1H), 7.38 (d, *J* = 2.8 Hz, 2H), 7.25 (d, *J* = 7.9 Hz, 1H), 7.02 (d, *J* = 9.7 Hz, 2H), 6.79 (dd, *J* = 9.7, 2.8 Hz, 2H), 3.35 (s, 12H), 2.09 (s, 3H), 1.64 (s, 9H), 0.62 (s, 3H), 0.61 (s, 3H). ^{13}C NMR (101 MHz, CD_3OD) δ 169.18, 166.77, 155.85, 149.50, 144.64, 141.93, 137.63, 133.75, 132.02, 130.50, 127.99, 127.72, 122.34, 115.41, 82.75, 40.94, 28.43, 19.42, -1.11, -1.30. **HRMS** (ESI) calculated for $[M-Cl]^+$ $C_{31}H_{39}N_2O_2Si^+$ 499.2775, found 499.2779.

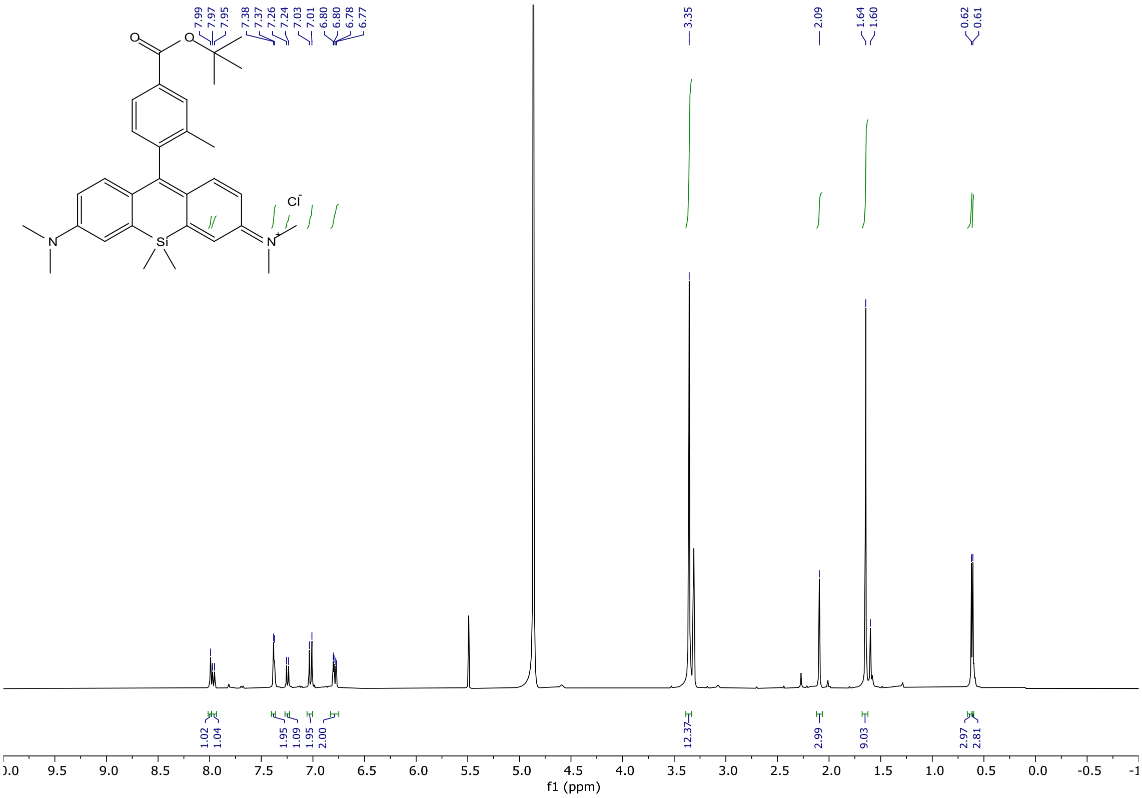
f1 (ppm)

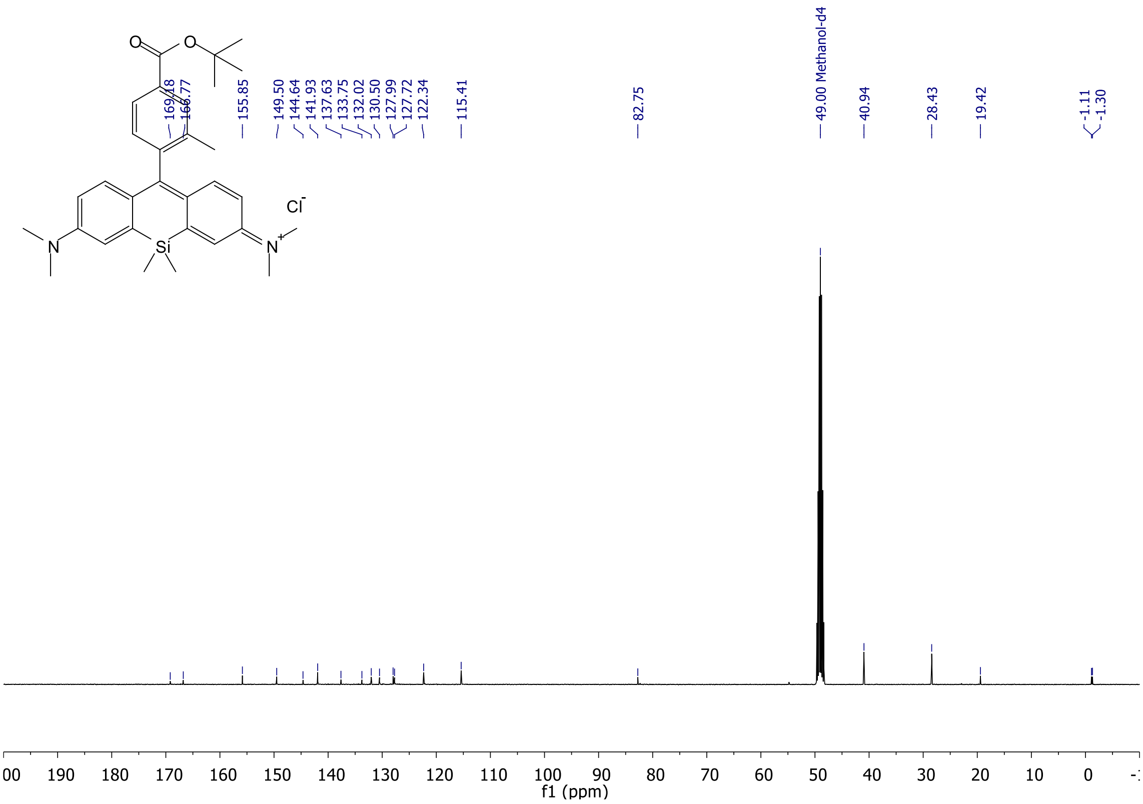
49.00 Methanol-d4
f1 (ppm)

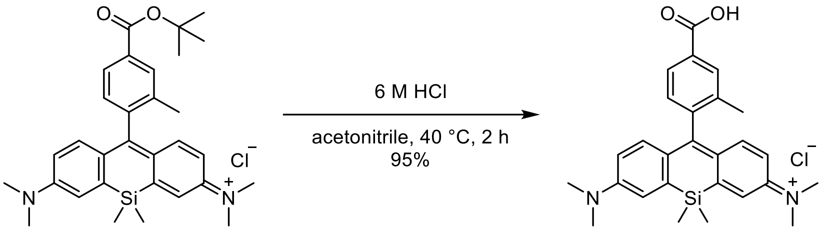

S6 **S7**

***N*-(10-(4-carboxy-2-methylphenyl)-7-(dimethylamino)-5,5-dimethyldibenzo[*b*,*e*]silin-3(5*H*)-ylidene)-*N*-methylmethanaminium chloride (S7).** To a stirred solution of *N*-(10-(4-(*tert*-butoxycarbonyl)-2-methylphenyl)-7-(dimethylamino)-5,5-dimethyldibenzo[*b,e*]silin-3(5*H*)-ylidene)-*N*-methylmethanaminium chloride (**S6**) (0.501 g, 0.937 mmol) in MeCN (40 mL) in a beaker open to air was added aq. HCl (6 M, 0.125 L, 0.75 mol). The blue solution was warmed to 40°C and stirred for 2 h. The solution was cooled to room temperature, extracted with CH_2Cl_2, the combined organic layers were washed with brine, dried over $MgSO_4$, filtered and concentrated under reduced pressure onto silica gel. The crude was purified by flash column chromatography (CH_2Cl_2/MeOH gradient from 1:0 to 9:1) to give the desired product in 95% yield (0.451 g, 0.886 mmol). Physical and spectral data in accordance with literature.[S5] Purple solid: **^{1}H NMR** (300 MHz, CD_3OD) δ 8.06 (s, 1H), 8.01 (d, *J* = 7.9 Hz, 1H), 7.38 (d, *J* = 2.8 Hz, 2H), 7.25 (d, *J* = 7.8 Hz, 1H), 7.03 (d, *J* = 9.6 Hz, 2H), 6.79 (dd, *J* = 9.6, 2.8 Hz, 2H), 3.36 (s, 12H), 2.10 (s, 3H), 0.63 (s, 3H), 0.61 (s, 3H). **^{13}C NMR** (101 MHz, CD_3OD) δ 169.32, 169.22, 155.86, 149.51, 144.76, 141.94, 137.64, 132.84, 132.47, 130.51, 128.09, 128.01, 122.34, 115.41, 40.94, 19.41, -1.09, -1.31. **HRMS** (ESI) calculated for $[M-Cl]^+$ $C_{27}H_{31}N_2O_2Si^+$ 443.2149, found 443.2146.

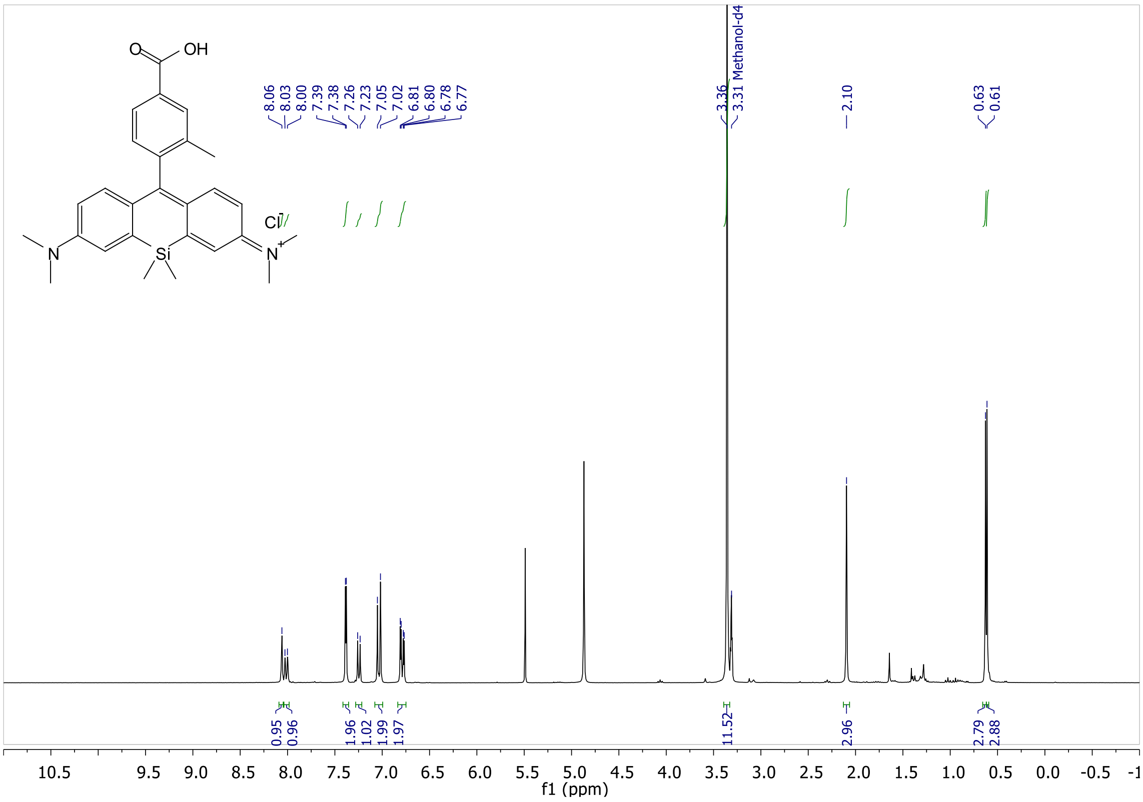

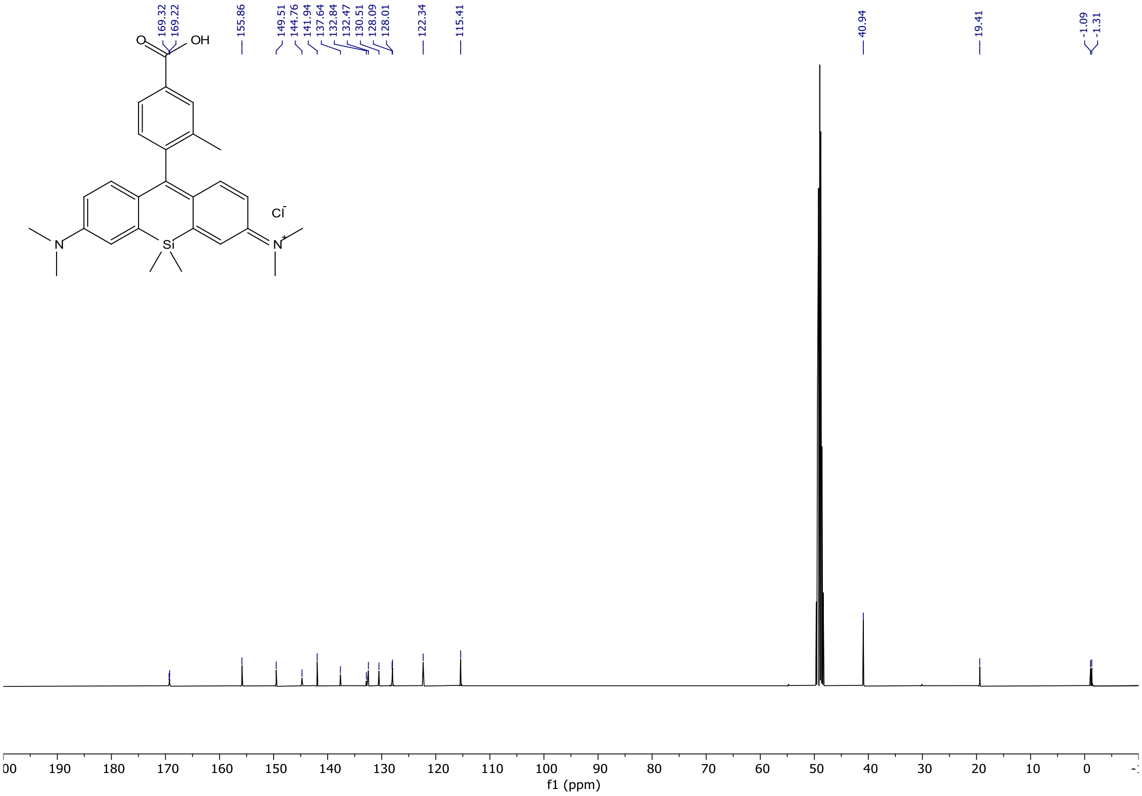

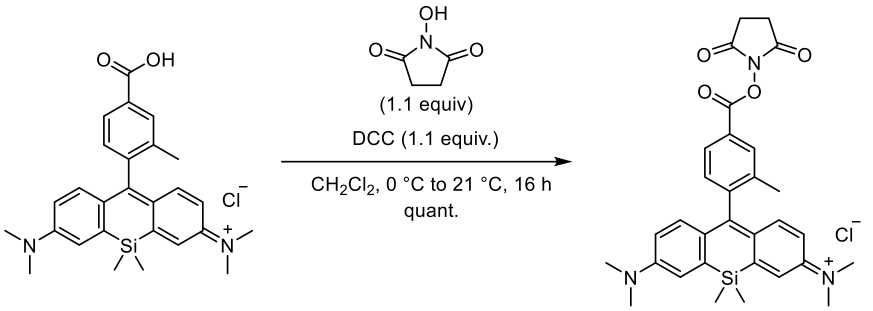

S7 **SiR-NHS**

***N*-(7-(dimethylamino)-10-(4-(((2,5-dioxopyrrolidin-1-yl)oxy)carbonyl)-2-methylphenyl)-5,5-dimethyldibenzo[*b,e*]silin-3(5*H*)-ylidene)-*N*-methylmethanaminium chloride (SiR-NHS).** To a stirred solution of *N*-(10-(4-carboxy-2-methylphenyl)-7-(dimethylamino)-5,5-dimethyldibenzo[*b*,*e*]silin-3(5*H*)-ylidene)-*N*-methylmethanaminium chloride (**S7**) (0.099 g, 0.206 mmol) in dry CH_2Cl_2 (5 mL) at 0°C was added *N*-hydroxysuccinimide (0.037 g, 0.227 mmol) followed by *N*,*N*′-Dicyclohexylcarbodiimid (0.048 g, 0.234 mmol). The mixture was slowly warmed to 21°C and then stirred for 16 h. The mixture was concentrated under reduced pressure onto silica gel. The crude was purified by flash column chromatography (CH_2Cl_2/MeOH gradient from 1:0 to 9:1) to give the desired compound in quantitative yield (0.118 g, 0.204 mmol). Purple solid: **^{1}H NMR** (300 MHz, $CDCl_3$) δ 8.16 – 8.06 (m, 2H), 7.31 – 7.26 (m, 3H), 6.92 (d, *J* = 9.6 Hz, 2H), 6.61 (dd, *J* = 9.6, 2.8 Hz, 2H), 3.43 (s, 12H), 2.96 (s, 4H), 2.11 (s, 3H), 0.66 (s, 3H), 0.64 (s, 3H). **^{13}C NMR** (75 MHz, $CDCl_3$) δ 169.34, 166.36, 161.48, 154.32, 148.62, 145.73, 140.72, 137.33, 132.37, 129.85, 128.05, 126.72, 125.87, 121.62, 114.35, 41.43, 25.87, 19.48, -0.49, -0.76. **HRMS** (ESI) calculated for $[M\text{-}Cl]^+$ $C_{31}H_{34}N_3O_4Si^+$ 540.2313, found 540.2293. **Purity (HPLC):** 95% UV_{214}, 95% UV_{254}.

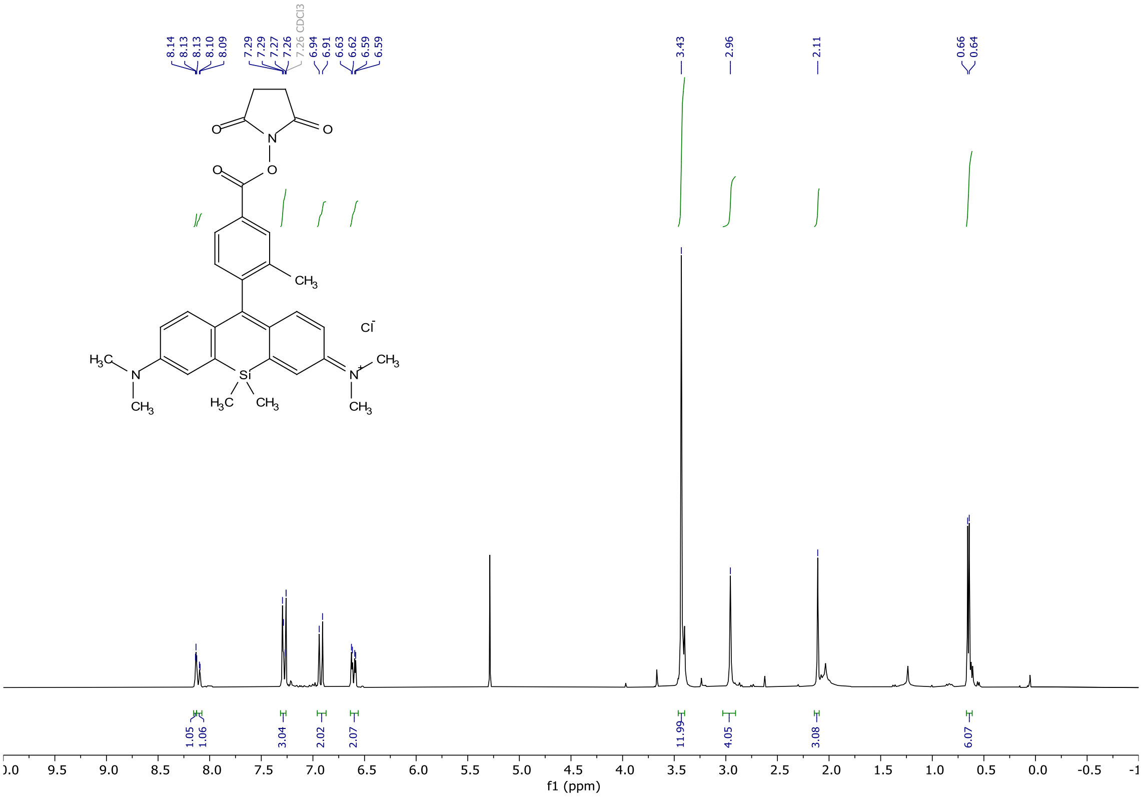
8.14
8.13
8.13
8.10
8.09
7.29
7.29
7.27
7.26
7.26 CDCl3
6.94
6.91
6.63
6.62
6.59
6.59
3.43
2.96
2.11
0.66
0.64
1.05
1.06
3.04
2.02
2.07
11.99
4.05
3.08
6.07
f1 (ppm)

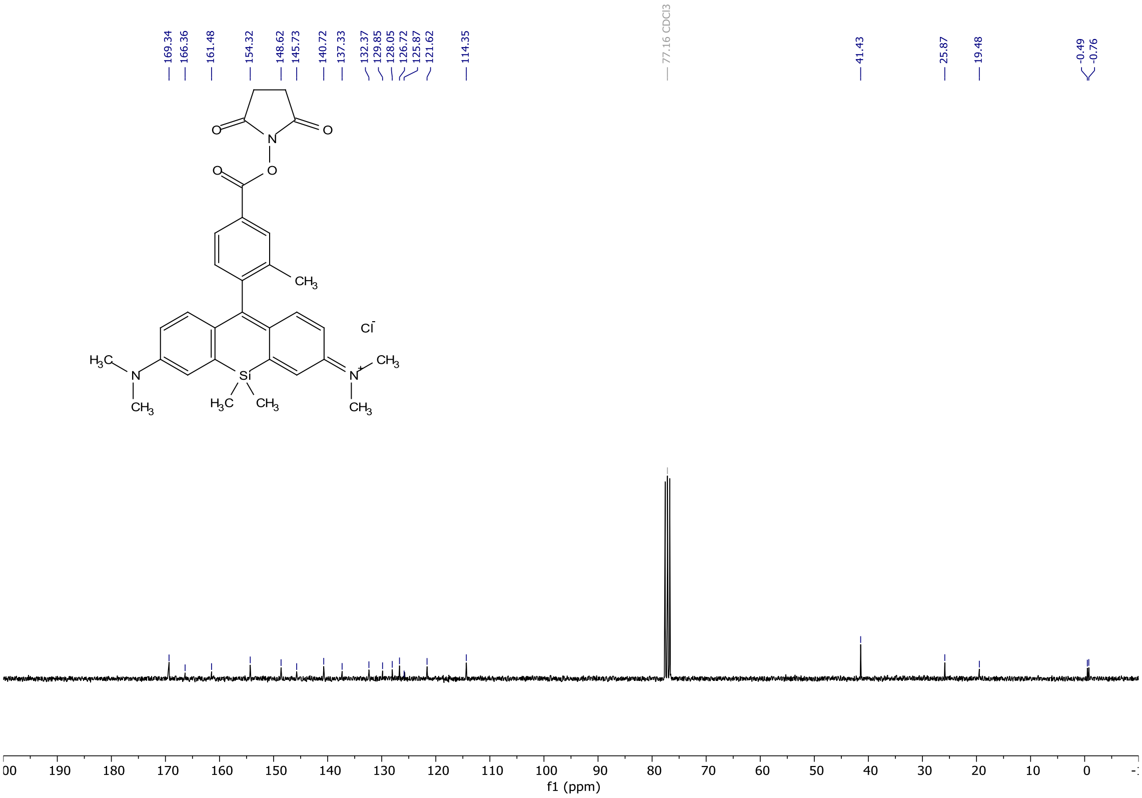
169.34
166.36
161.48
154.32
148.62
145.73
140.72
137.33
132.37
129.85
128.05
126.72
125.87
121.62
114.35
77.16 CDCl3
41.43
25.87
19.48
-0.49
-0.76
f1 (ppm)

2. Synthesis of biotin-NHS ester

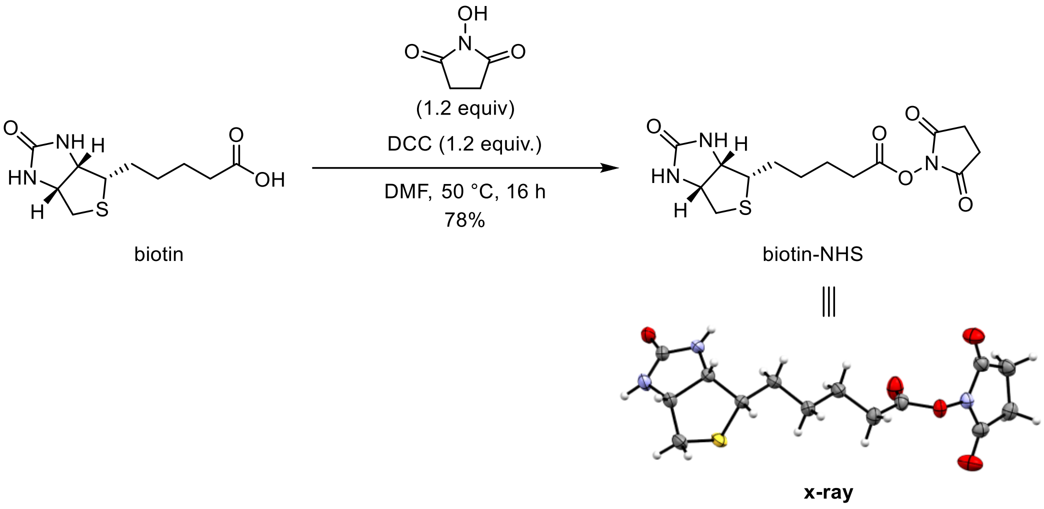

2,5-dioxopyrrolidin-1-yl-5-((3a*S*,4*S*,6a*R*)-2-oxohexahydro-1*H*-thieno[3,4-*d*]imidazol-4-yl)pentanoate (biotin-NHS). Adaptation of described procedure.[S6] To Biotin (1.148 g, 4.699 mmol), *N*-Hydroxysuccinimide (0.65 g, 0.565 mmol) and *N*,*N'*-Dicyclohexylcarbodiimide (1.163 g, 5.639 mmol) was added DMF (50 mL). The atmosphere in the flask was replaced with argon and the mixture heated to 50°C, giving a cloudy suspension. The mixture was filtered through a pad of celite. To the filtrate was added cold (-18°C) Et_2O, resulting in a precipitate. This precipitate was filtered off, washed thoroughly with cold Et_2O and the powder was dried under reduced pressure. This gave the desired compound in 78% yield (1.248 g, 3.654 mmol). A small sample was recrystallised from CH_2Cl_2 and MeOH to obtain single crystals of suitable quality for x-ray diffraction measurements (CCDC deposition number: 2177612). Colourless powder: **^{1}H NMR** (300 MHz, DMSO-*d*$_6$) δz 6.44 (s, 1H), 6.37 (s, 1H), 4.31 (dd, *J* = 7.7, 4.9 Hz, 1H), 4.15 (m, 1H), 3.11 (m, 1H), 2.85 (d, *J* = 5.1 Hz, 1H), 2.81 (s, 4H), 2.67 (t, *J* = 7.3 Hz, 2H), 2.58 (d, *J* = 12.4 Hz, 1H), 1.70 – 1.36 (m, 6H). **^{13}C NMR** (75 MHz, DMSO-*d*$_6$) δ 170.28, 168.95, 162.72, 61.01, 59.19, 55.24, 39.93, 30.01, 27.84, 27.59, 25.45, 24.31. **HRMS** (ESI) calculated for $[M+H]^+$ $C_{14}H_{20}N_3O_5S$ 342.1118, found 342.1113.

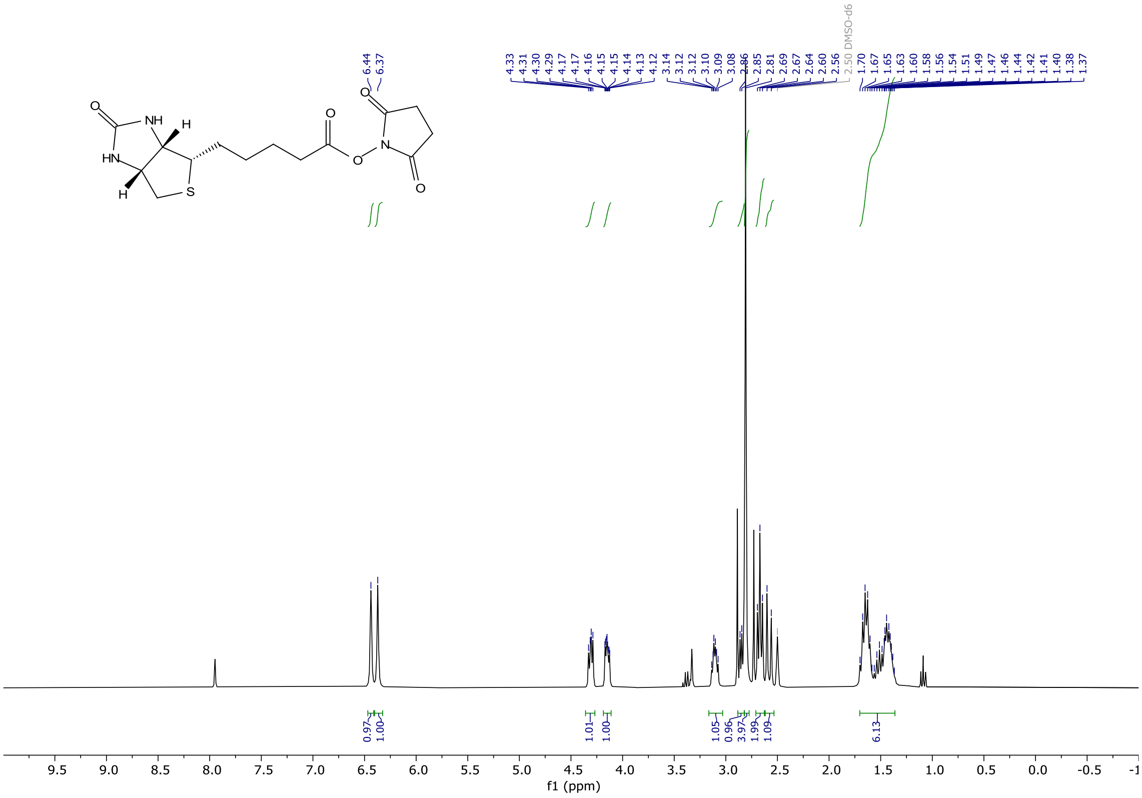

6.44
6.37
4.33
4.31
4.30
4.29
4.17
4.17
4.16
4.15
4.15
4.14
4.13
4.12
3.14
3.12
3.12
3.10
3.09
3.08
2.86
2.85
2.81
2.69
2.67
2.64
2.60
2.56
2.50 DMSO-d6
1.70
1.67
1.65
1.63
1.60
1.58
1.56
1.54
1.51
1.49
1.47
1.46
1.44
1.42
1.41
1.40
1.38
1.37
0.97
1.00
1.01
1.00
1.05
0.96
3.97
1.99
1.09
6.13
f1 (ppm)

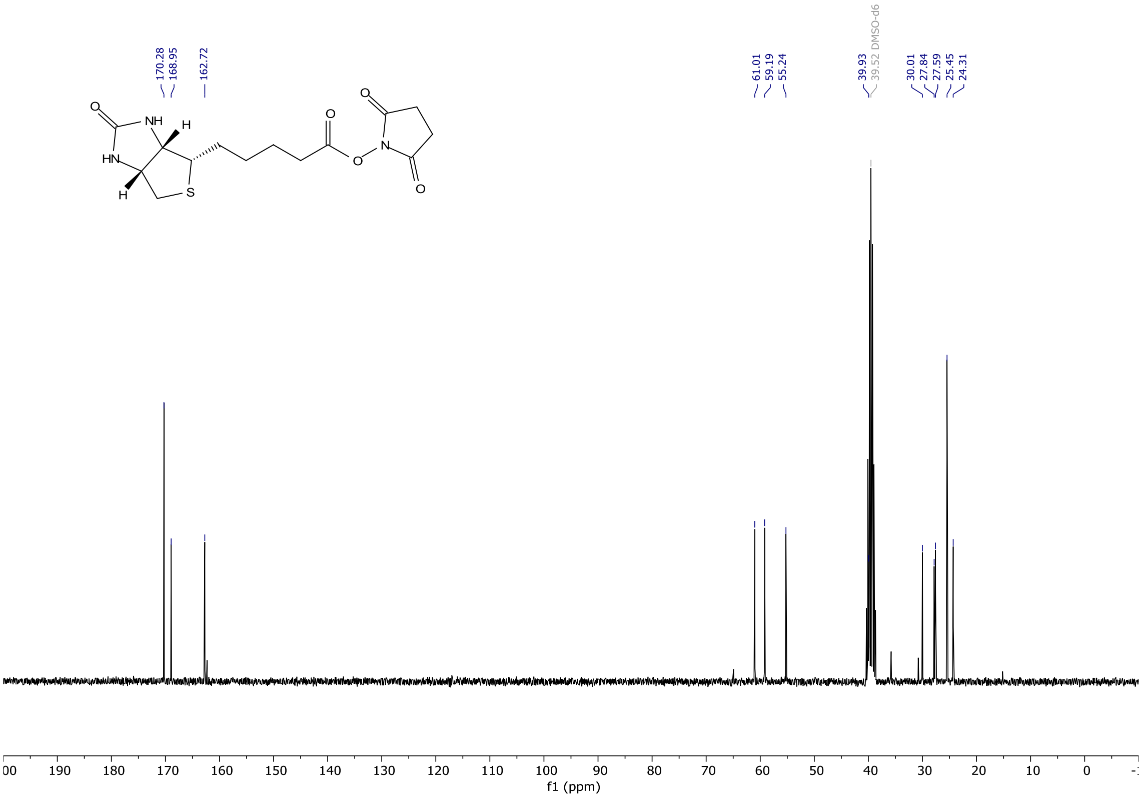

170.28
168.95
162.72
61.01
59.19
55.24
39.93
39.52 DMSO-d6
30.01
27.84
27.59
25.45
24.31
f1 (ppm)

3. Synthesis of azide linkers

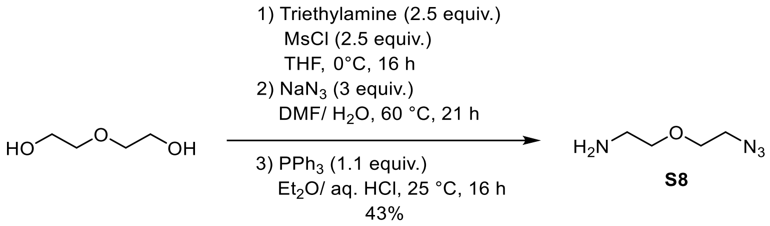

2-(2-azidoethoxy)ethan-1-amine (S8). According to modified literature procedure.[S7] To a stirred solution of diethylene glycol (5.00 g, 47.12 mmol) and methanesulfonyl chloride (9.2 mL, 13.616 g, 118.865 mmol) in THF (55 mL) at 0°C was added dropwise over the course of 25 min Et_3N (20 mL, 14.6 g, 144.283 mmol) in THF (30 mL). The mixture was slowly warmed to 23°C and stirred for 16 h. The suspension was filtered, and the filter cake was thoroughly washed with THF. The filtrate was concentrated under reduced pressure resulting in a clear orange oil. This oil was dissolved in a mixture of DMF (40 mL) and H_2O (30 mL). To this mixture sat. aq. $NaHCO_3$ (15 mL) and NaN_3 (6.404 g, 98.508 mmol) was added. The flask was protected from light and the mixture was stirred for 21 h at 60°C. The mixture was extracted with Et_2O (6 x 30 mL). The combined organic phases were washed with brine, dried over $MgSO_4$, filtered and concentrated under reduced pressure to ca. 30 mL residual volume of Et_2O . To this solution was added aq. HCl (1 M, 40 mL) and then dropwise over the course of 60 min PPh_3 (8.027 g, 31.531 mmol) in Et_2O (40 mL). The reaction mixture was left stirring at 25°C for 16 h. The organic phase was separated. The resulting aqueous phase was washed with Et_2O (6 x 10 mL). To the aqueous layer was added aq. NaOH (20%, 10 mL). This aqueous mixture was extracted with CH_2Cl_2 (5 x 25 mL). The combined organic layers were washed with brine, dried over $MgSO_4$, filtered and concentrated under reduced pressure. This gave the desired compound in 43% yield (2.966 g, 20.430 mmol). Clear yellowish oil: **^{1}H NMR** (300 MHz, $CDCl_3$) δ 3.63 (t, *J* = 5.0 Hz, 2H), 3.50 (t, *J* = 5.1 Hz, 2H), 3.36 (t, *J* = 4.9 Hz, 2H), 2.88 – 2.83 (t, *J* = 5.1 Hz, 2H), 1.76 (s, 2H). **^{13}C NMR** (75 MHz, $CDCl_3$) δ 73.18, 70.05, 50.84, 41.79. **HRMS** (ESI) calculated for $[M+H]^+$ $C_4H_{11}N_4O^+$ 131.0927, found 131.0923.

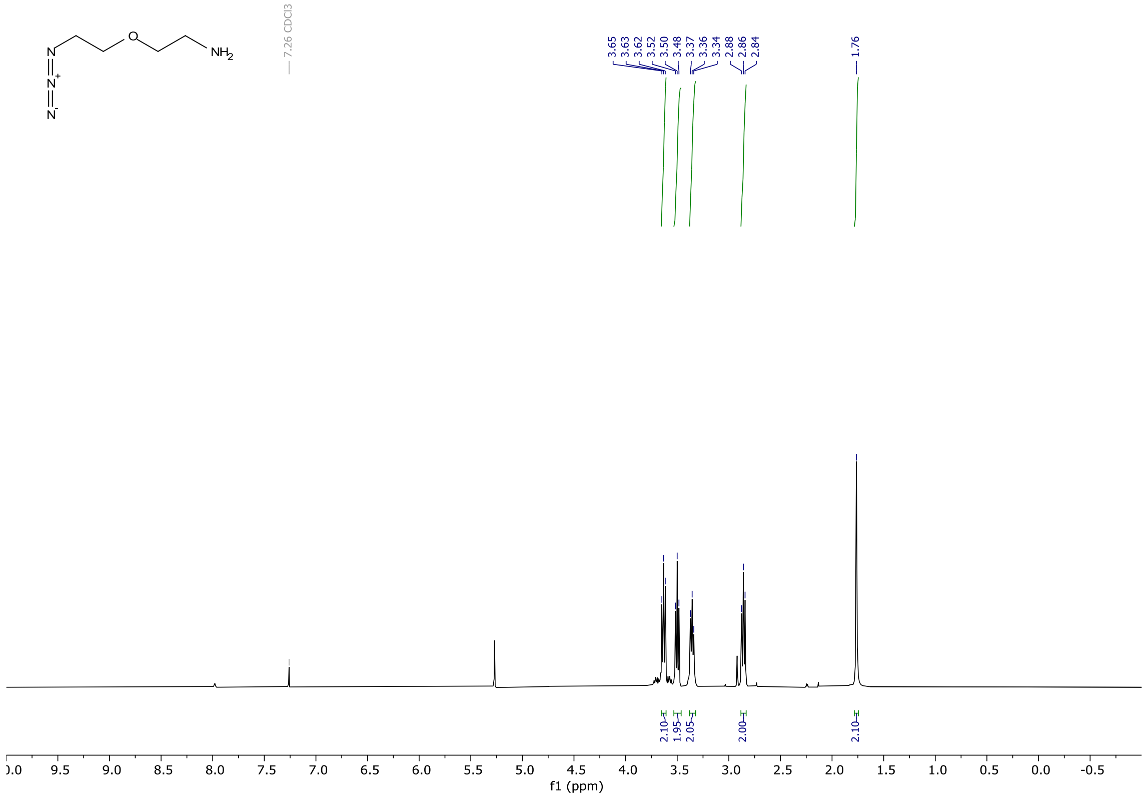

NH2
7.26 CDCl3
3.65
3.63
3.62
3.52
3.50
3.48
3.37
3.36
3.34
2.88
2.86
2.84
1.76
2.10
1.95
2.05
2.00
2.10
f1 (ppm)

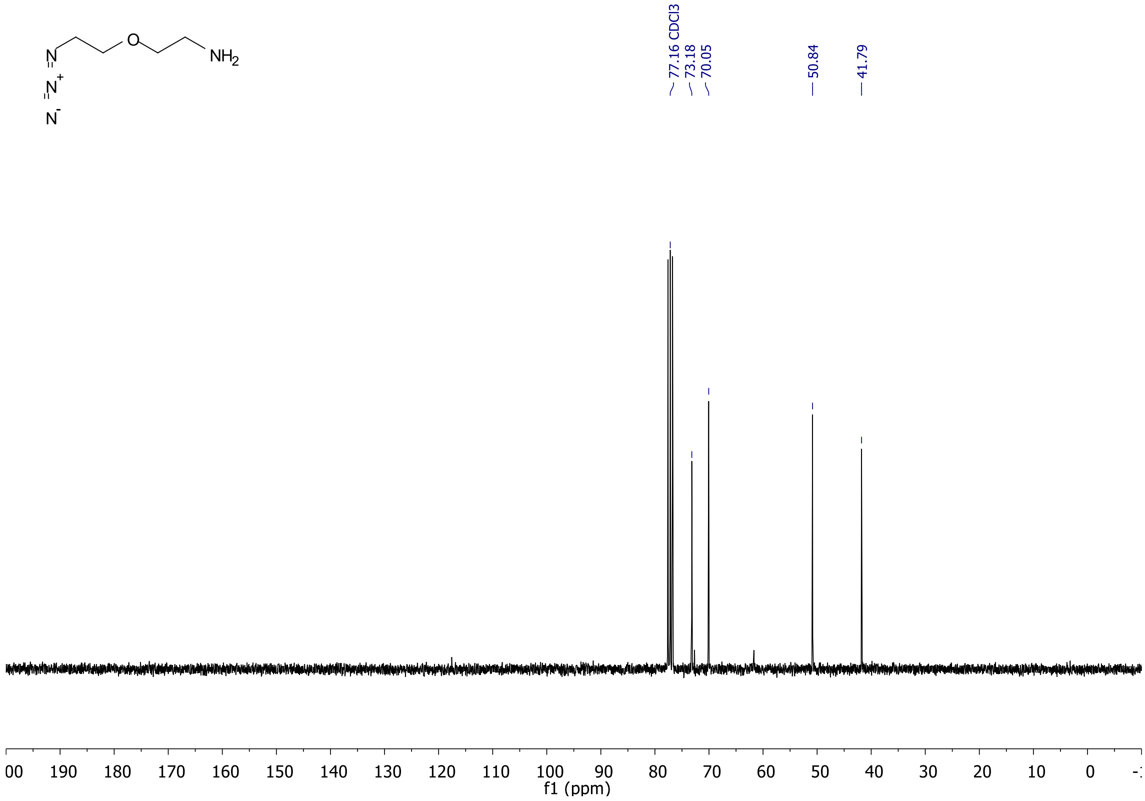

NH2
77.16 CDCl3
73.18
70.05
50.84
41.79
f1 (ppm)

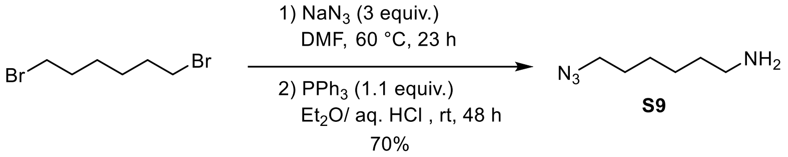

6-azidohexan-1-amine (S9). According to modified literature procedure.[S7] To a stirred solution of 1,6-dibromohexane (4.8 mL, 7.61 g, 31.2 mmol) in dry DMF (80 mL) was added NaN_3 (6.07 g, 93.4 mmol). The mixture was heated to 60°C and stirred for 23 h under an argon atmosphere. To the reaction mixture water (150 mL) was added and the mixture was extracted with Et_2O (4 x 20 mL). The combined organic phases were dried over $MgSO_4$ and filtered into a round bottom flask. To this flask was added aqueous HCl (1 M, 30 mL), EtOAc (60 mL) and PPh_3 (9.03 g, 34.42 mmol) in portions. First a slight gas evolution was observed, which ceased with the later additions. The mixture was stirred for 48 h under an argon atmosphere. The mixture was diluted with aq. HCl (1 M, 30 mL) and extracted with CH_2Cl_2. The organic layers were discarded and the aqueous layer was basified with aq. NaOH and extracted with CH_2Cl_2. The combined organic layers were washed with brine, dried over $MgSO_4$, filtered, and concentrated under reduced pressure to give the desired compound in 70% yield (3.109 g, 21.865 mmol). Slight yellow oil: **^{1}H NMR** (300 MHz, $CDCl_3$) δ 3.24 (t, *J* = 6.9 Hz, 2H), 2.67 (t, *J* = 6.8 Hz, 2H), 1.59 (p, *J* = 6.8 Hz, 2H), 1.50 – 1.20 (m, 8H). **^{13}C NMR** (75 MHz, $CDCl_3$) δ 51.49, 42.16, 33.65, 28.91, 26.68, 26.53. **HRMS** (ESI) calculated for $[M+Na]^+$ $C_6H_{15}N_4$ 143.1291, found 143.1291.

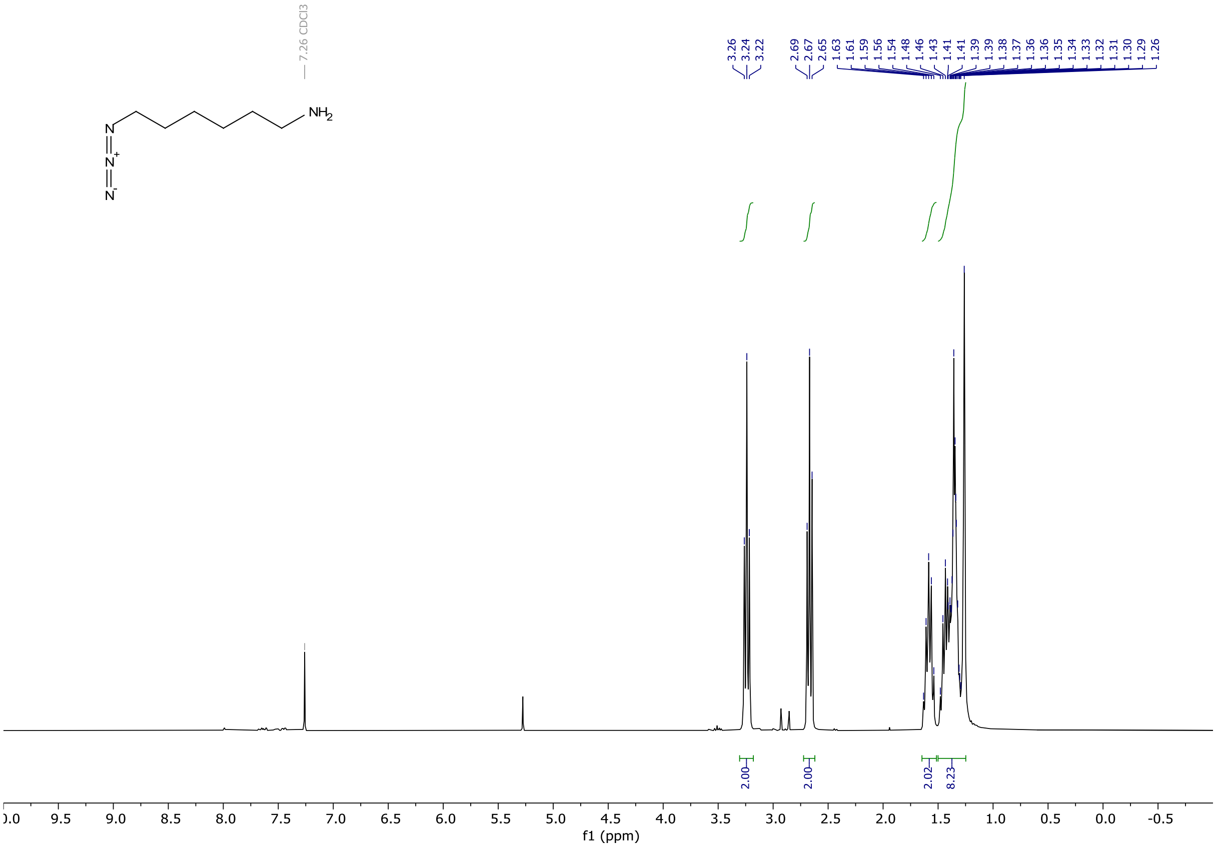

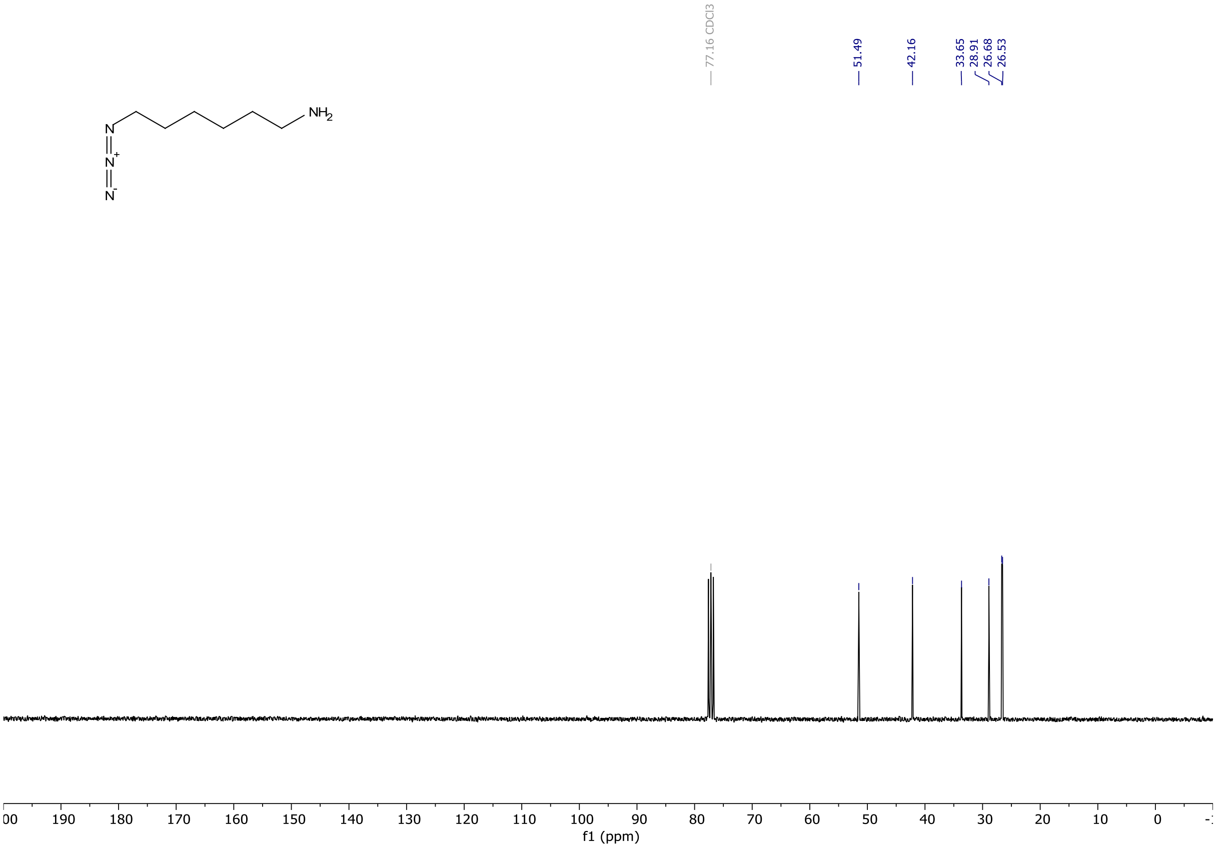

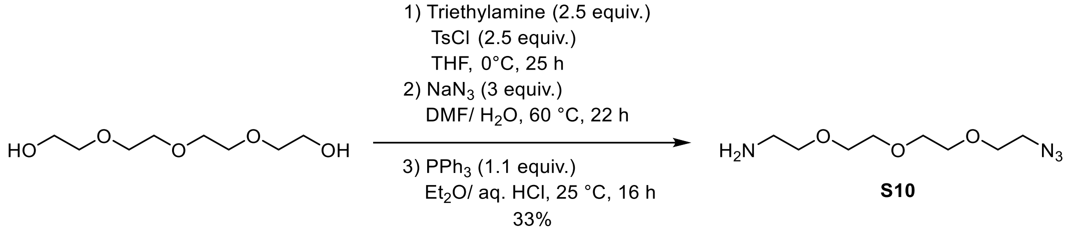

2-(2-(2-(2-azidoethoxy)ethoxy)ethoxy)ethan-1-amine (S10). According to modified literature procedure.[S7] To a stirred solution of tetraethylene glycol (4.746 g, 24.435 mmol) in THF (50 mL) at 0°C was added TsCl (11.622 g, 60.96 mmol). The mixture was stirred for 5 min. Then a solution of Et_3N (8.5 mL, 6.205 g, 61.320 mmol) in THF (30 mL) was added dropwise over the course of 15 min. The mixture was slowly warmed to room temperature and stirred for 25 h. The mixture was filtered through a por3 filter and the filter cake was thoroughly washed with 100 mL THF. The clear colourless filtrate was concentrated under reduced pressure. The crude bis-tosylate was dissolved in a mixture of DMF (50 mL) and H_2O (30 mL) resulting in a suspension. To this was added sat. aq. $NaHCO_3$ (20 mL) followed by careful addition of NaN_3 (4.737 g, 72.865 mmol). The slight turbid orange mixture was heated to 80°C and stirred for 22 h. After cooling, the mixture was extracted with Et_2O. The organic phase was completely colourless. The combined organic phases were washed with brine, and the yellow aqueous phase was discarded. The organic phase was dried over $MgSO_4$, filtered and concentrated under reduced pressure, which gave a clear colourless oil. This oil was dissolved in a mixture of Et_2O (40 mL) and 1 M HCl (80 mL), cooled to 0°C and placed under an argon atmosphere. To this mixture was added slowly PPh_3 (4.352 g, 16.593 mmol) in Et_2O (40 mL) over the course of 1 h. The mixture was slowly warmed to 21°C and stirred for 16 h. The organic phase was separated and the aqueous phase was washed with Et_2O (5 x). The organic wash phases were discarded. The aqueous layer was cooled to

0°C and basified with 2 M aq. NaOH. The aqueous phase was then extracted with CH_2Cl_2, the combined organic layers were washed with brine, dried over $MgSO_4$, filtered and concentrated under reduced pressure to give the desired compound in 33% yield (1.731 g, 7.931 mmol). Yellowish oil: **^{1}H NMR** (300 MHz, $CDCl_3$) δ 3.69 – 3.63 (m, 10H), 3.51 (t, *J* = 5.2 Hz, 2H), 3.38 (t, *J* = 5.1 Hz, 2H), 2.86 (t, *J* = 5.2 Hz, 2H), 1.66 (s, 2H). **^{13}C NMR** (75 MHz, $CDCl_3$) δ 73.42, 70.85, 70.80, 70.78, 70.42, 70.18, 50.83, 41.88. **HRMS** (ESI) calculated for $[M+H]^+$ $C_8H_{19}N_4O_3$ 219.1452, found 219.1450.

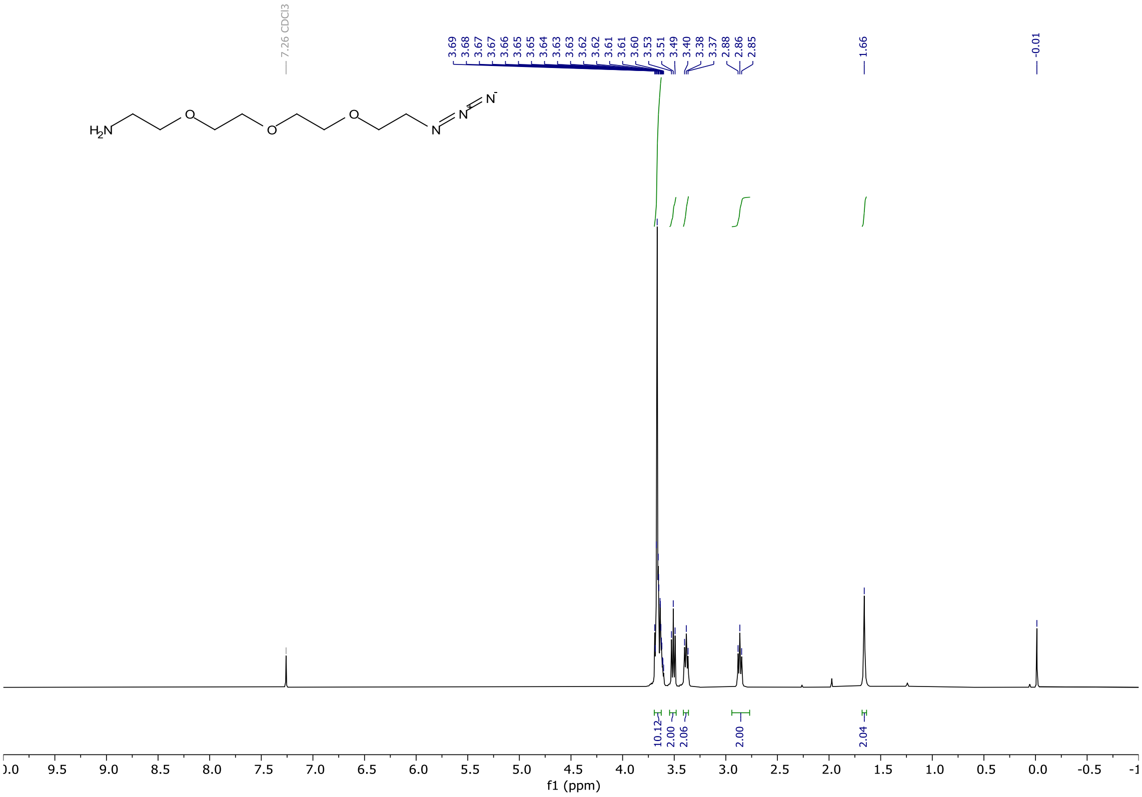

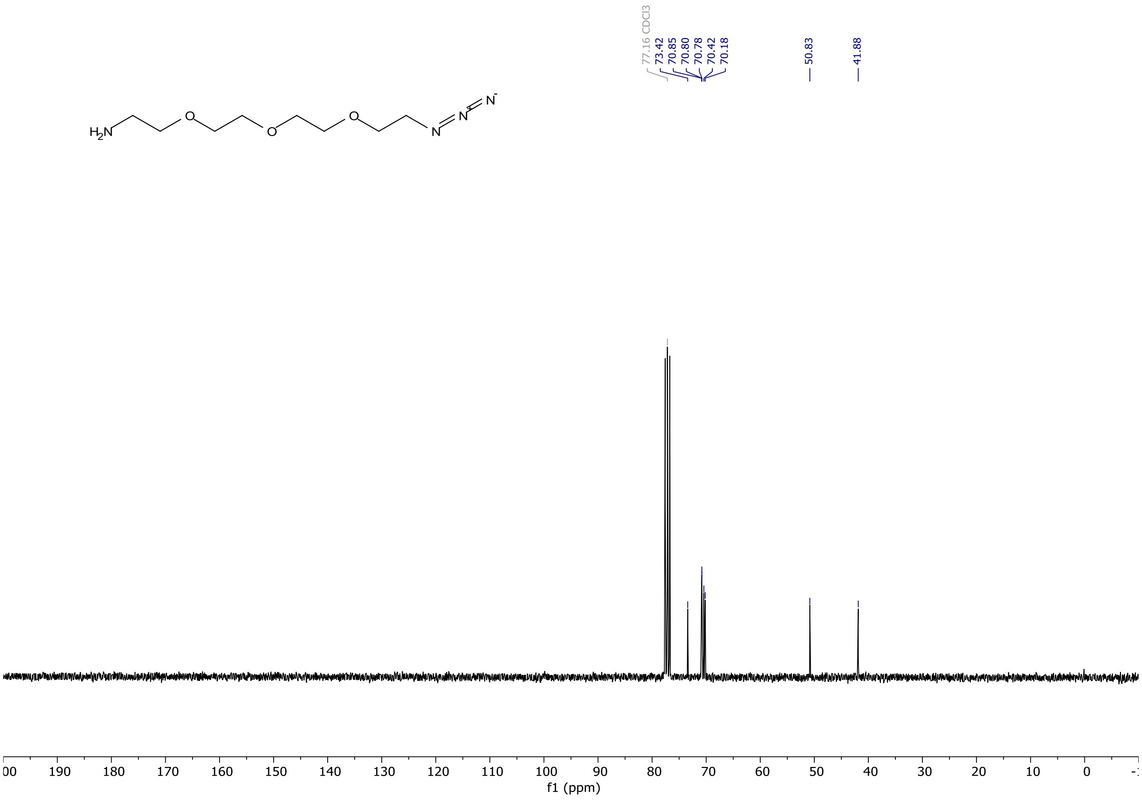

4. Observed side reactions with NHS-activated esters

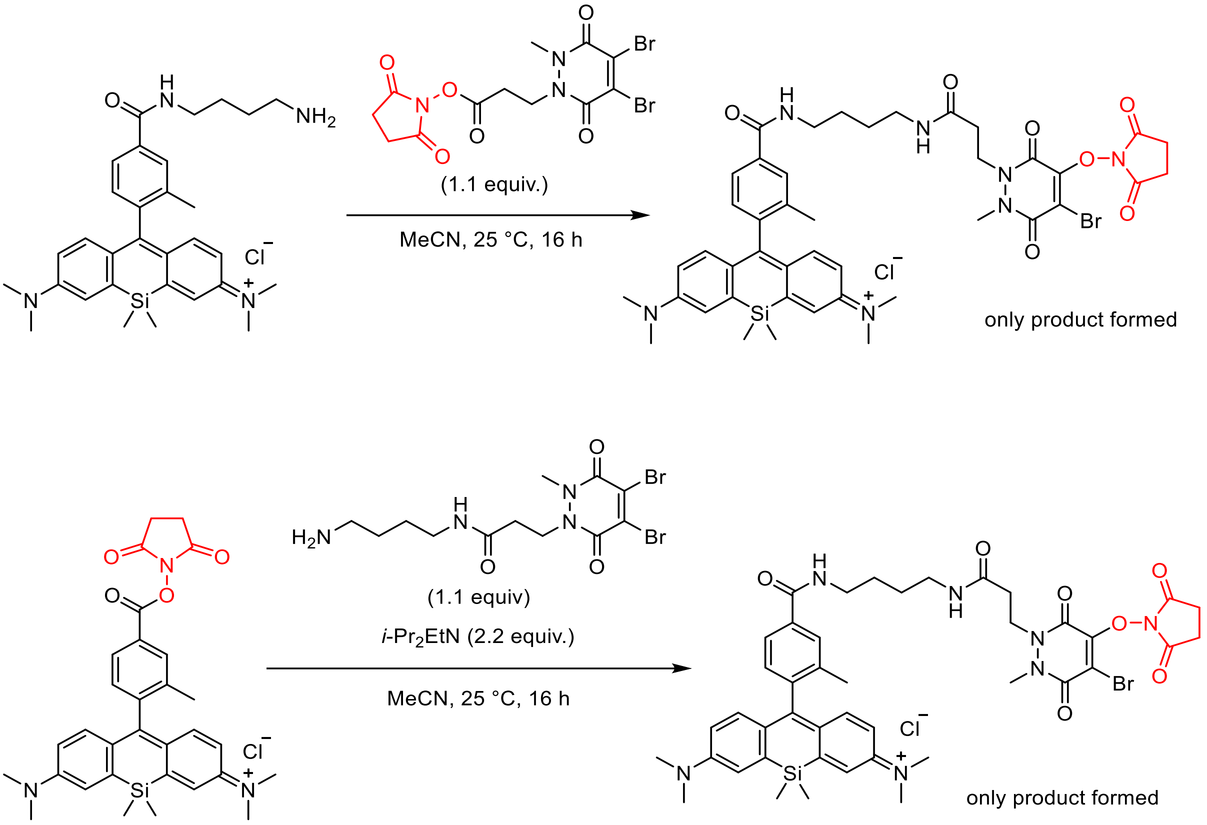

Scheme S2 Original coupling strategy to link fluorescent SiR dye with dibromopyridazinediones via NHS-activated esters. Even though the amide bond formation was very efficient, one bromine atom of the dibromopyridazinediones was displaced by the *N*-hydroxysuccinimid leaving group, leading to the shown products. The isolated adducts are most probably a mixture of regioisomers and their NMR and HRMS spectra are in agreement with the depicted structures. HRMS (ESI) calculated for $[M\text{-}Cl]^+$ $C_{43}H_{51}BrN_7O_7Si$ 884.2803, found 884.2825.

5. SDS-PAGE of modified anti GAPDH antibody under non-reducing conditions

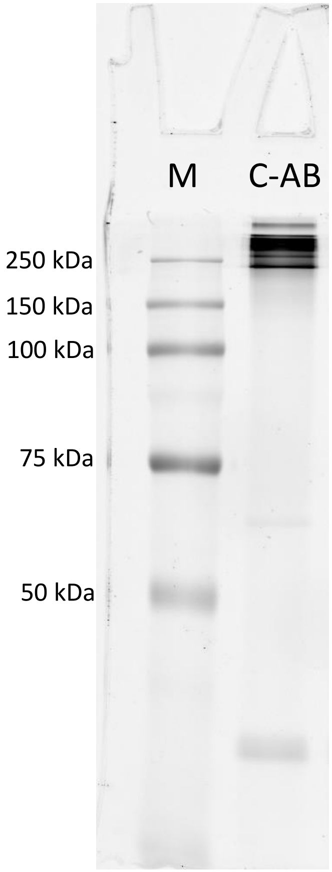

Figure S1 SDS-PAGE of anti GAPDH antibody after re-bridging with SiR-labelled dibromopyridazinedione **8** (lane C-AB) using non-reducing loading buffer (not containing mercaptoethanol). Detection of protein bands by fluorescence (Cy5 channel). Several fluorescent bands at around 250 kDa most probably result from aggregated and incompletely linearised labelled antibodies. Compare to SDS-PAGE of same antibody sample using reducing loading buffer (Figure 1B). Lanes: M, All Blue protein standard; C-AB, SiR-conjugated anti GAPDH antibody.

6. Size exclusion chromatography (SEC) of BFL-modified anti GAPDH and native anti GAPDH antibodies

BFL-modified anti GAPDH antibody **native anti GAPDH antibody**

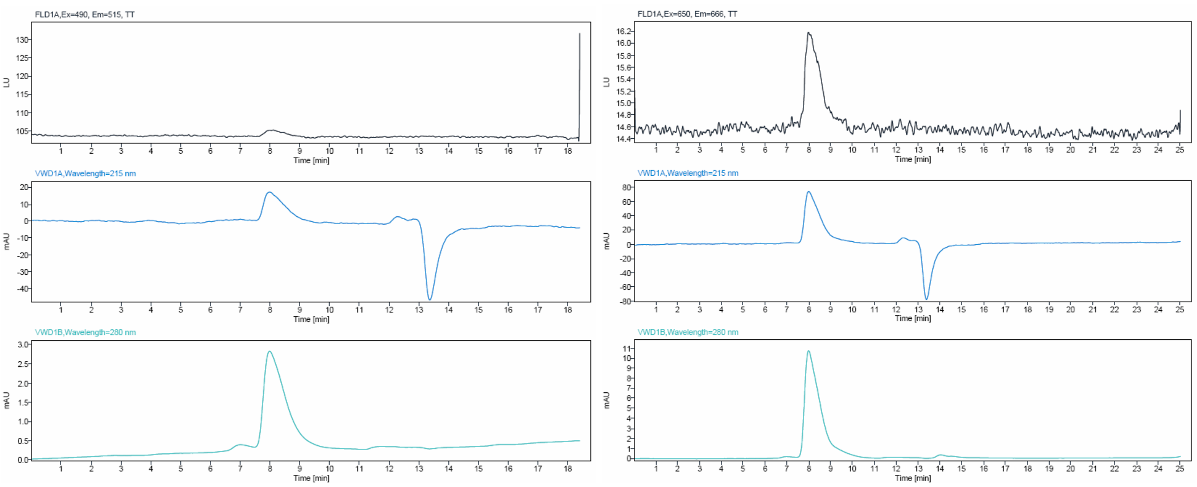

Figure S2 Size exclusion chromatograms of BFL-modified anti GAPDH antibody after re-bridging with dibromopyridazinedione **9** (left traces) and of native anti GAPDH antibody (right traces). Detection of antibodies by fluorescence (top traces, note the different scale of the intensity axes), by absorption at 215 nm (middle traces) and at 280 nm (bottom traces).

7. Direct conjugation of anti GAPDH antibody

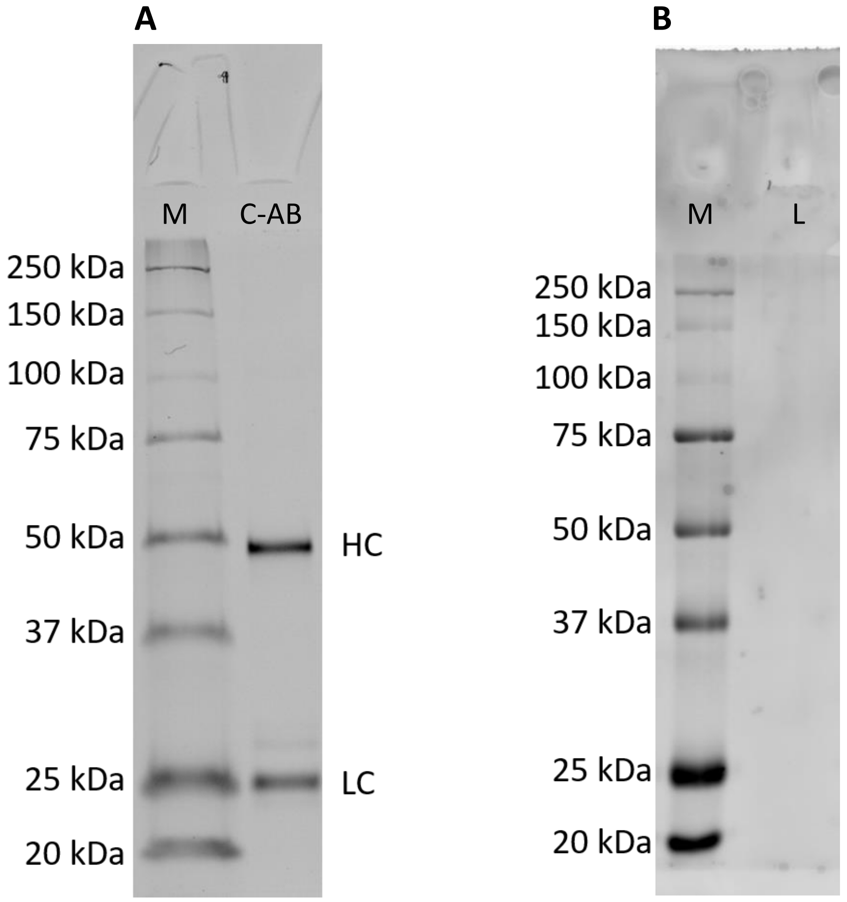

Figure S3 Anti GAPDH antibody was directly labelled with electrophilic, fluorescent reagent SiR-NHS (detailed procedure see *Experimental section*). (**A**) SDS-PAGE of anti GAPDH antibody conjugation product (lane C-AB) detected by fluorescence (excitation at 635 nm, Cy5 channel). Bands at 50 kDa and 25 kDa correspond to the heavy (HC) and light chains (LC), respectively. (**B**) Western blot analysis of mouse cardiomyocyte whole cell lysate (lane L) using directly SiR-labelled anti GAPDH antibody from (**A**). Detection by fluorescence (Cy5 channel) did not show any protein band at 36 kDa corresponding to mouse GAPDH. M, All Blue Protein Standard.

8. SDS-PAGE of IgG antibody conjugates

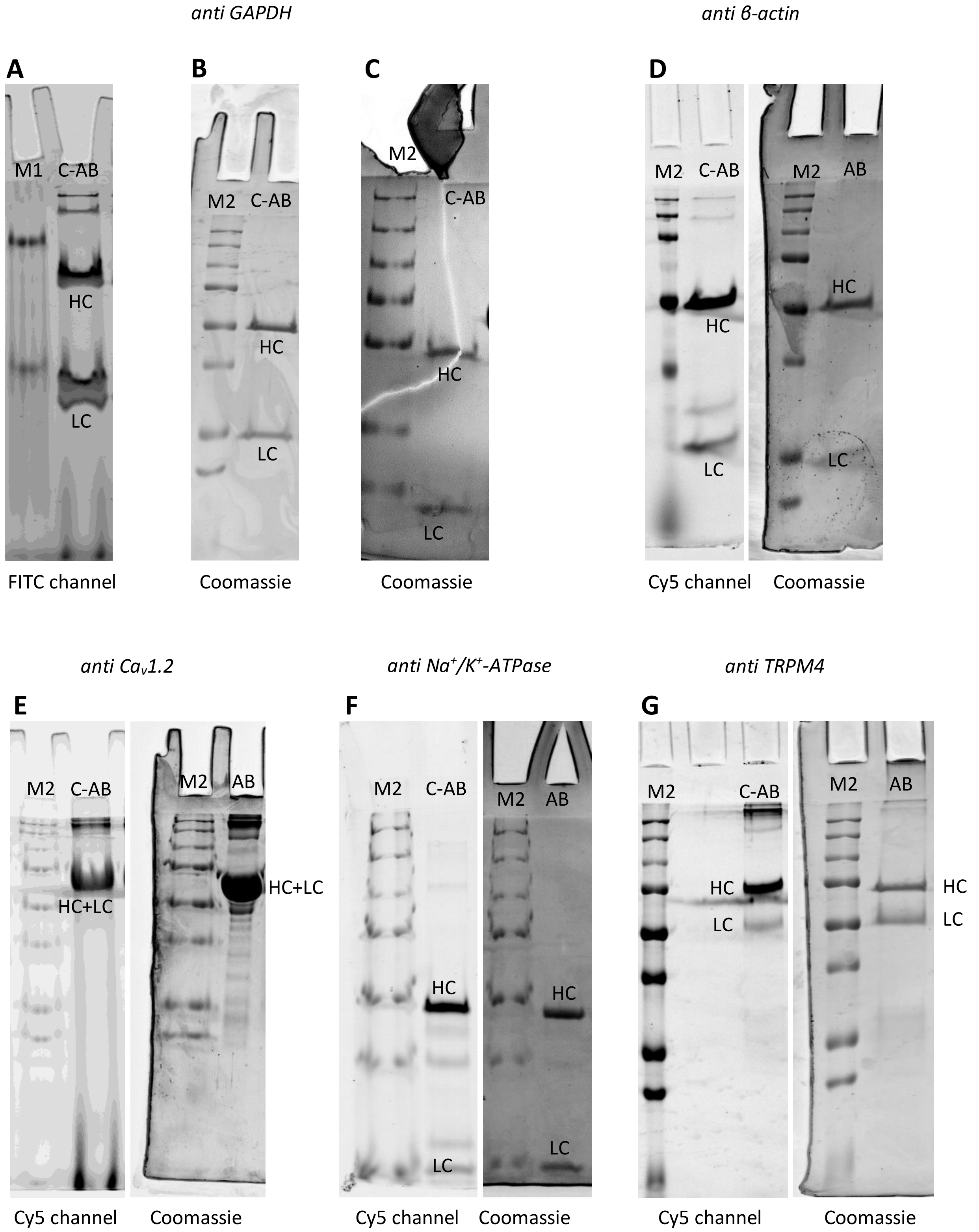

Figure S4

anti Na_v1.5

H

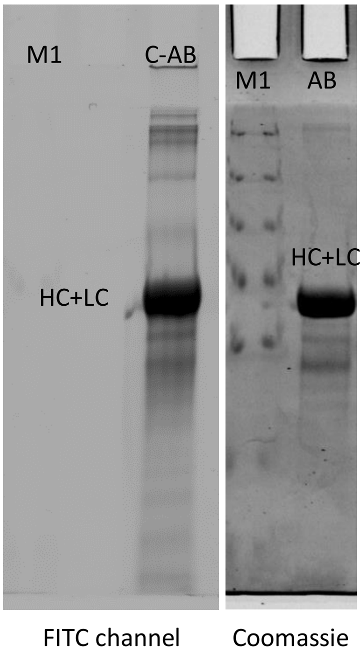

Figure S4 (*Continued*) (**A**) BFL-conjugated anti GAPDH antibody. (**B**) Biotin-conjugated anti GAPDH antibody. (**C**) PB-conjugated anti GAPDH antibody. (**D**) Left gel: SiR-conjugated anti β-actin antibody. Right gel: native anti β-actin antibody. (**E**) Left gel: SiR-conjugated anti Ca_v1.2 antibody. Right gel: native anti Ca_v1.2 antibody. (**F**) Left gel: SiR-conjugated anti Na^+/K^+-ATPase antibody. Right gel: native anti Na^+/K^+-ATPase antibody. (**G**) Left gel: SiR-conjugated anti TRPM4 antibody. Right gel: native anti TRPM4 antibody. (**H**) Left gel: BFL-conjugated anti Na_v1.5 antibody. Right gel: native anti Na_v1.5 antibody. For native GAPDH antibody, see Figure 1C. Lanes: M1, Protein Standard (Thermo Fisher); M2, All Blue Protein Standard (Bio-Rad); AB, native antibody; C-AB, conjugated antibody. HC, heavy chains; LC, light chains; HC+LC, one heavy and one light chain.

9. Western blot and direct immunofluorescence with IgG antibody conjugates

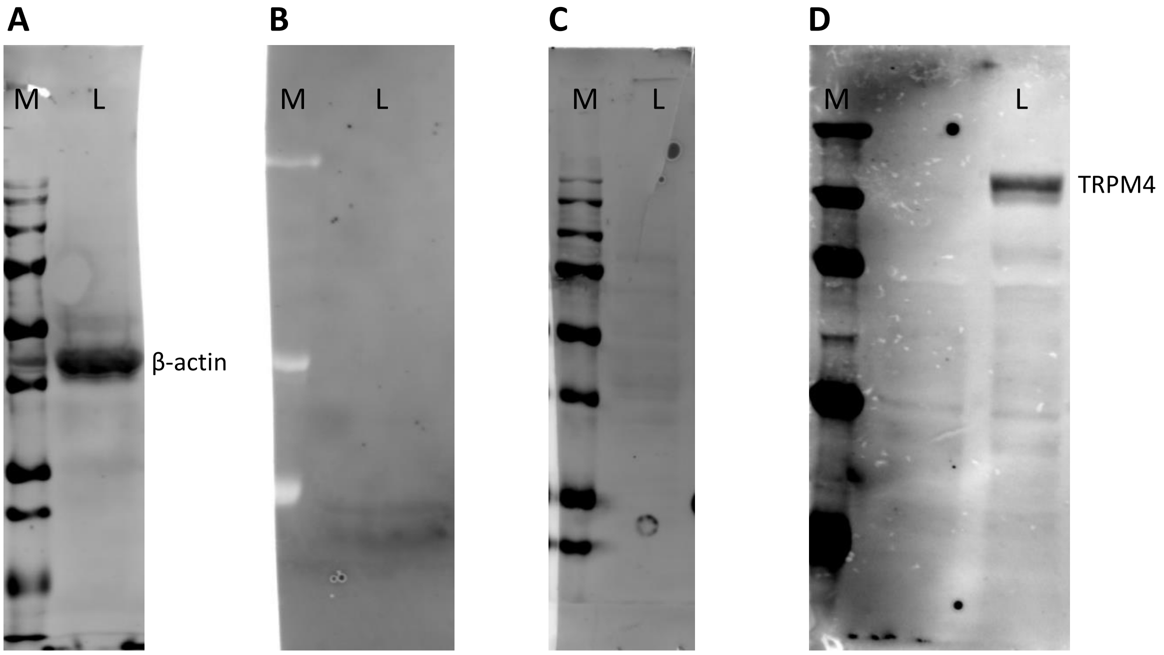

Figure S5 Western blot analysis of mouse cardiomyocyte whole cell lysate using (**A**) SiR-modified anti β-actin antibody, (**B**) BFL-modified anti $Na_v1.5$ antibody, (**C**) SiR-modified anti Na^+/K^+-ATPase antibody or (**D**) SiR-modified anti TRPM4 antibody. SiR- and BFL-conjugated antibodies were detected directly by fluorescence (Cy5 and FITC channel, respectively). Lanes: M, Marker, L, Lysate.

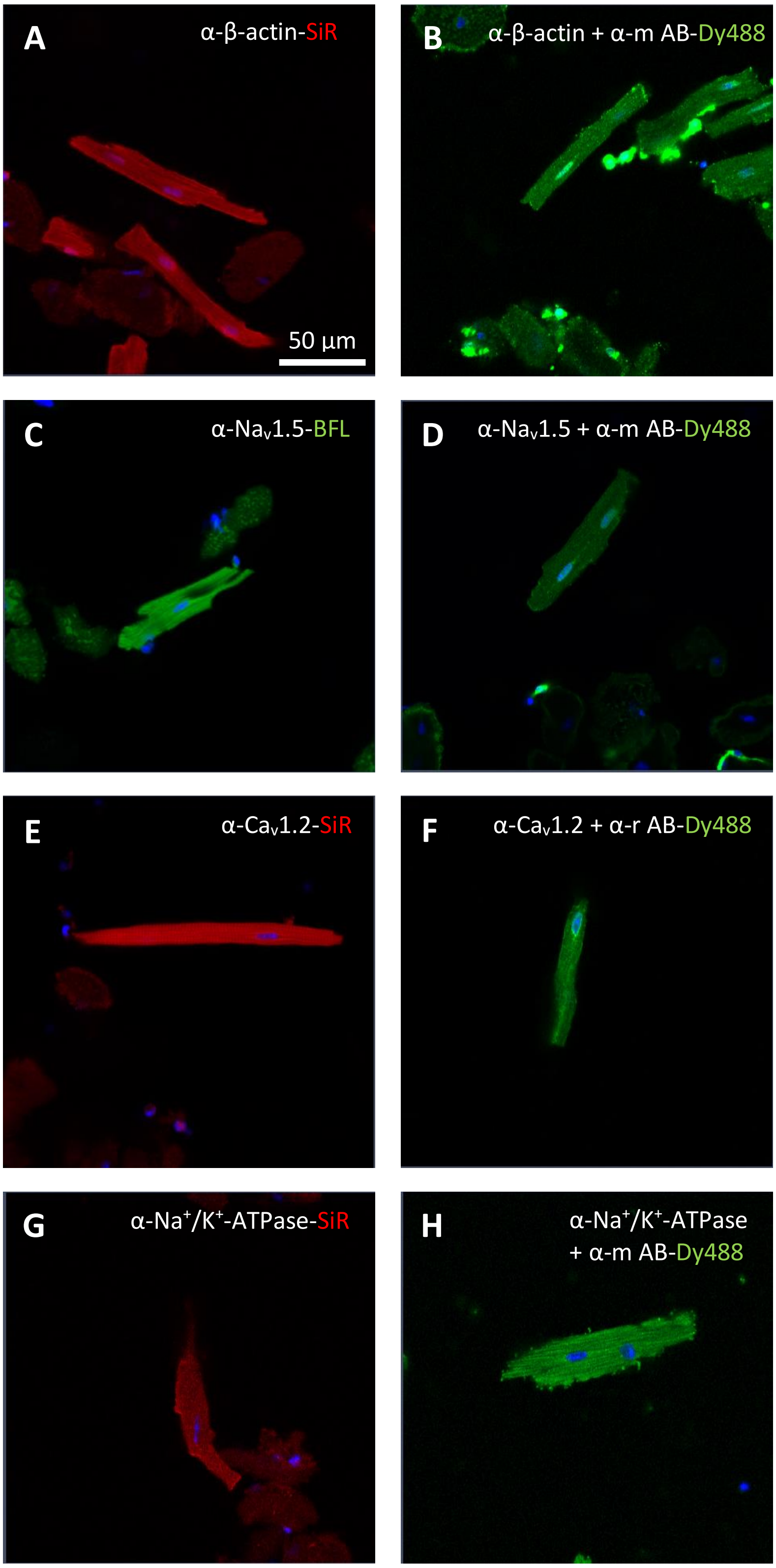
A
α-β-actin-SiR
50 µm
B
α-β-actin + α-m AB-Dy488
C
α-Nav1.5-BFL
D
α-Nav1.5 + α-m AB-Dy488
E
α-Cav1.2-SiR
F
α-Cav1.2 + α-r AB-Dy488
G
α-Na+/K+-ATPase-SiR
H
α-Na+/K+-ATPase
+ α-m AB-Dy488

Figure S6

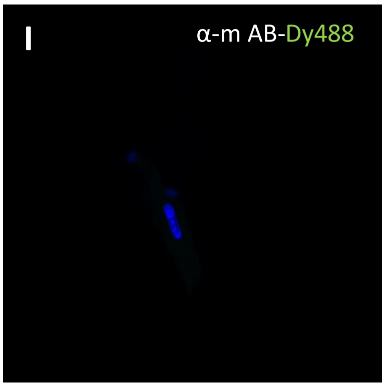

Figure S6 (*Continued*) Direct (**A**, **C**, **E**, **G**) and indirect (**B**, **D**, **F**, **H**) immunofluorescence with isolated and fixed mouse cardiomyocytes using conjugated and native antibodies. (**A**) SiR-modified anti β-actin antibody. (**B**) Native anti β-actin antibody. (**C**) BFL-modified anti $Na_v1.5$ antibody. (**D**) Native anti $Na_v1.5$ antibody. (**E**) SiR-modified anti $Ca_v1.2$ antibody. (**F**) Native anti $Ca_v1.2$ antibody. (**G**) SiR-modified anti Na^+/K^+-ATPase antibody. (**H**) Native anti Na^+/K^+-ATPase antibody. Detection of primary antibodies by fluorescence (**A**, **C**, **E**, **G**; Cy5 or FITC channel, respectively) or by secondary goat anti mouse or anti rabbit Dy488-labelled antibodies (**B**, **D**, **F**, **H**). (**I**) Control experiment: mouse cardiomyocytes incubated with secondary goat anti mouse Dy488-laballed antibody alone (Cy5, FITC and DAPI channels overlay). Cell nuclei stained with DAPI (blue). SiR, Si-rhodamine; BFL, BODIPY FL; Dy488, DyLight® 488; α-m AB, goat anti mouse antibody; α-r AB, goat anti rabbit antibody. Scale bar represents 50 µm.

10. Absorption and emission spectra for fluorescent dibromopyridazinediones 8-10

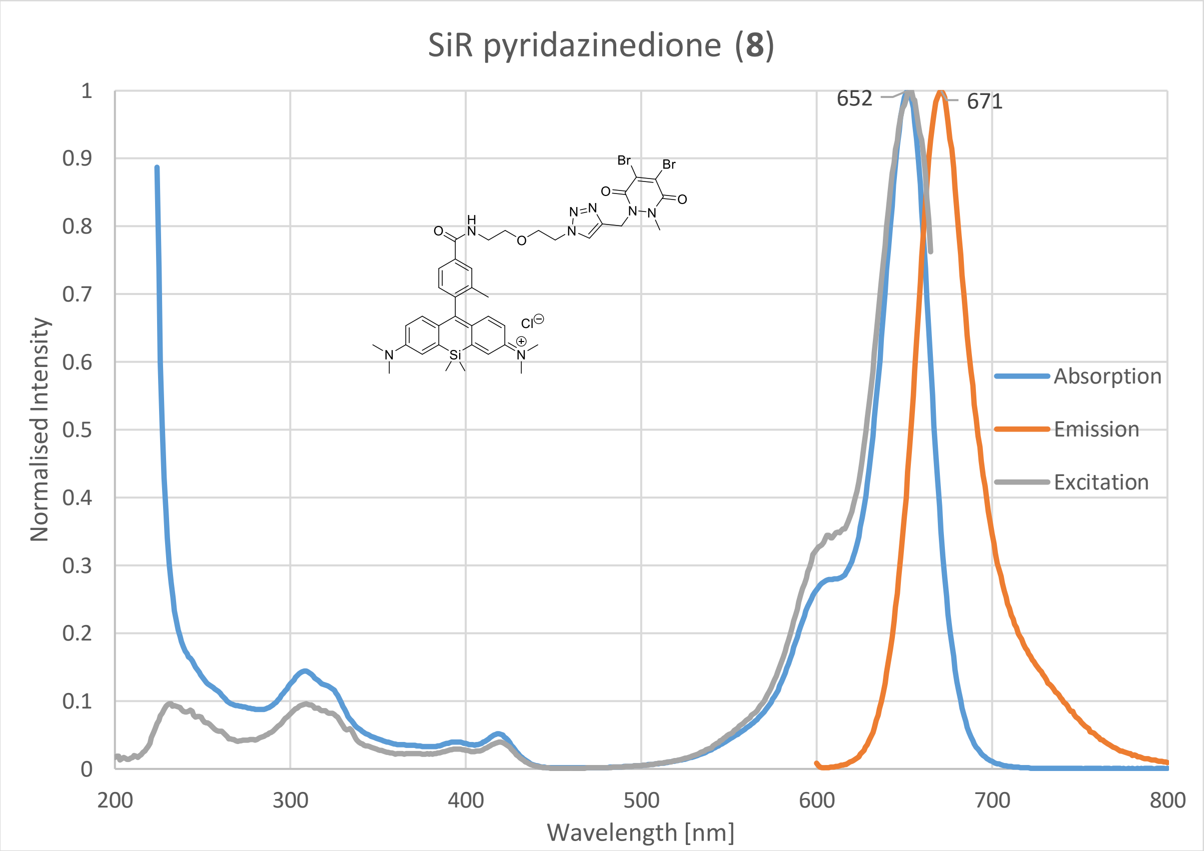

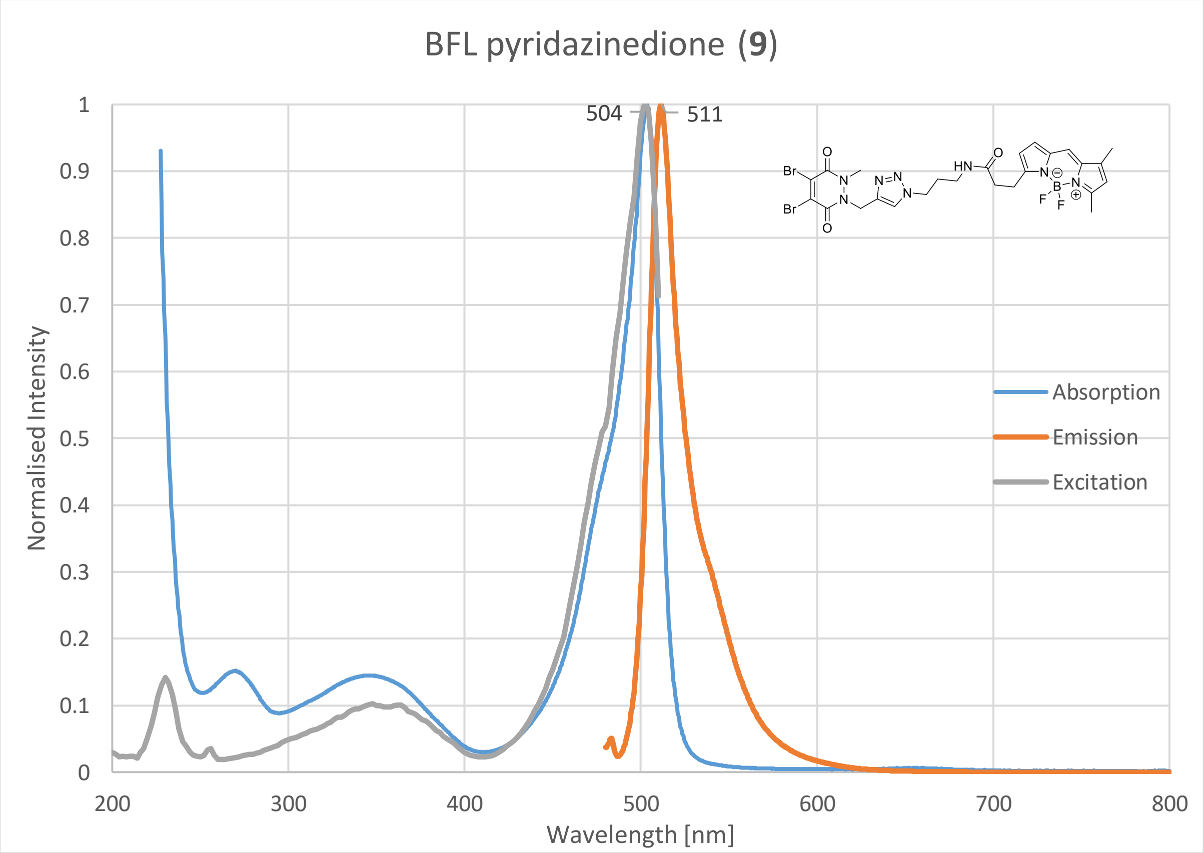

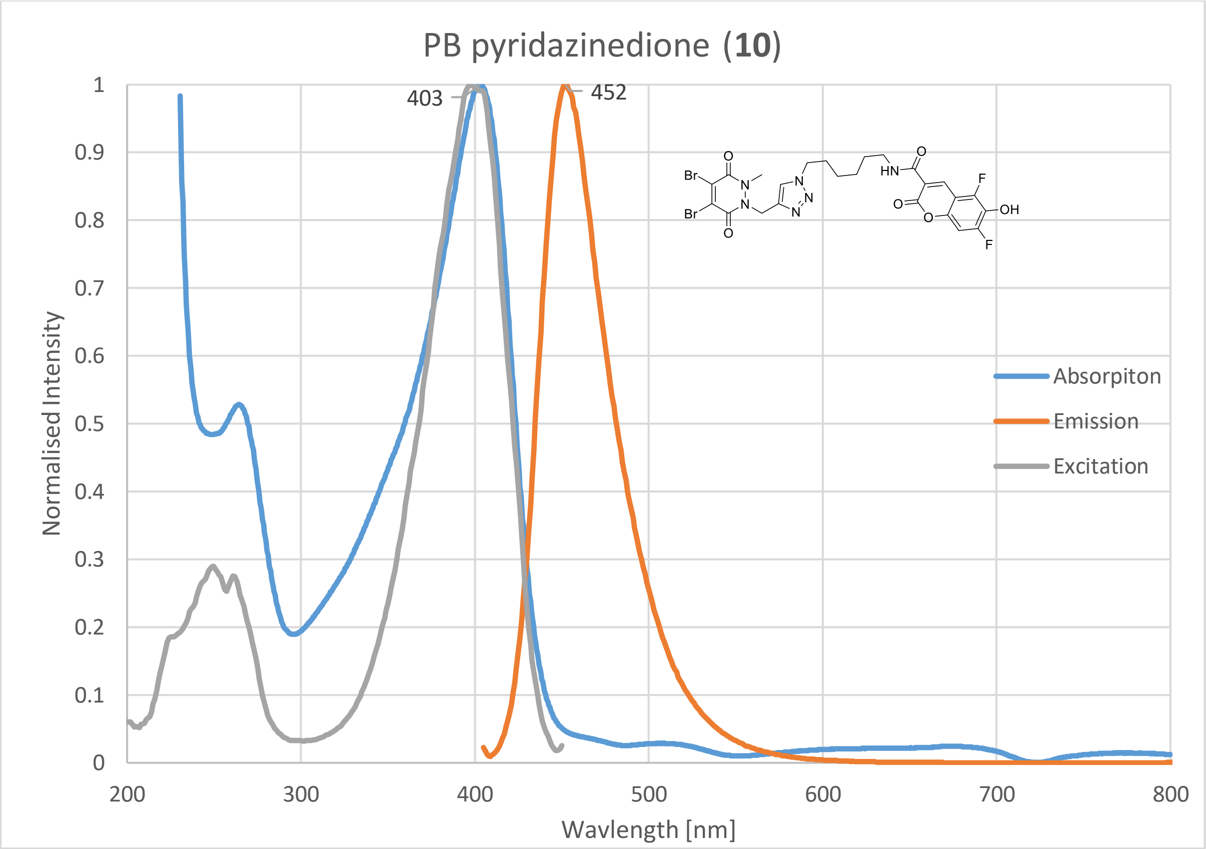

PB pyridazinedione (**10**)
403
452
Normalised Intensity
Wavlength [nm]
Absorpiton
Emission
Excitation
0
0.1
0.2
0.3
0.4
0.5
0.6
0.7
0.8
0.9
1
200
300
400
500
600
700
800

11. NMR spectra for final compounds

Di-*tert*-butyl 1-methylhydrazine-1,2-dicarboxylate (1)

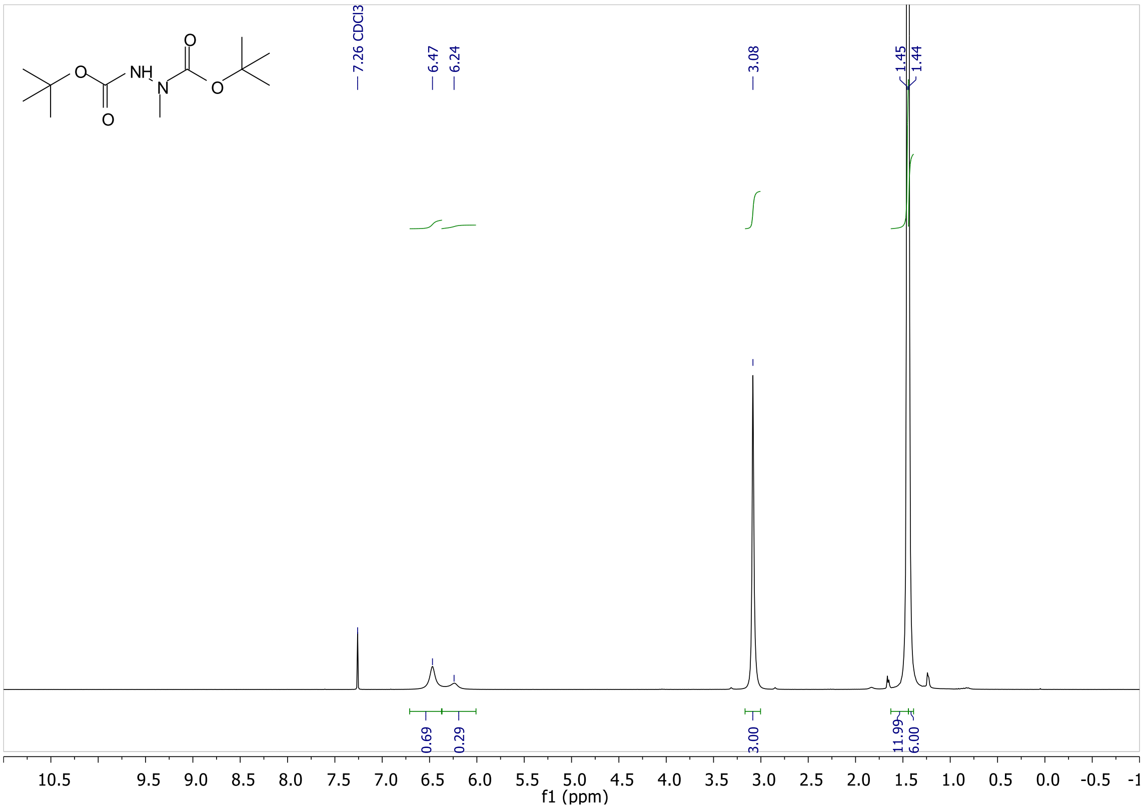

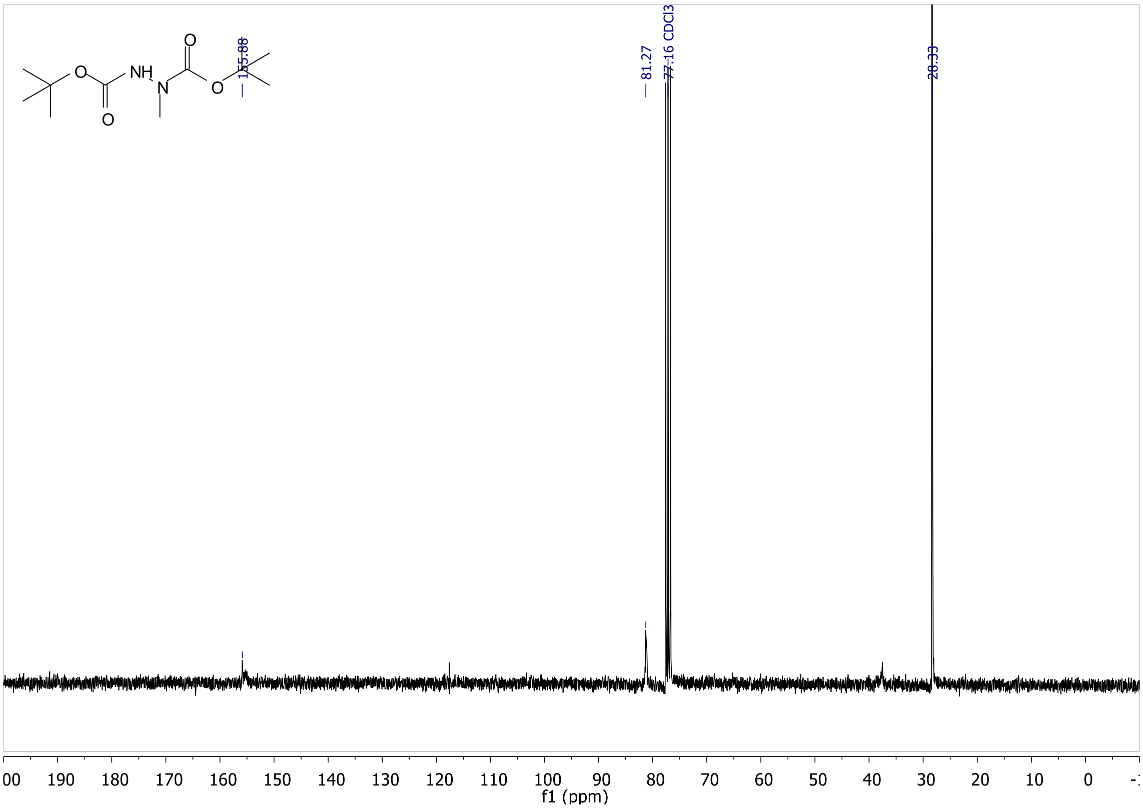

Di-*tert*-butyl 1-methyl-2-(prop-2-yn-1-yl)hydrazine-1,2-dicarboxylate (2)

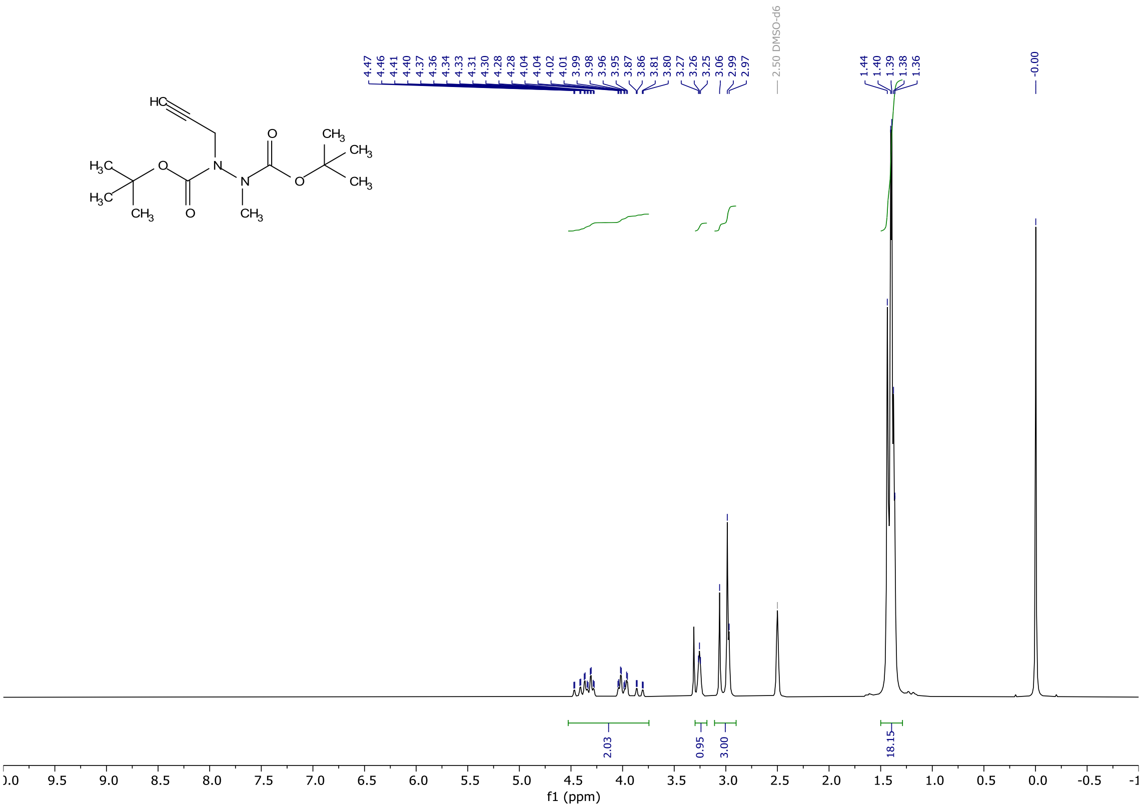

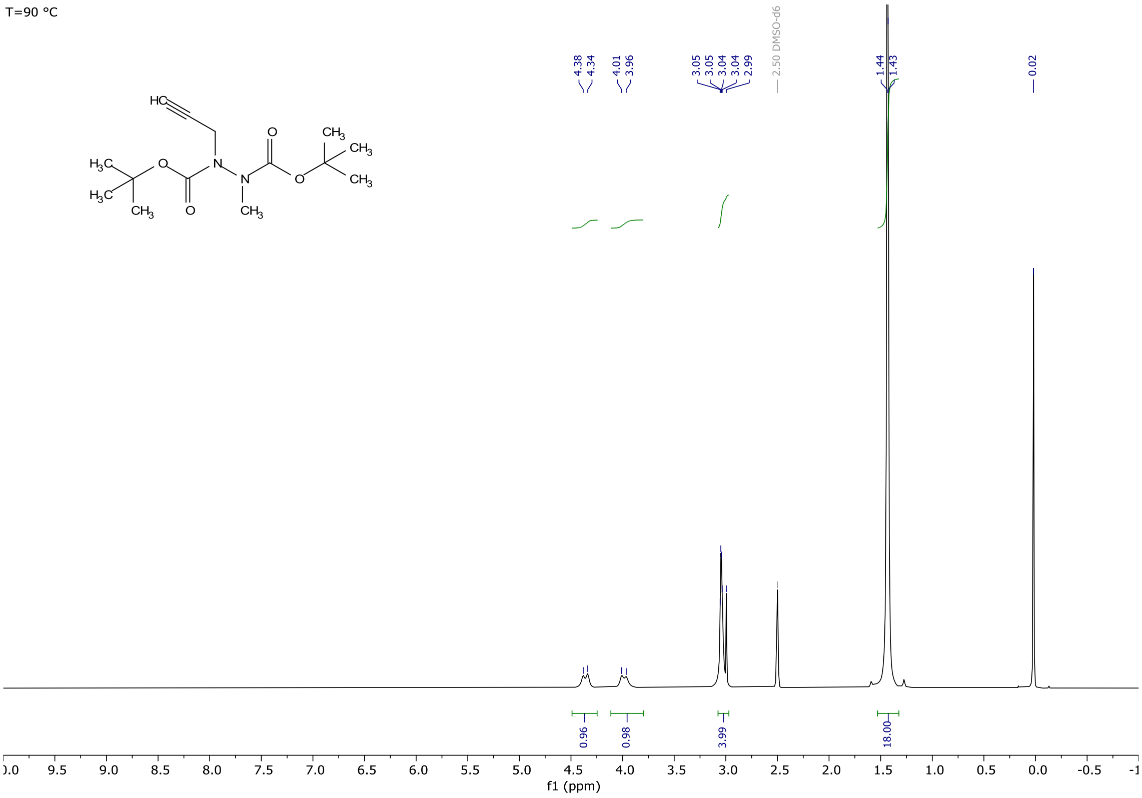

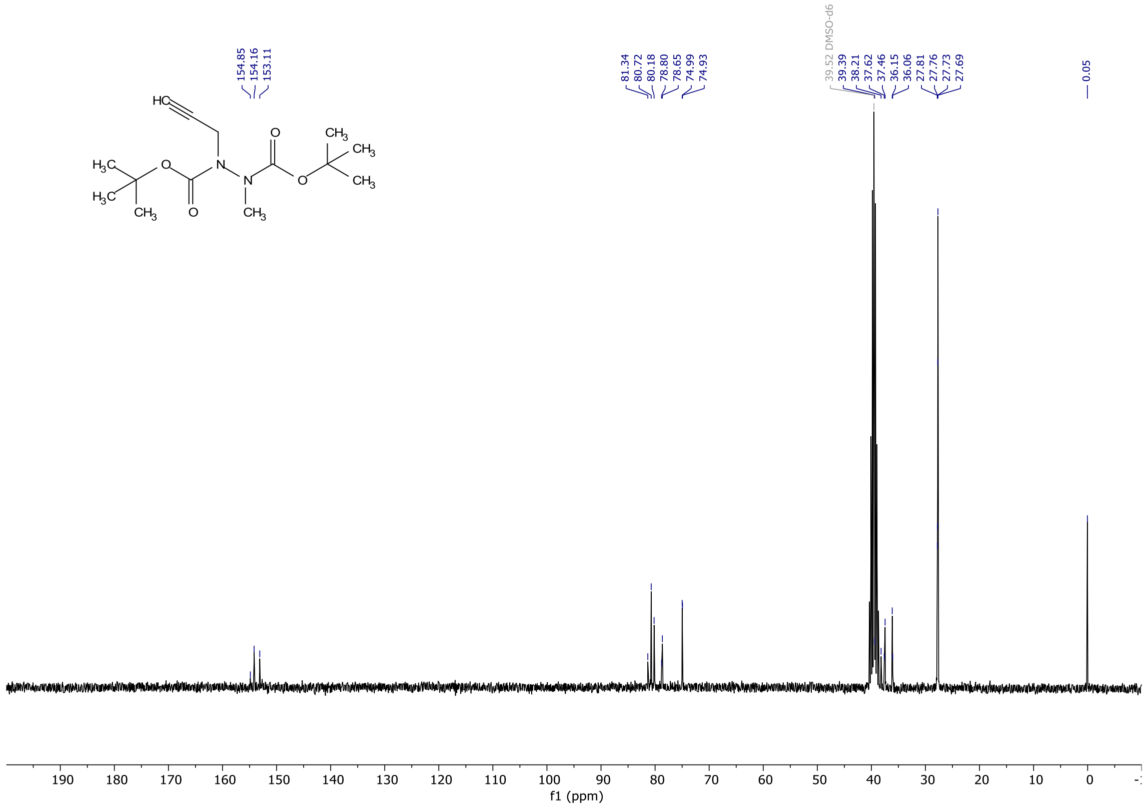

4,5-Dibromo-1-methyl-2-(prop-2-yn-1-yl)-1,2-dihydropyridazine-3,6-dione (3)

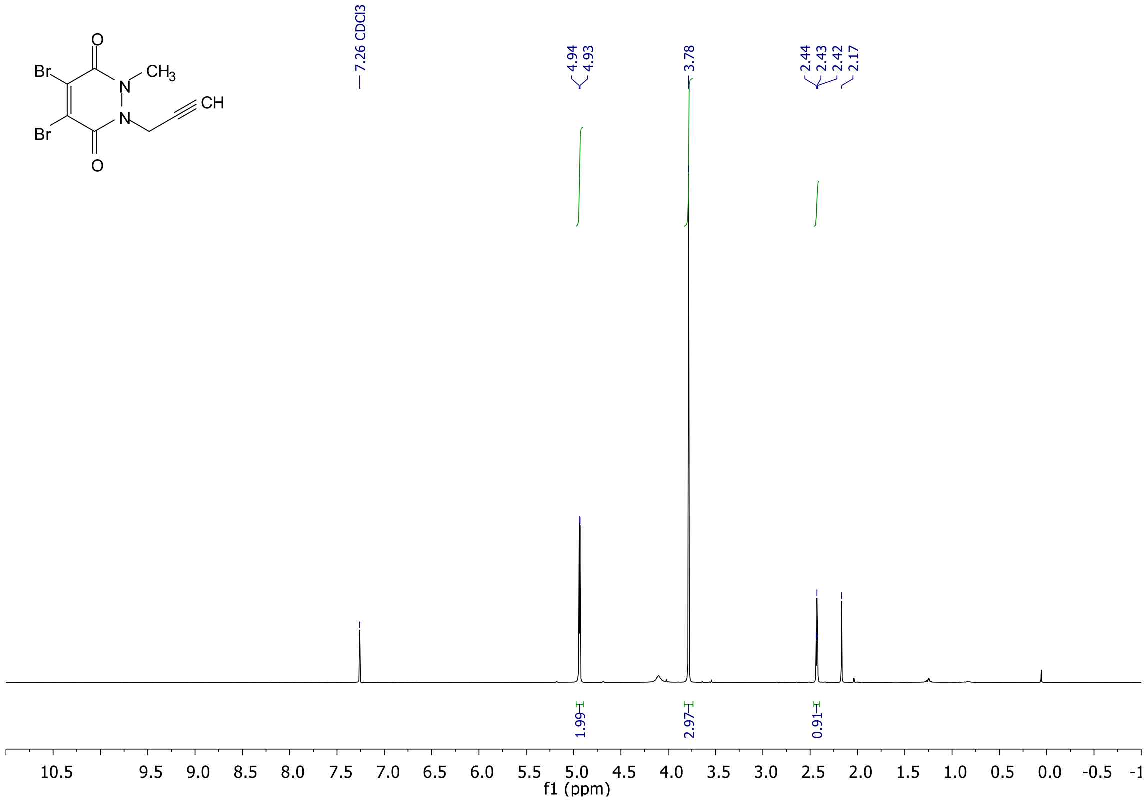

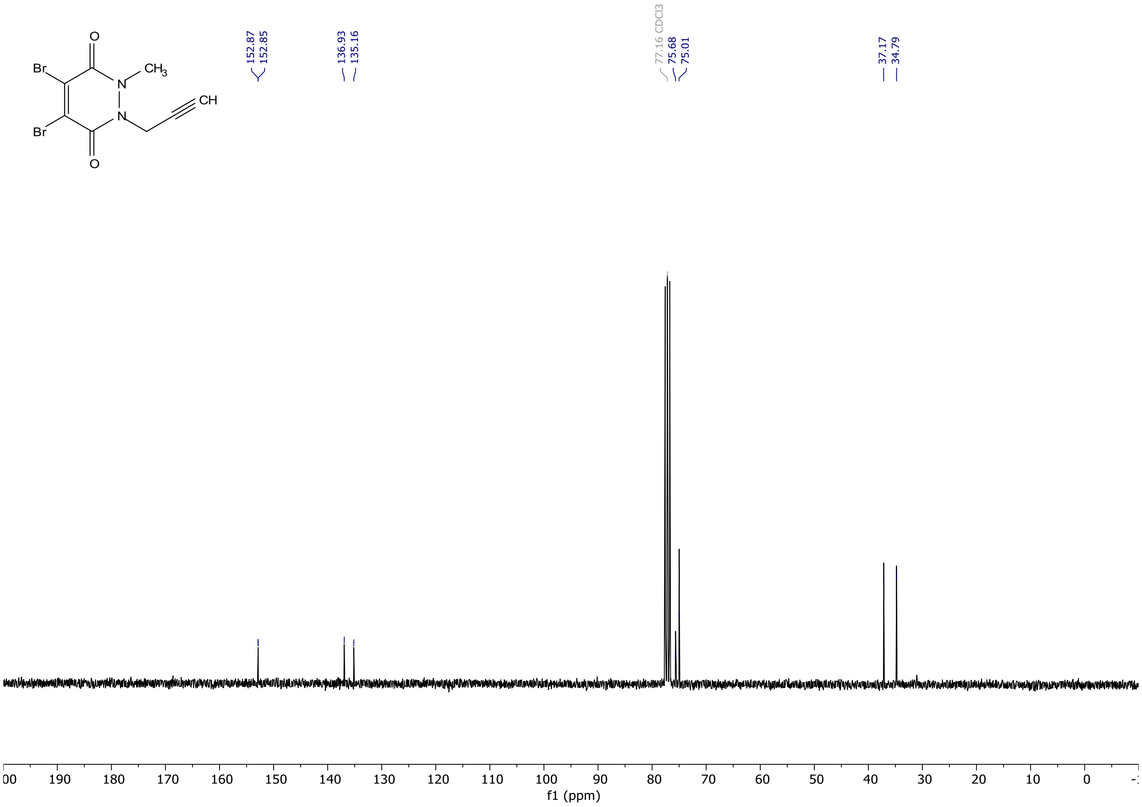

_N_-(10-(4-((2-(2-azidoethoxy)ethyl)carbamoyl)-2-methylphenyl)-7-(dimethylamino)-5,5-dimethyldibenzo[_b_,_e_]silin-3(5_H_)-ylidene)-_N_-methylmethanaminium chloride (4)

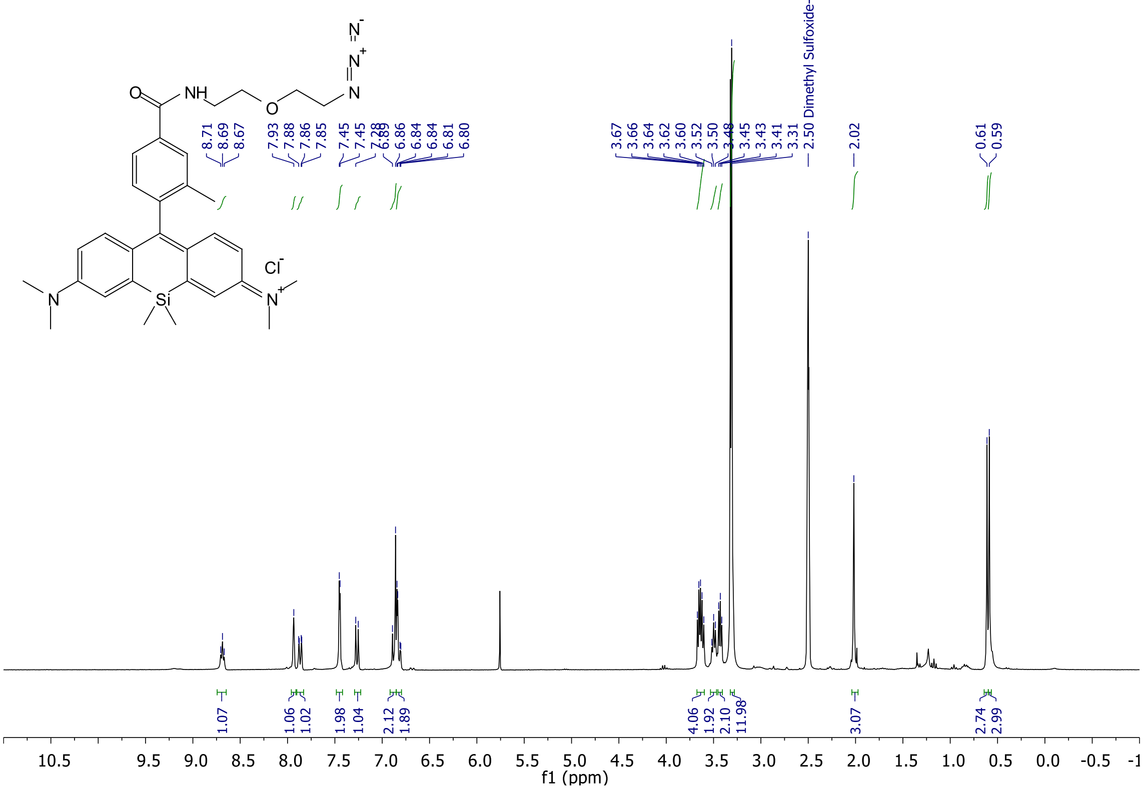

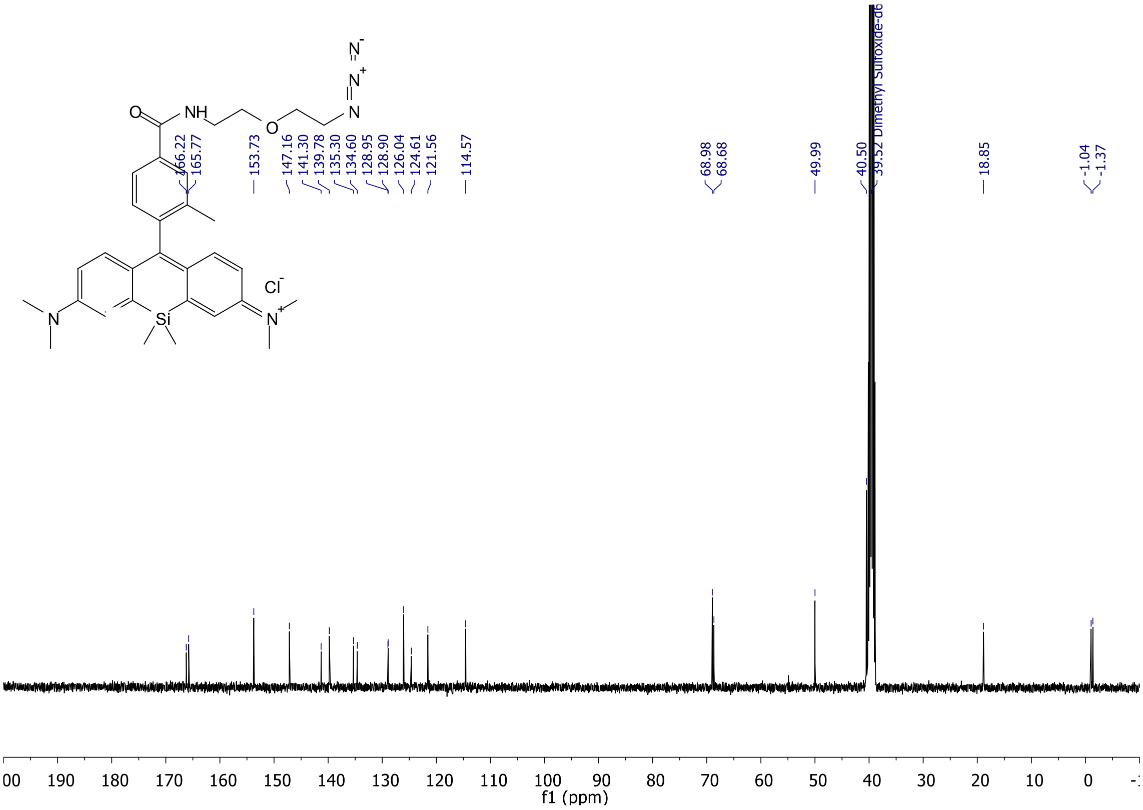

N-(6-azidohexyl)-5,7-difluoro-6-hydroxy-2-oxo-2H-chromene-3-carboxamide (6)

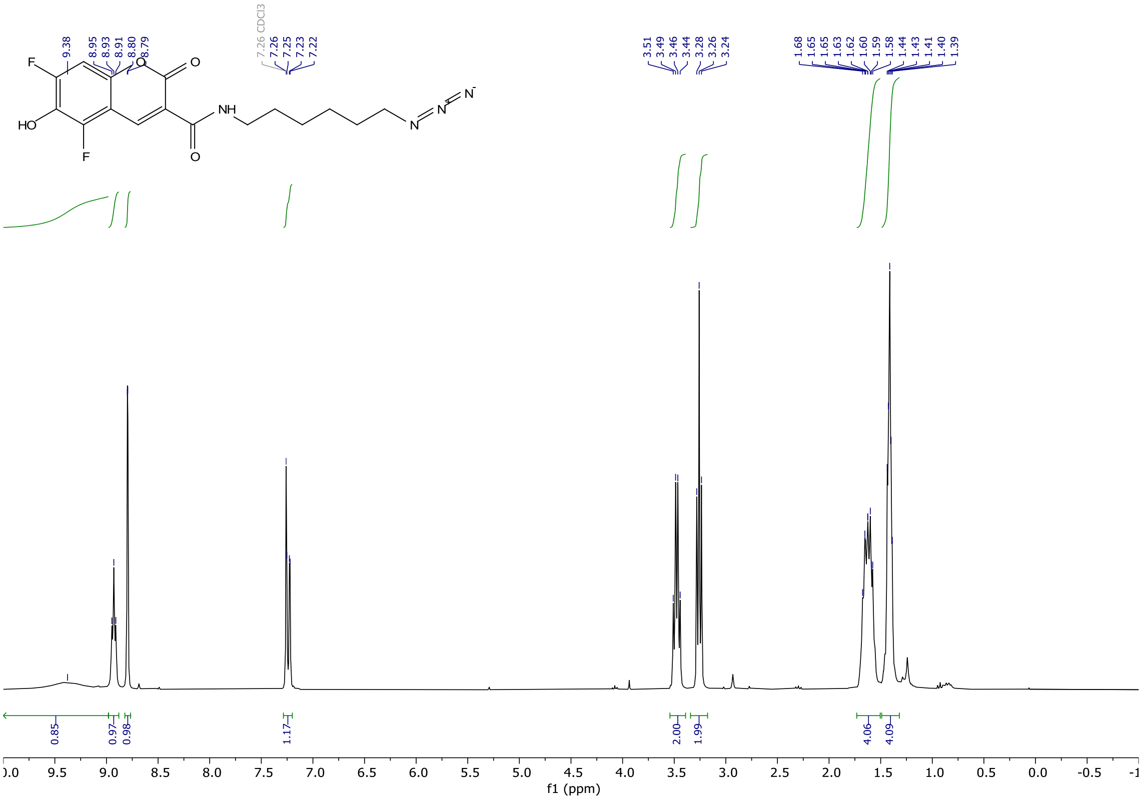

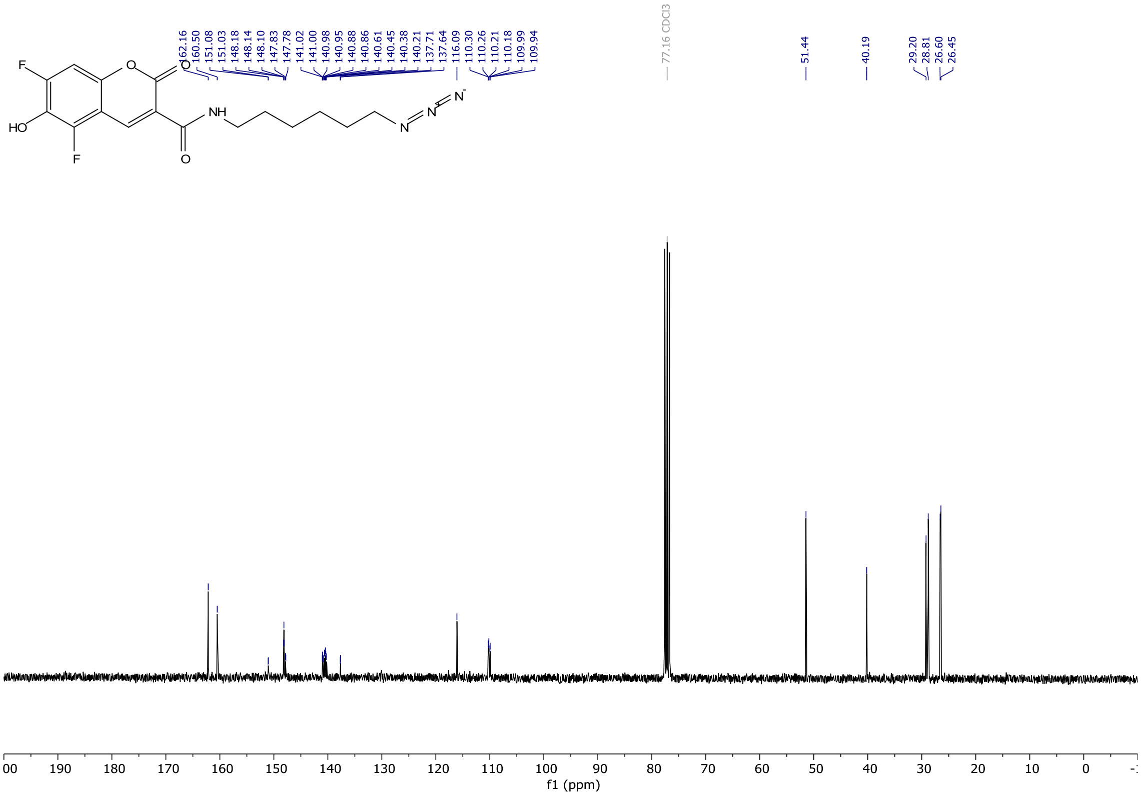

N-(2-(2-(2-(2-azidoethoxy)ethoxy)ethoxy)ethyl)-5-((3a*S*,4*S*,6a*R*)-2-oxohexahydro-1*H*-thieno[3,4-*d*]imidazol-4-yl)pentanamide (7)

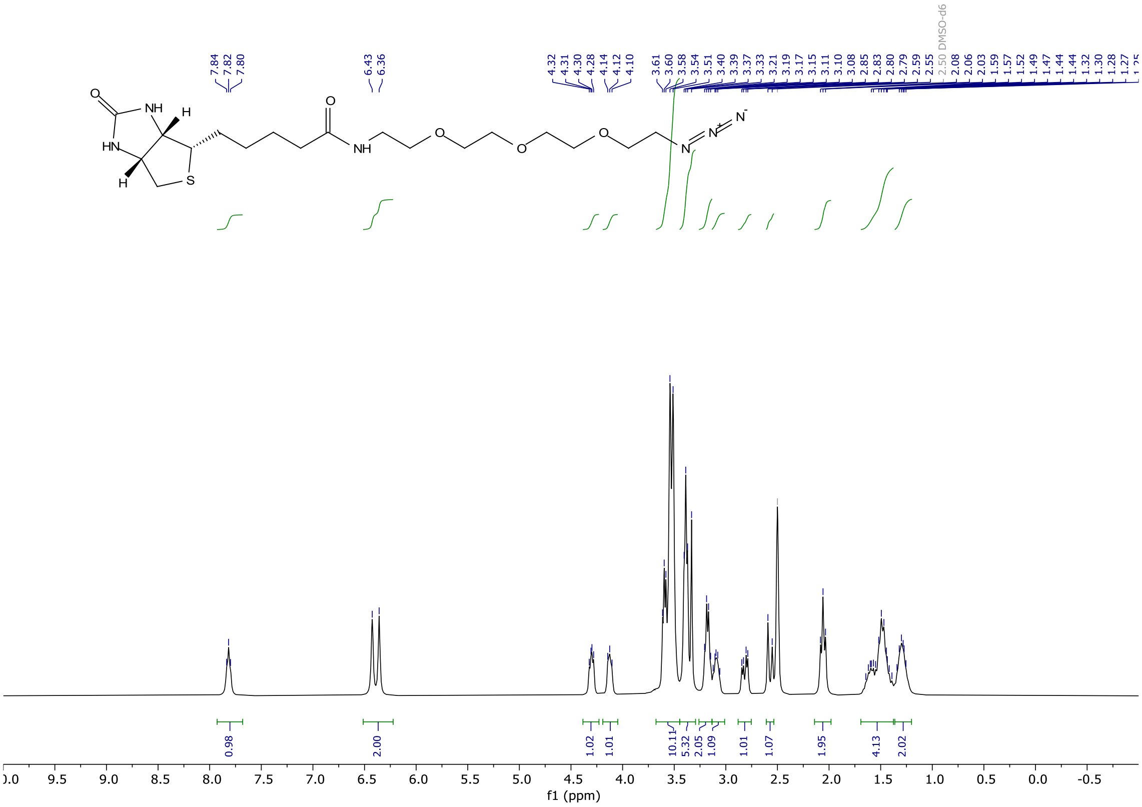

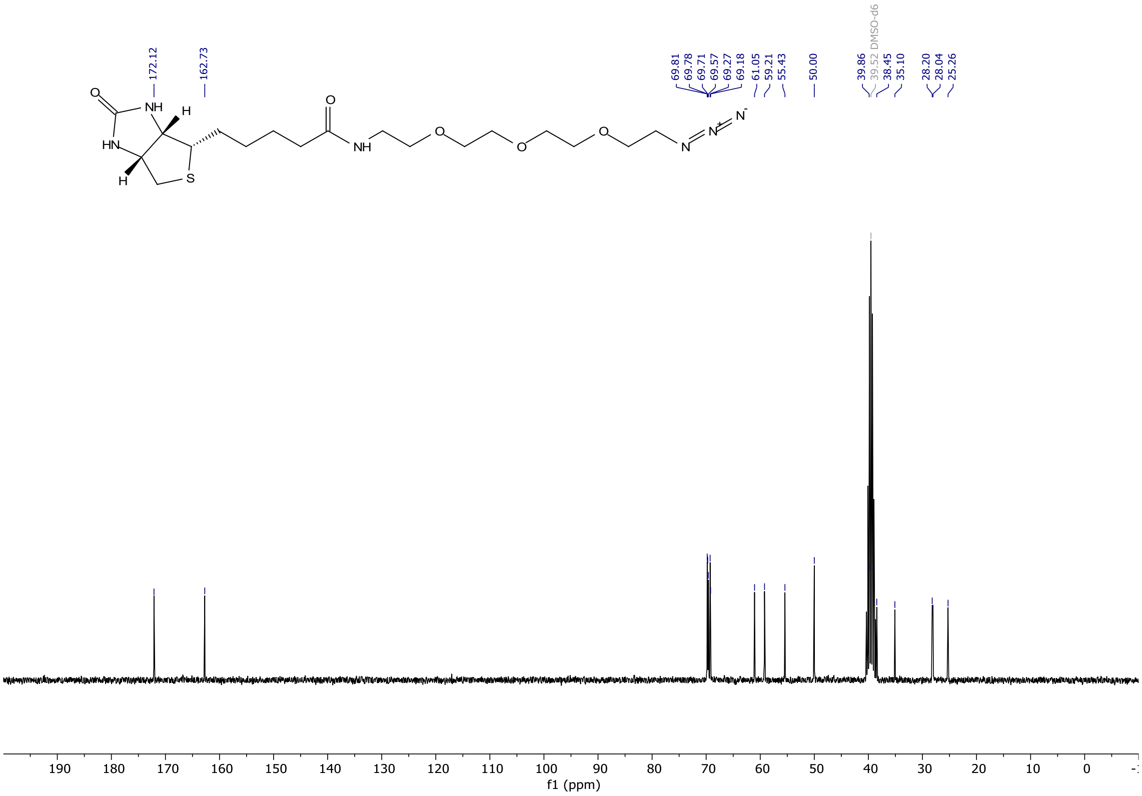

N-(10-(4-((2-(2-(4-((4,5-dibromo-2-methyl-3,6-dioxo-3,6-dihydropyridazin-1(2H)-yl)methyl)-1H-1,2,3-triazol-1-yl)ethoxy)ethyl)carbamoyl)-2-methylphenyl)-7-(dimethylamino)-5,5-dimethyldibenzo[b,e]silin-3(5H)-ylidene)-N-methylmethanaminium chloride (8)

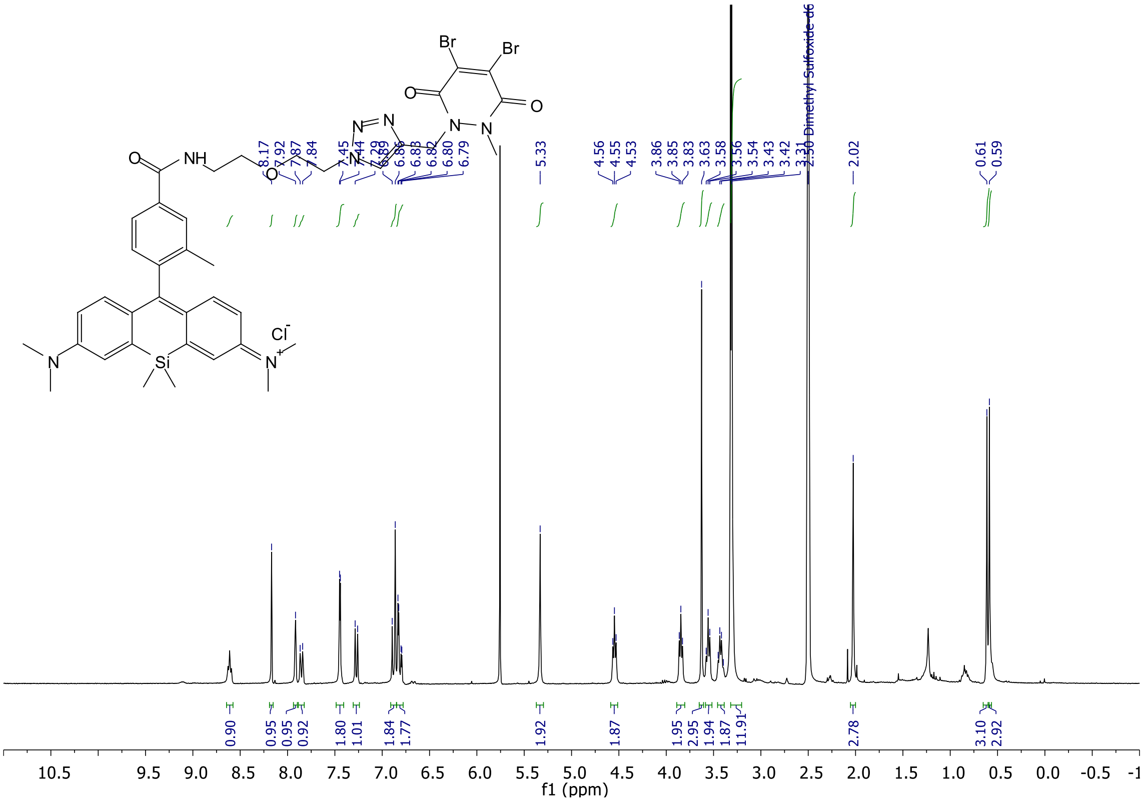

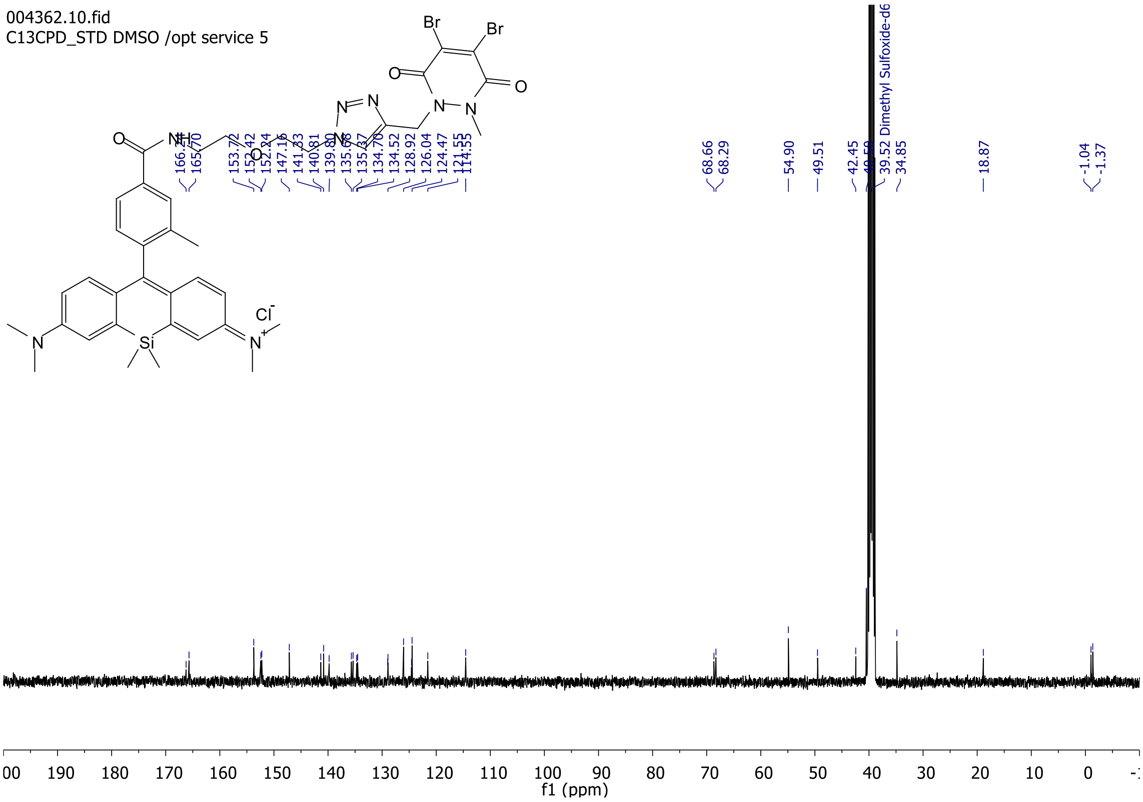

N-(3-(4-((4,5-dibromo-2-methyl-3,6-dioxo-3,6-dihydropyridazin-1(2H)-yl)methyl)-1H-1,2,3-triazol-1-yl)propyl)-3-(5,5-difluoro-7,9-dimethyl-5H-5λ^4,6λ^4-dipyrrolo[1,2-c:2',1'-f][1,3,2]diazaborinin-3-yl)propenamide (9)

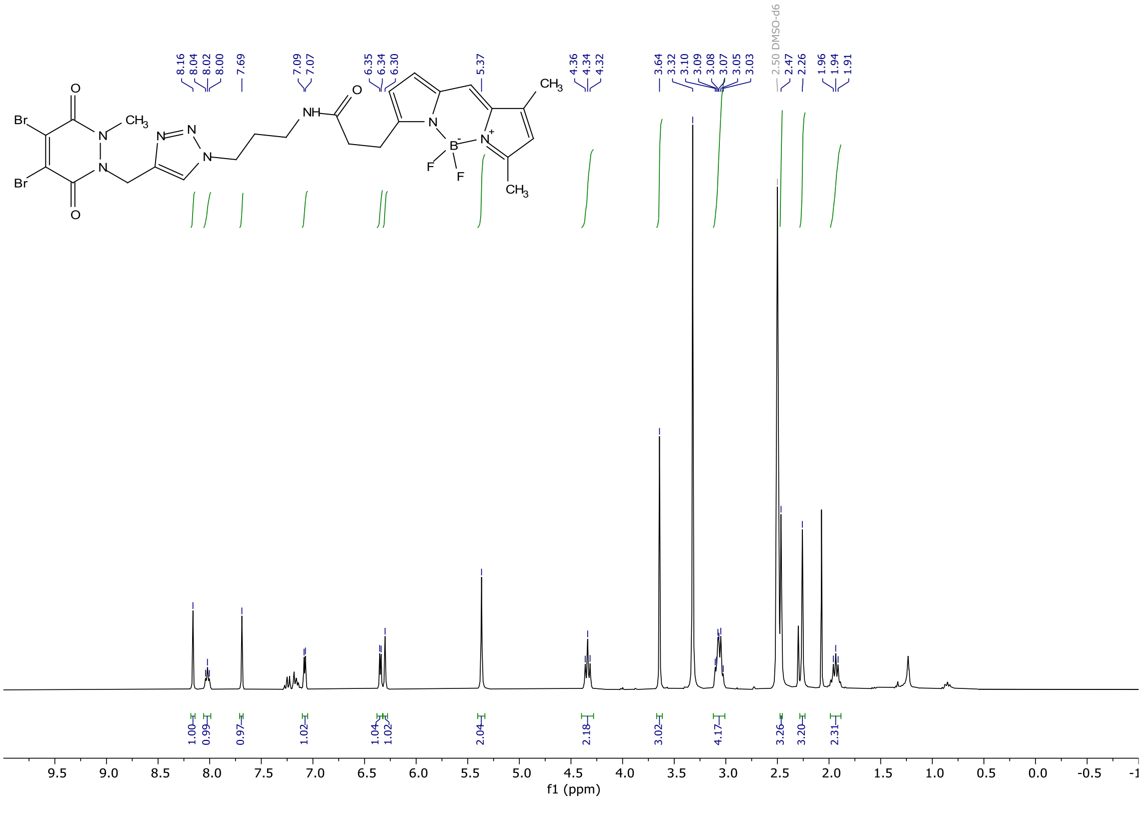

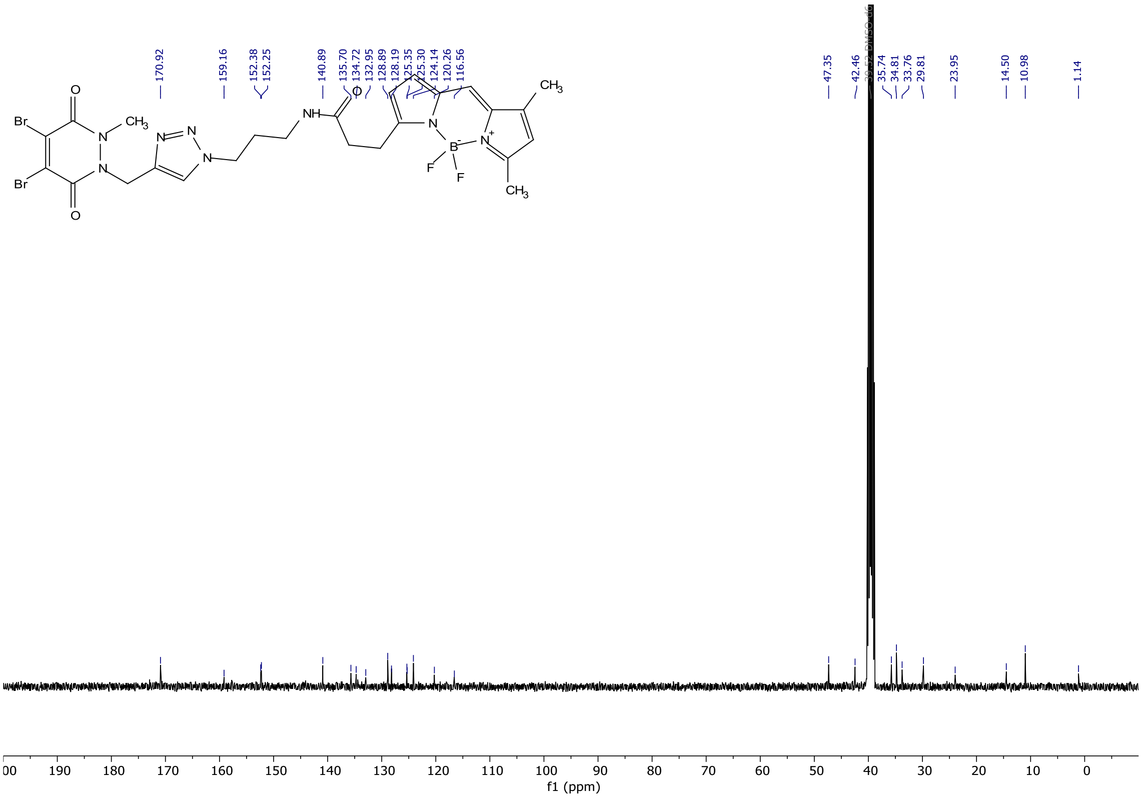
f1 (ppm)

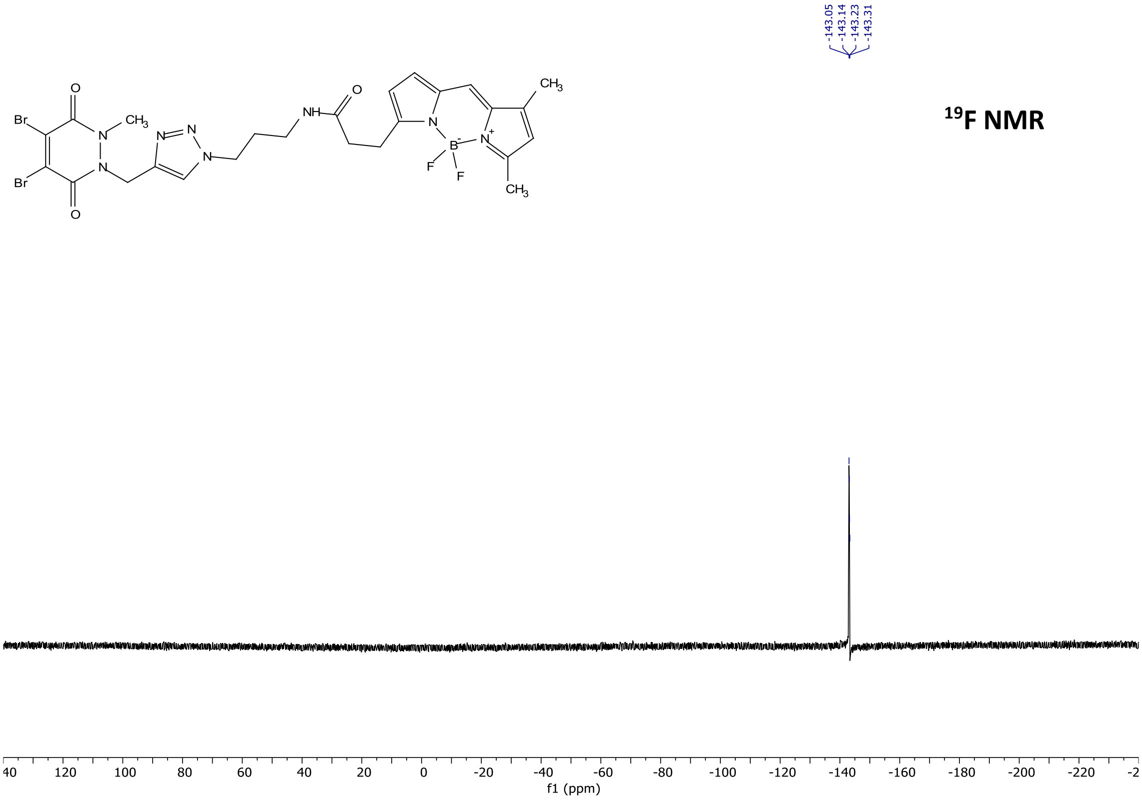
^{19}F NMR
f1 (ppm)

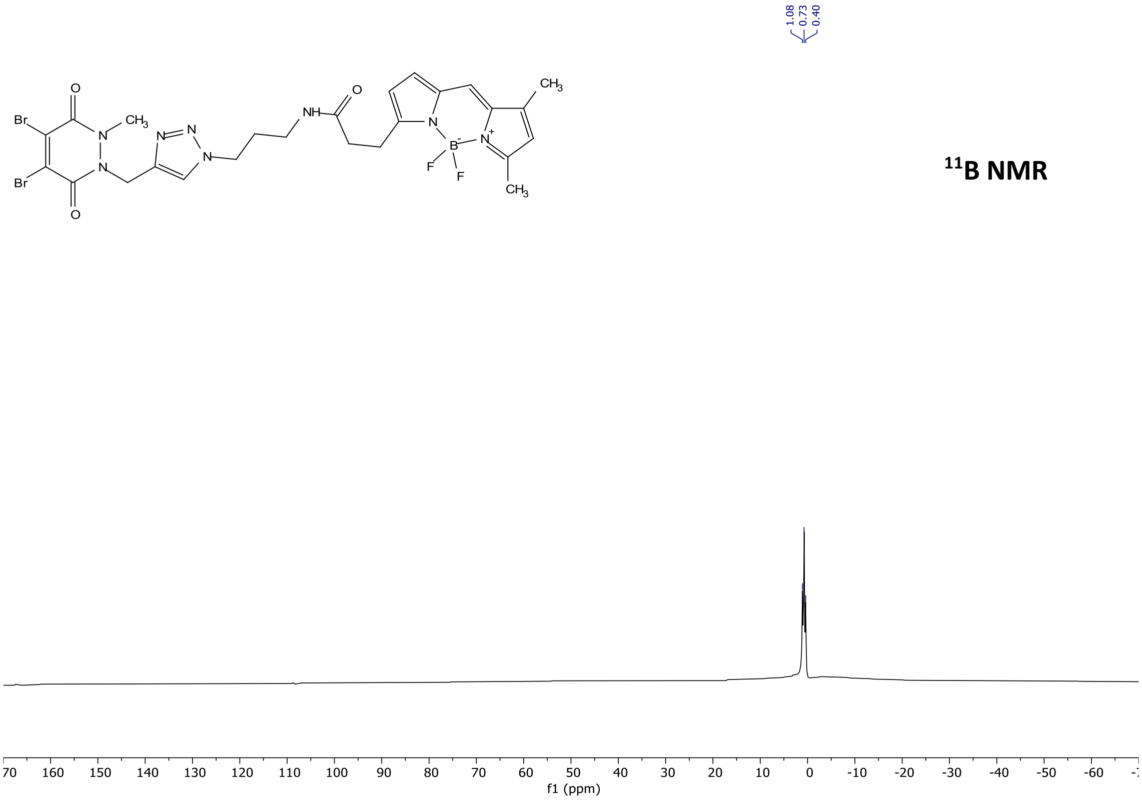

^{11}B NMR

***N*-(6-(4-((4,5-dibromo-2-methyl-3,6-dioxo-3,6-dihydropyridazin-1(2*H*)-yl)methyl)-1*H*-1,2,3-triazol-1-yl)hexyl)-5,7-difluoro-6-hydroxy-2-oxo-2*H*-chromene-3-carboxamide (10)**

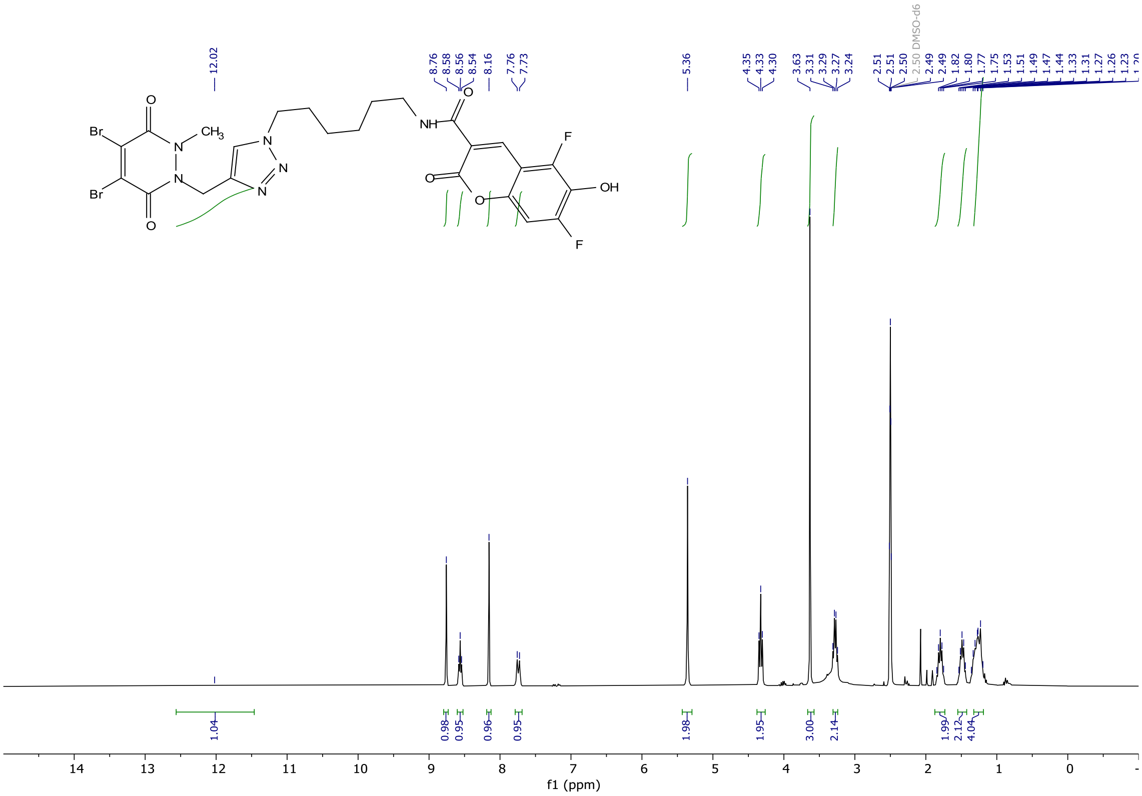

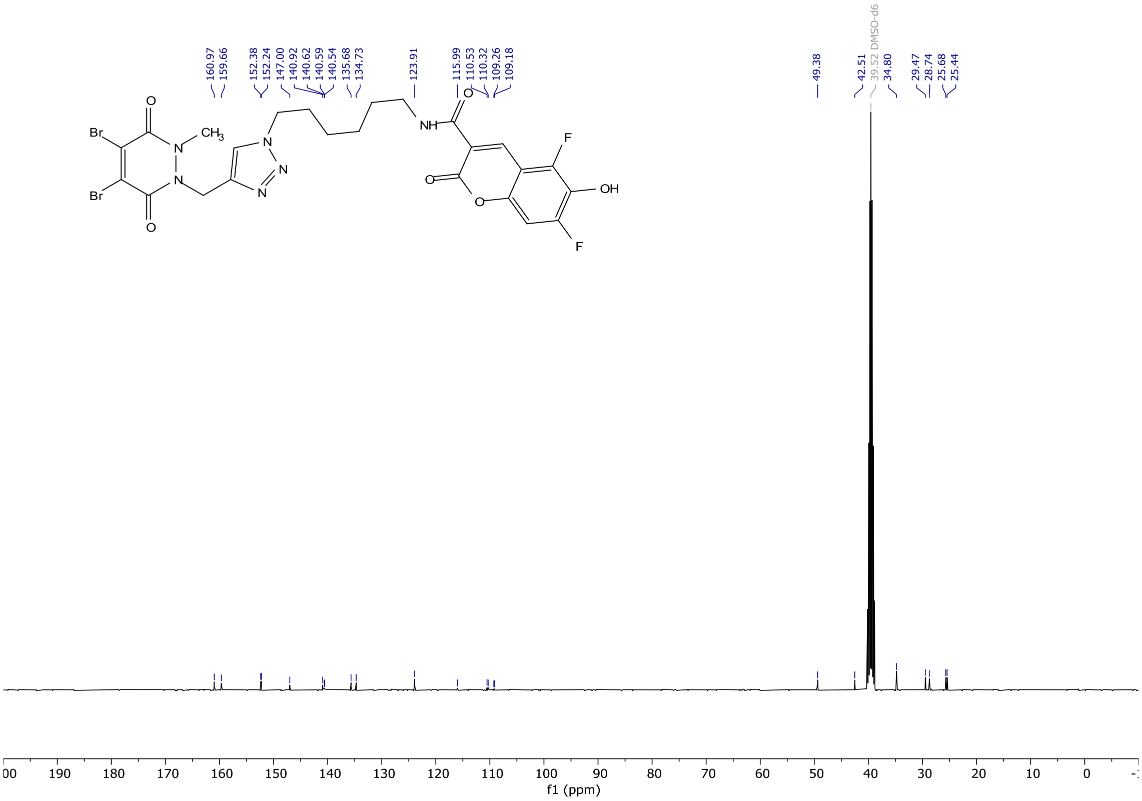
160.97
159.66
152.38
152.24
147.00
140.92
140.62
140.59
140.54
135.68
134.73
123.91
115.99
110.53
110.32
109.26
109.18
49.38
42.51
39.52 DMSO-d6
34.80
29.47
28.74
25.68
25.44
f1 (ppm)

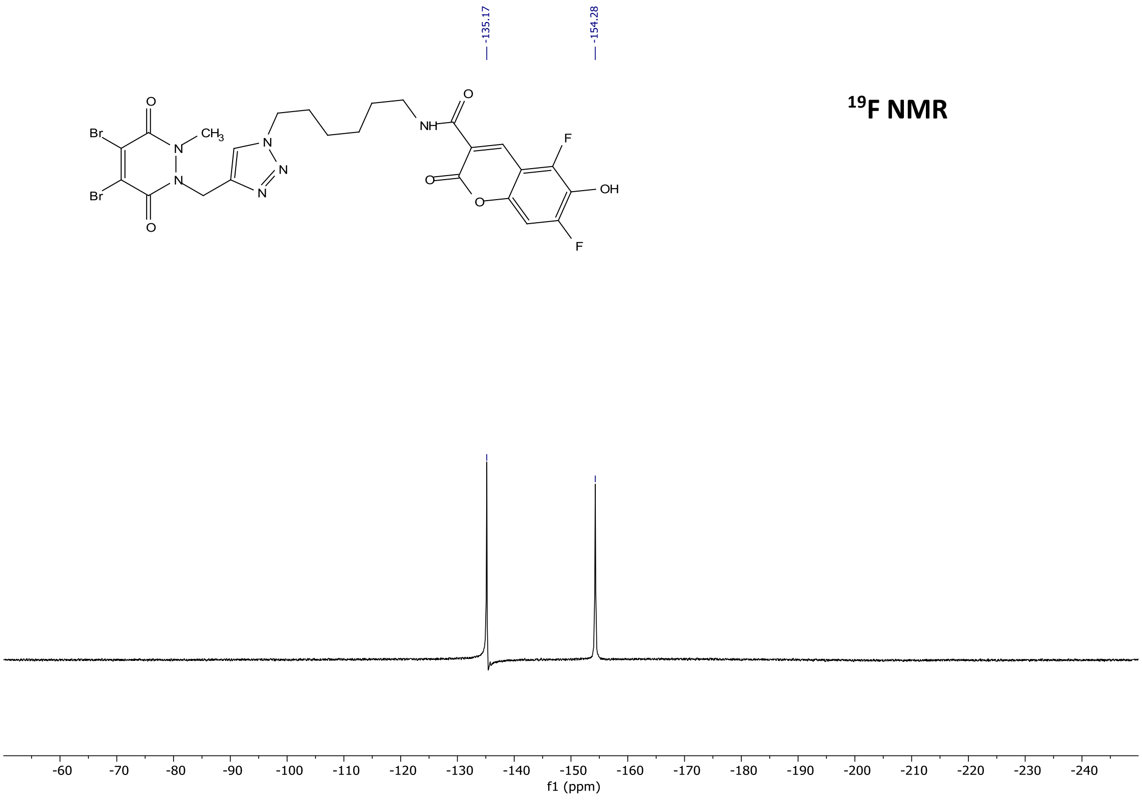
-135.17
-154.28
f1 (ppm)

^{19}F NMR

_N_-(2-(2-(2-(2-(4-((4,5-dibromo-2-methyl-3,6-dioxo-3,6-dihydropyridazin-1(2_H_)-yl)methyl)-1_H_-1,2,3-triazol-1-yl)ethoxy)ethoxy)ethoxy)ethyl)-5-((3a_S_,4_S_,6a_R_)-2-oxohexahydro-1_H_-thieno[3,4-_d_]imidazol-4-yl)pentanamide (11)

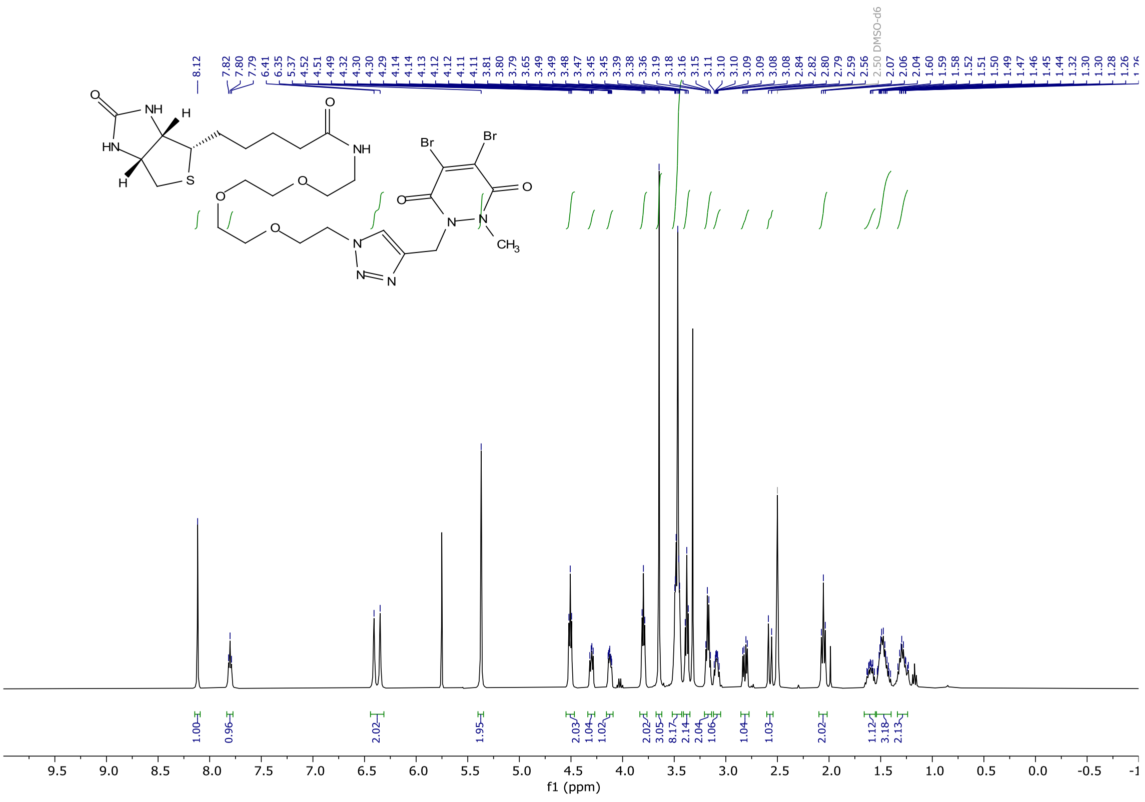

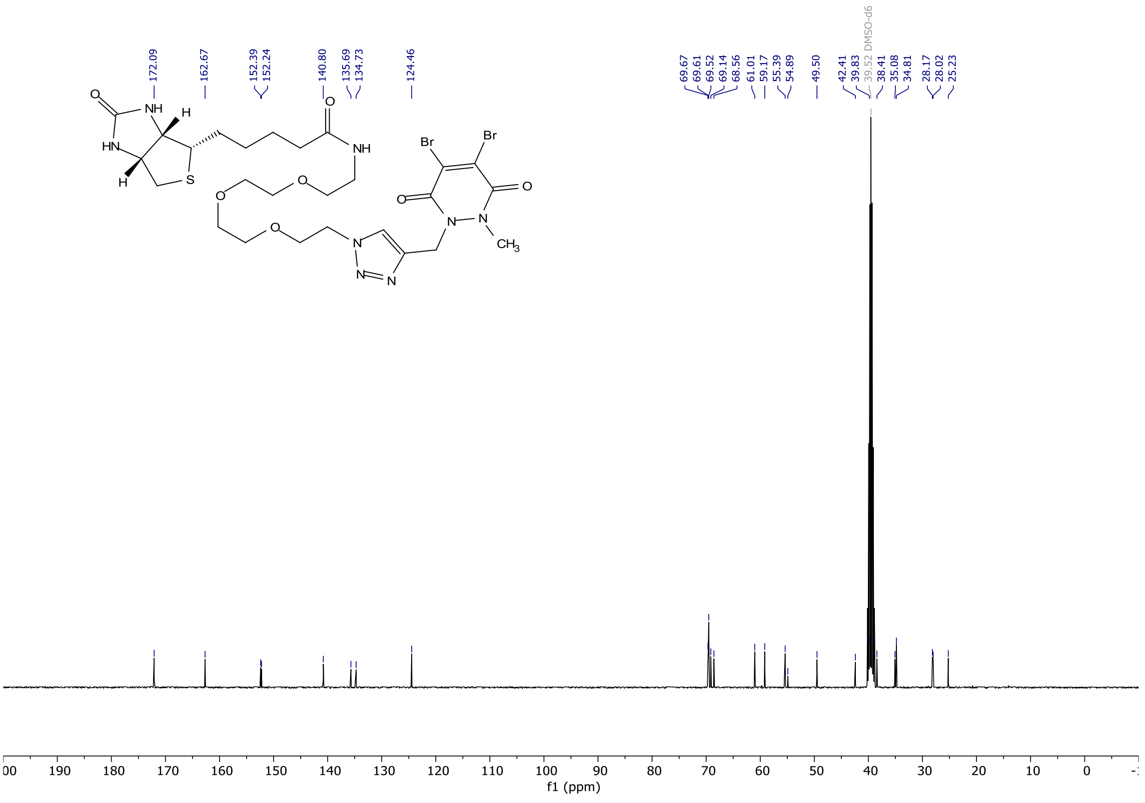

12. Supplementary references

S1 T. Pastierik, P. Šebej, J. Medalová, P. Štacko and P. Klán, *J. Org. Chem.*, 2014, **79**, 3374-3382.

S2 C. Fischer and C. Sparr, *Angew. Chem. Int. Ed.*, 2018, **57**, 2436-2440.

S3 J. B. Grimm, T. A. Brown, A. N. Tkachuk and L. D. Lavis, *ACS Cent. Sci.*, 2017, **3**, 975-985.

S4 A. N. Butkevich, G. Lukinavičius, E. D'Este and S. W. Hell, *J. Am. Chem. Soc*., 2017, **139**, 12378-12381.

S5 R. Sato, J. Kozuka, M. Ueda, R. Mishima, Y. Kumagai, A. Yoshimura, M. Minoshima, S. Mizukami and K. Kikuchi, *J. Am. Chem. Soc.*, 2017, **139**, 17397-17404.

S6 Y. Cui, Y. Li, Q. Duan and T. Kakuchi, *Appl. Biochem. Biotechnol*., 2013, **169**, 239-249.

S7 A. W. Schwabacher, J. W. Lane, M. W. Schiesher, K. M. Leigh and C. W. Johnson, *J. Org. Chem.*, 1998, **63**, 1727-1729.
